# Two-photon sensitive protecting groups operating *via* intramolecular electron transfer: uncaging of GABA and tryptophan†
†Electronic supplementary information (ESI) available: One- and two-photon absorption spectra, emission spectra, synthetic procedures and characterization, square-wave and cyclic voltammetry, photolysis protocols. See DOI: 10.1039/c4sc03775h



**DOI:** 10.1039/c4sc03775h

**Published:** 2015-02-03

**Authors:** Karolina A. Korzycka, Philip M. Bennett, Eduardo Jose Cueto-Diaz, Geoffrey Wicks, Mikhail Drobizhev, Mireille Blanchard-Desce, Aleksander Rebane, Harry L. Anderson

**Affiliations:** a Oxford University , Department of Chemistry , Chemistry Research Laboratory , 12 Mansfield Road , Oxford , OX1 3TA , UK . Email: harry.anderson@chem.ox.ac.uk ; Fax: +44 (0)1865 285002 ; Tel: +44 (0)1865 275704; b Université de Bordeaux , Institut des Sciences Moléculaires , CNRS UMR 5255 , 33400 Bordeaux , France; c Department of Physics , Montana State University , Bozeman , MT 59717 , USA; d National Institute of Chemical Physics and Biophysics , Tallinn 12618 , Estonia

## Abstract

Improved photo-labile protecting groups, with high sensitivity to two-photon excitation, are needed for the controlled release of drugs, as tools in neuroscience and physiology. Here we present a new modular approach to the design of caging groups based on photoinduced electron transfer from an electron-rich two-photon dye to an electron acceptor, followed by scission of an ester to release a carboxylic acid. Three different electron acceptors were tested: nitrobenzyl, phenacyl and pyridinium. The nitrobenzyl system was ineffective, giving only photochemical decomposition and no release of the carboxylic acid. The phenacyl system also performed poorly, liberating the carboxylic acid in 20% chemical yield and 0.2% photochemical yield. The pyridinium system was most successful, and was tested for the release of two carboxylic acids: γ-amino butyric acid (GABA) and tryptophan. The caged GABA undergoes photochemical cleavage with a chemical yield of >95% and a photochemical yield of 1%; it exhibits a two-photon absorption cross section of 1100 GM at 700 nm, corresponding to a two-photon uncaging cross section of 10 ± 3 GM.

## Abstract 2

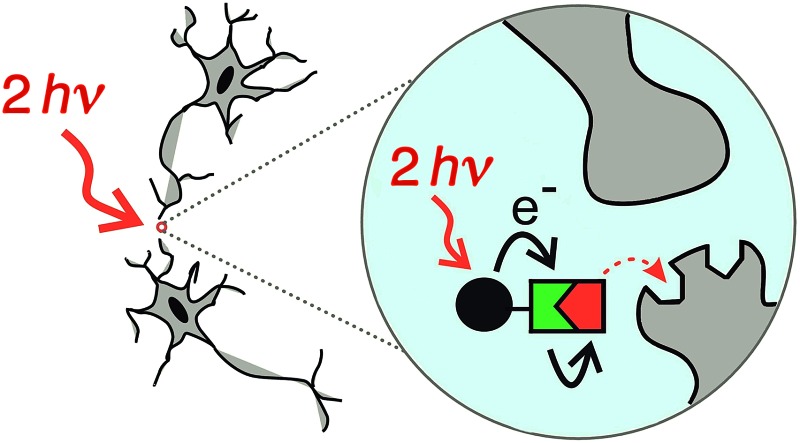
We present a modular approach to photo-labile protecting groups based on photoinduced electron transfer, providing high sensitivity to two-photon excitation.

## Introduction

**Fig. 1:**
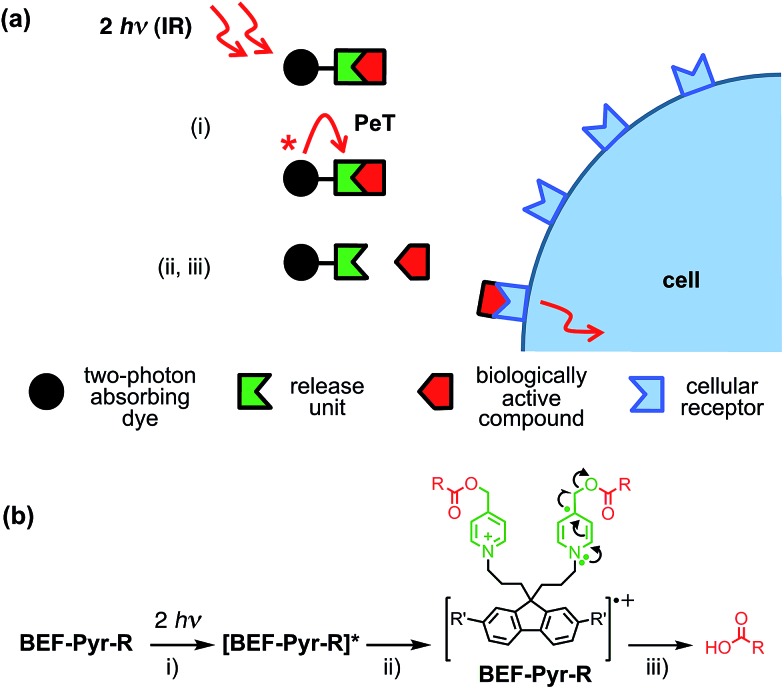
Concept of a photolabile protecting group operating *via* photoinduced electron transfer. Upon two-photon excitation (i) the dye unit donates an electron to the release unit (ii), which undergoes photochemical reaction and liberates the drug (iii).

Light-sensitive protecting groups have continued to gain importance as tools for investigating the role of physiologically active compounds, ever since they were first applied in biological system in 1978.^1^ A number of UV- and visible-light cleavable cages have been developed^2^ to allow rapid spatially and temporally controlled photo-release of various biomolecules within living cells.^3^ In the 1990s, the concept of uncaging was extended to take advantage of two-photon absorption (TPA),^4,5^ a nonlinear optical phenomenon in which excitation occurs by the simultaneous absorption of two photons, each having half the energy of the corresponding one-photon process. The main advantage of TPA is that excitation is effectively restricted to the focal volume, giving tight spatial control. Furthermore, TPA allows the use of near-IR wavelengths which diminishes photo-damage and improves tissue penetration by reducing scattering and avoiding absorption by natural pigments. Unfortunately, most protecting groups optimized for one-photon uncaging display low efficiency under two-photon excitation, because their chromophores lack the specific features required for efficient TPA. The process of uncaging can be divided into two steps: light absorption followed by bond scission. The efficiency of TPA is quantified by the TPA cross-section (*δ*_a_), while the quantum yield of uncaging (*φ*_u_) measures the efficiency of photo-induced bond scission. The product of these two parameters, the two-photon uncaging cross-section (*δ*_u_ = *δ*_a_ *φ*_u_) is a figure of merit reflecting the overall sensitivity of a protecting group to two-photon uncaging. Typically, *δ*_u_ values reported to date lie between 0.05 and 2.5 GM (ref. 2*c*, 6, 7) (1 GM = 10^50^ cm^4^ s per photon) whereas *δ*_u_ > 3 GM is desired for efficient uncaging in living cells.^8^ A common strategy for enhancing the TPA cross-section of a pre-existing protecting platform is to extend the π-system and modulate the strength of electron donating/accepting substituents. Dipolar, quadrupolar and octupolar architectures have been explored for functionalizing established caging groups such as coumarine,^9^ *o*-nitrobenzyl,^10,11c^ 2-(*o*-nitrophenyl)propyl,^11^ phenacyl^11*c*^ and quinoline^12^ with vinyl, phenyl, styryl, dihydronaphthalenyl, thienyl, fluorenyl and triphenylamine groups. The strategy of incorporating an existing protecting group into a conjugated donor–π–acceptor system led to a dipolar protecting group within the 2-(*o*-nitrophenyl)propyl series with a record *δ*_u_ of 11 GM at 800 nm.^11*a*^ However, as absorption and bond scission are inherently related, any alteration in the caging group influences both *δ*_a_ and *φ*_u_. Therefore some structurally modified cages, such as BNSF (2,7-bis-{4-nitro-8-[3-(2-propyl)-styryl]}-9,9-bis-[1-(3,6-dioxaheptyl)]-fluorene, *δ*_u_ of 5 GM at 800 nm, 65% yield of uncaging),^11*b*^ suffer from light-induced side reactions or decreased yield of release, compared to their parent protecting units. Since it proves extremely difficult to enhance *δ*_a_ while preserving a high value of *δ*_u_, several attempts have been made to explore an alternative approach, in which the absorption and release steps are decoupled and occur in different parts of the caging platform. A modular design allows each process to be optimized independently. This concept was first demonstrated for one-photon photolysis, where two spatially separated steps of uncaging were linked by intramolecular photoinduced electron transfer (PeT)^13^ or triplet sensitization.^14^ Recently this strategy has been implemented in two-photon uncaging systems: the fluorenyl–nitroindolinyl derived protecting group (*δ*_u_ of 0.5 GM at 730 nm), where the absorption step is followed by intramolecular energy transfer-mediated release^15^ and 2-(*o*-nitrophenyl)propyl–thioxanthone with intermolecular FRET (*δ*_u_ of 0.86 GM at 766 nm).^16^ Here, we report a study of PeT-mediated uncaging in a two-photon excitable system.^17^ Drawing upon previously reported designs,^13^ we devised a protecting group, the removal of which operates *via* intramolecular PeT between a photoexcited electron-donor (a TPA dye with high *δ*_a_) and an electron-acceptor (pre-existing release unit) to achieve efficient release of physiologically active compounds (Fig. 1a).

**Fig. 2:**
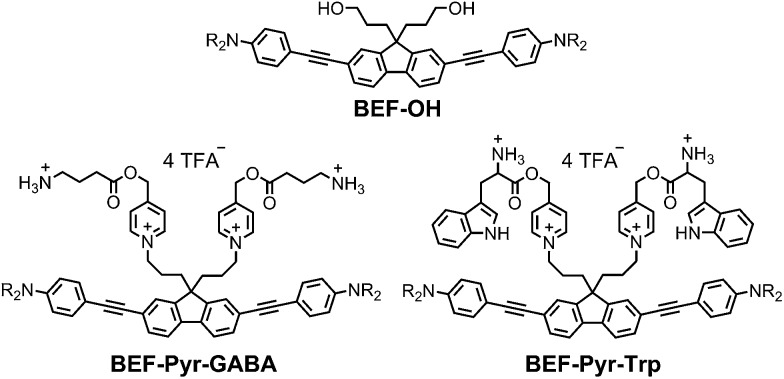
Structure of model electron-donor **BEF-OH**, caged tryptophan, **BEF-Pyr-Trp**, and caged GABA, **BEF-Pyr-GABA**; R = (CH_2_CH_2_O)_7_CH_3_.

Our studies began with choosing a suitable electron-donor with high *δ*_a_. We selected a symmetric banana-shaped bisethynyl fluorene (BEF) dye, in which the core is extended with substituted anilines *via* acetylene bridges.^18^ A pyridinium salt was chosen as a potential electron-acceptor, since it has been demonstrated to release carboxylic acids upon PeT.^13b,c,19^ Fig. 1b shows the proposed mechanism of photo-deprotection. Light absorption generates a photosensitizer-based singlet state, which is quenched by electron transfer to the release group. The resulting charge-shifted state decays by σ-bond cleavage to liberate the physiologically active carboxylic acid. The symmetrical design was chosen to simplify the synthesis, while the aniline unit was substituted with heptaethyleneglycol chains to promote solubility in aqueous media. A model dye-unit **BEF-OH** was synthesized for the purpose of photophysical and electrochemical studies (Fig. 2). We used caged tryptophan, **BEF-Pyr-Trp**, for testing the intramolecular PeT mediated uncaging mechanism. l-Tryptophan (Trp) was selected as a model amino acid due to the presence of an indole chromophore that allows release to be quantified by HPLC, with UV detection. We also prepared caged γ-amino butyric acid (GABA), **BEF-Pyr-GABA**, to explore the utility of our protecting group for release of an inhibitory neurotransmitter.

## Results and discussion

### Synthesis

**Scheme 1:**
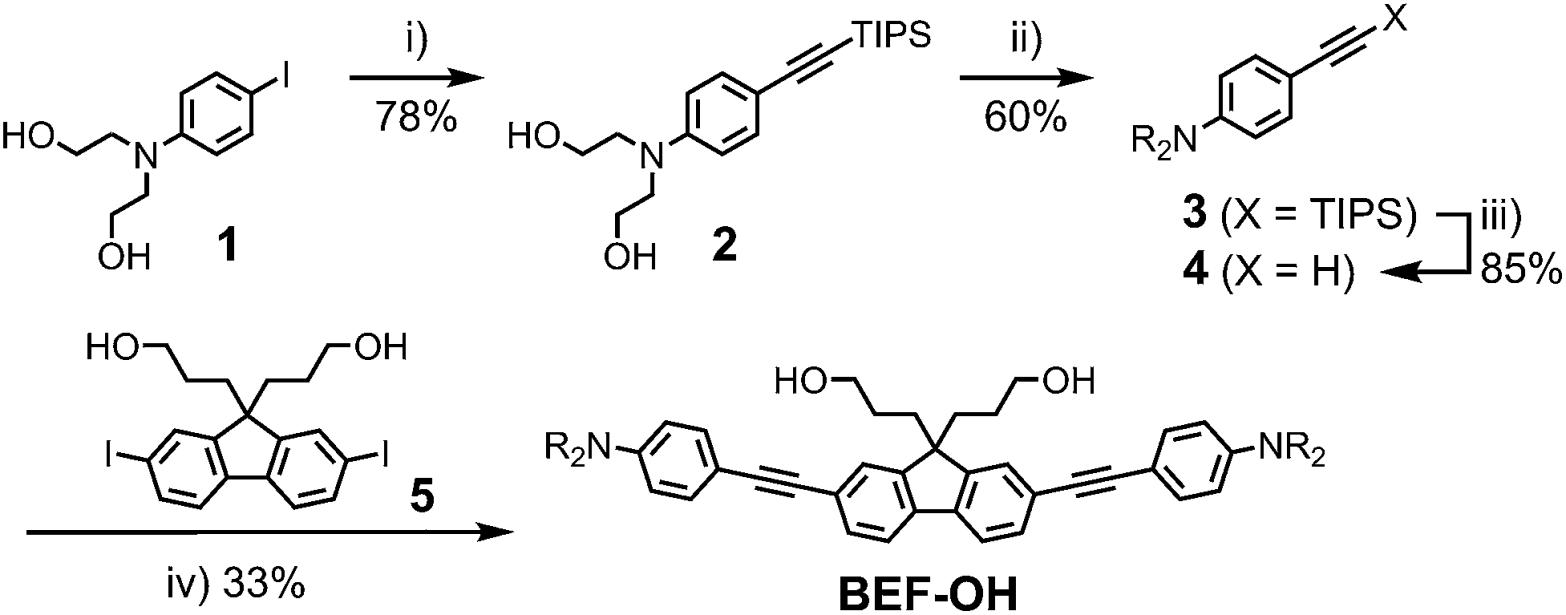
Reagents: (i) TIPS-acetylene, Pd(OAc)_2_, PPh_3_, CuI, DIPA, 50 °C, 16 h, (ii) CH_3_(OCH_2_CH_2_)_6_OTs, NaH, THF, reflux, 48 h, (iii) TBAF, THF, 20 °C, 12 h, (iv) **4**, Pd(OAc)_2_, PPh_3_, CuI, DIPA, MeCN, 20 °C, 3 h; Ts = *p*-toluenesulfonate, TIPS = triisopropylsilyl, THF = tetrahydrofuran, DIPA = diisopropylamine, TBAF = tetra-*n*-butylammonium fluoride, R = (CH_2_CH_2_O)_7_CH_3_.

**Scheme 2:**
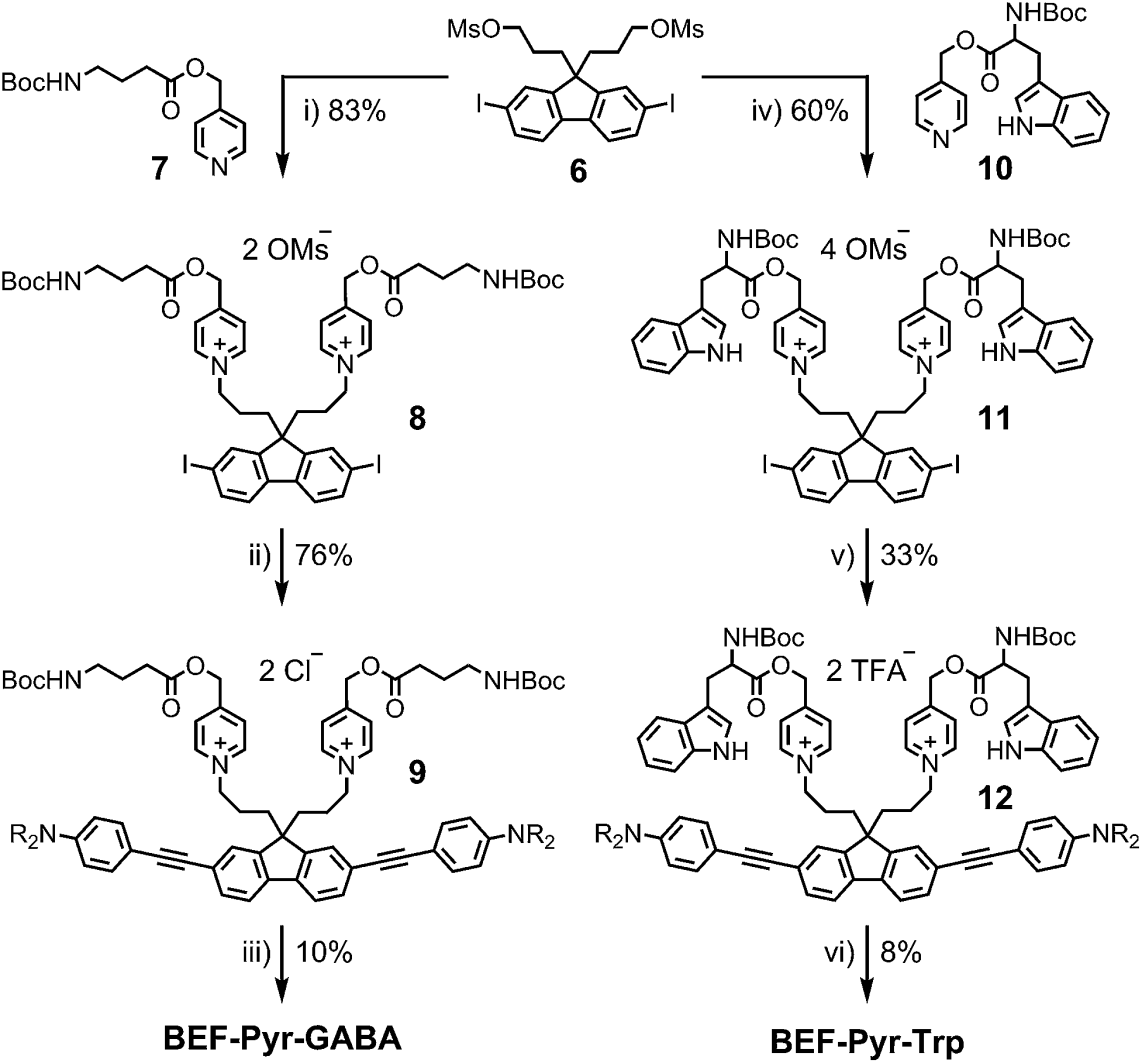
Reagents: (i) MeCN, reflux, 36 h, (ii) **4**, Pd(OAc)_2_, PPh_3_, CuI, DIPA, 20 °C, 1 h, brine wash, (iii) BF_3_·OEt_2_, DCM, 0 °C → 20 °C, 5 h, (iv) MeCN, 100 °C, 24 h, (v) **4**, Pd(OAc)_2_, PPh_3_, CuI, DIPA, DCM, MeCN, 20 °C, 2 h, then purification in TFA-buffered solvent, (vi) HCO_2_H, TIPS-H, 20 °C, 1 h.

The reference dye **BEF-OH** was prepared as shown in Scheme 1. The synthesis started from *N*-(4-iodophenyly)diethanolamine^20^ (**1**) by coupling with TIPS-acetylene to give intermediate **2**, which was subsequently alkylated with hexaethyleneglycol monomethyl ether tosylate. Removal of TIPS group from **3** resulted in the key aniline intermediate **4**, which was coupled with the central fluorene diiodide **5** (see ESI†) under Sonogashira conditions to give **BEF-OH**. In a convergent synthesis towards GABA and tryptophan derivatives, the fluorene core was appended with the pyridinium esters before conjugation with the substituted aniline units **4** using Sonogashira cross-coupling (Scheme 2). The final step involved removal of the Boc protecting groups to yield the trifluoroacetate salts **BEF-Pyr-GABA** and **BEF-Pyr-Trp**.

### One- and two-photon absorption and fluorescence spectra

**Fig. 3:**
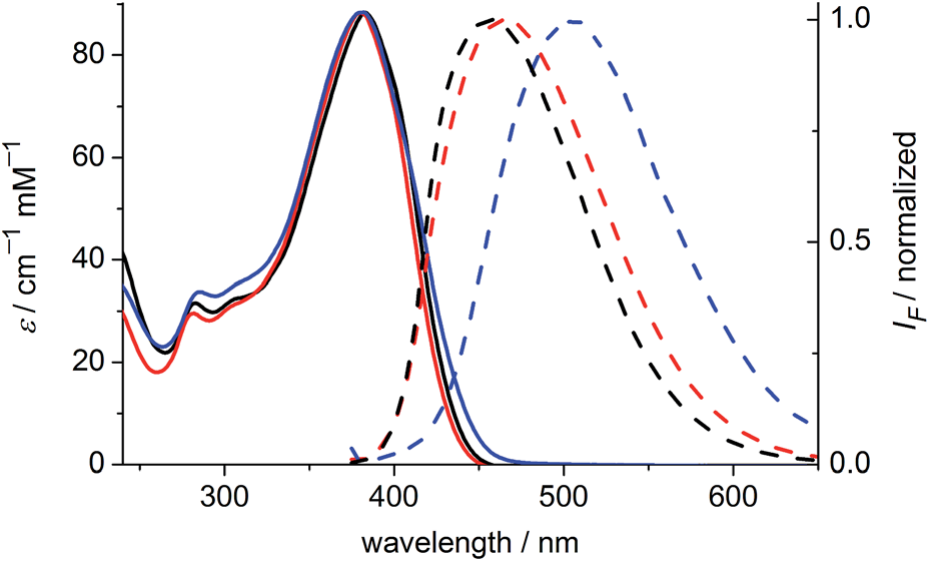
Absorption (solid) and emission (dashed) spectra of **BEF-OH** in THF (black), EtOH (red) and water (blue). Excitation wavelength: 366 nm.

**Fig. 4:**
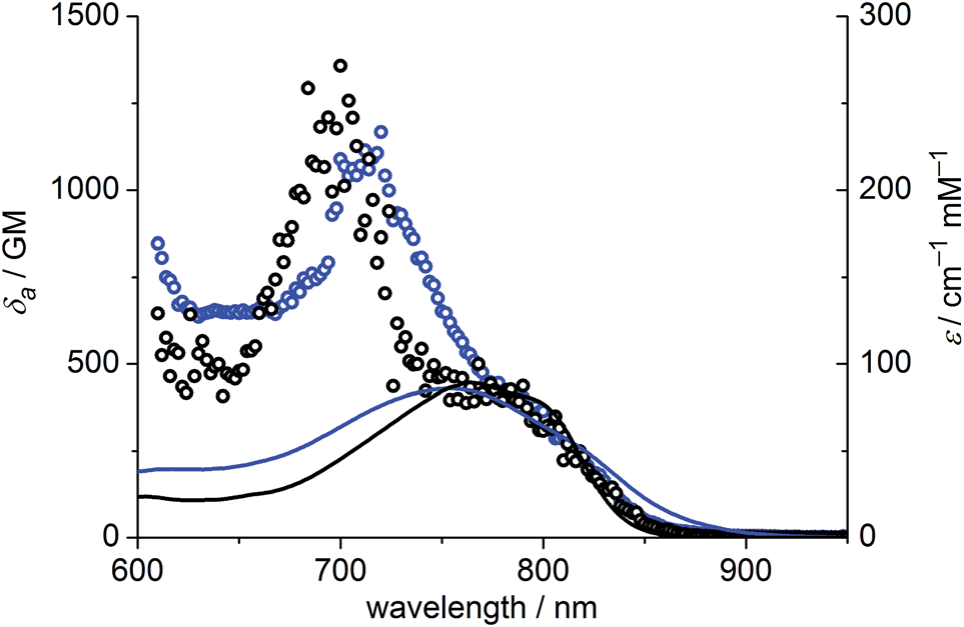
TPA spectra (circles) overlaid with double-wavelength one-photon absorption spectra (lines); blue – **BEF-OH** recorded in water, black – **BEF-OTBDMS** (*t*-butyldimethylsilyl ether derivative) recorded in EtOH.

The one-photon absorption and emission spectra of **BEF-OH** in water and polar organic solvents (EtOH and THF) are shown in Fig. 3. The fluorene-based dye exhibits a strong absorption at 300–400 nm (maximum: 380 nm; *ε*_380_ = 9.0 × 10^4^ M^–1^ cm^–1^). Comparison of the absorption spectra in water and organic solvents reveals that the one-photon absorption properties of **BEF-OH** are independent of the solvent polarity. However, the fluorescence spectrum is sensitive to the environment, being red-shifted and broadened in polar solvents. The Stokes shifts are 78, 83 and 128 nm in THF, EtOH and water, respectively. Bathochromic shifts in the luminescence spectra of banana-shaped fluorene-dyes have previously been attributed to symmetry breaking and the formation of a polar excited state, which is stabilized in polar solvents.^21^ The fluorescence quantum yield (*φ*_f_) of **BEF-OH** is about 0.42 in organic solvents (0.43 in THF, 0.41 in EtOH, referenced to quinine in 0.5 M H_2_SO_4_), but it falls to 0.1 in water. The TPA spectra of **BEF-OH** and its *t*-butyldimethylsilyl ether derivative, **BEF-OTBDMS** (Fig. S28†), in water and EtOH, respectively, are compared in Fig. 4. The TPA maxima are 1150 GM at 700 nm (for **BEF-OTBDMS** in EtOH) and 1100 GM at 715 nm (for **BEF-OH** in water). These cross-sections are similar to those reported previously for closely related dyes.^18,22^ The spectrum is slightly broader and red-shifted in water, but the spectra are similar, revealing that the TPA is insensitive to the solvent environment. In both solvents, there is a shoulder in the TPA spectrum at twice the wavelength of the one-photon allowed S_0_ → S_1_ transition. However, the TPA spectra are dominated by peaks corresponding to the one-photon forbidden, two-photon allowed higher-energy electronic or vibronic transitions. This behavior is similar to that reported for slightly non-centrosymmetric D–π–D quadrupolar chromophores.^18^

**Fig. 5:**
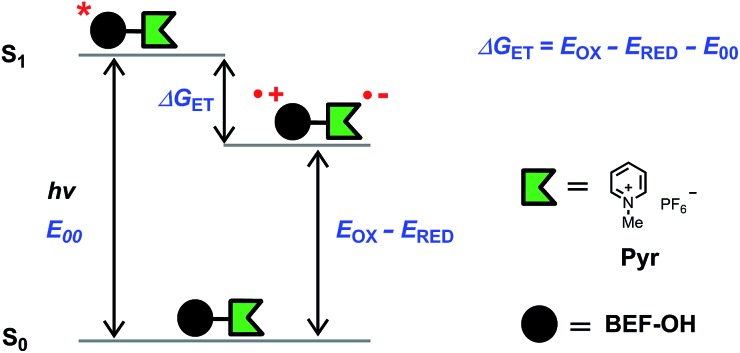
Diagram representing the energy levels of the species involved in electron transfer. Right: structure of *N*-methyl pyridinium hexafluorophosphate (**Pyr**), the model compound used in electrochemical measurements.

The model electron acceptor, *N*-methyl pyridinium hexafluorophosphate (**Pyr**) (Fig. 5), displays weak absorption in the UV region (*λ*_max_ = 270 nm, *ε*_270_ = 0.5 × 10^4^ M^–1^ cm^–1^ in THF, Fig. S26†), with no significant absorption at wavelengths greater than 300 nm. The difference between the absorption spectra of the dye unit **BEF-OH** and release platform **Pyr**, mean that the fluorene dye is the only absorbing species at wavelengths longer than 300 nm.

### Thermodynamics of electron transfer

The fundamental requirement for efficient PeT is that the Gibbs free energy (Δ*G*_ET_) for the process must be negative. The energy of the singlet excited state of the dye (*E*_00_) must be greater than the energy cost of transferring an electron from the donor to the acceptor, *i.e.* greater than the difference between the oxidation potential of the donor (*E*_OX_) and the reduction potential of the acceptor (*E*_RED_), corrected by the Coulombic stabilization of the charges, as summarized by Fig. 5 and eqn (1),^23^

1 Δ*G*_ET_ = *N*_A_{*e*[*E*_OX_ – *E*_RED_] + *w*(D^+^˙A^–^˙) – *w*(DA)} – *E*_00_

where *w*(D^+^˙A^–^˙) and *w*(DA) are terms that factor in electrostatic interaction in the products and reactants:2
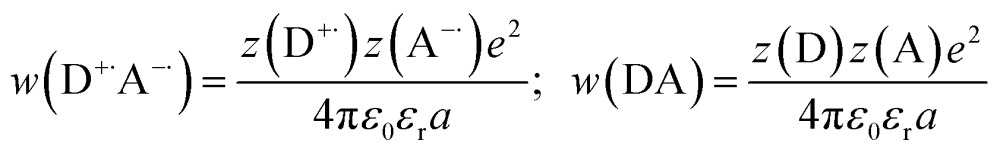
and *N*_A_ is the Avogadro constant, *e* is the elementary charge, *ε*_0_ is the vacuum permittivity, *ε*_r_ is the dielectric constant of the solvent, *a* is the distance of charge separation and *z*(D/A) is charge of the species (D: donor; A: acceptor). The excited state energy (*E*_00_) is defined as the energy of transition between the lowest vibrational level of the ground and excited states, and can be estimated from the point of overlap between the absorption and emission spectra. The oxidation potential of the donor (*E*_OX_) and reduction potential of the acceptor (*E*_RED_) can be measured electrochemically. The excited state energy of **BEF-OH** in THF was estimated at 2.96 eV (418 nm) and the first oxidation potential was determined to be 0.36 V (relative to ferrocene in THF with 0.1 M Bu_4_PF_6_). The reduction potential of **Pyr** relative to ferrocene is –1.76 V under the same conditions. The free Gibbs energy (Δ*G*_ET_) for PeT calculated according to eqn (1) for the pair **BET-OH** and **Pyr** is –0.84 eV indicating that PeT is strongly favorable. The Coulombic term is zero in the case of the pyridinium-based systems because electron transfer does not result information of a charge-separated state but only in the migration of a pre-existing charge. An extended study of photoinduced electron transfer in model dyads, in which the BEF electron donor is covalently linked to a variety of electron acceptors, is reported separately.^24^

### Fluorescence quenching

The efficiency of electron transfer in the systems reported here can be evaluated from their fluorescence quantum yields, because PeT competes directly with fluorescence. Comparison of the fluorescence quantum yields of the free donor unit (**BEF-OH**; *φ* = 0.10 in water) and the donor incorporated into the dyad (**BEF-Pyr-GABA**; *φ* = 0.001 in water) shows that the fluorescence of the fluorene dye is severely quenched by the presence of acceptor, implying that intramolecular PeT is fast and efficient. Using this information and eqn (3), the quantum yield of charge transfer is estimated to be near unity (*φ*_CT_ = 0.98).3
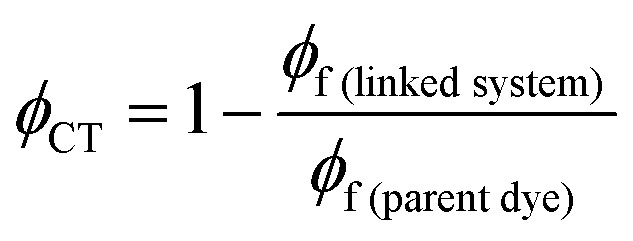



### Hydrolytic stability

**Fig. 6:**
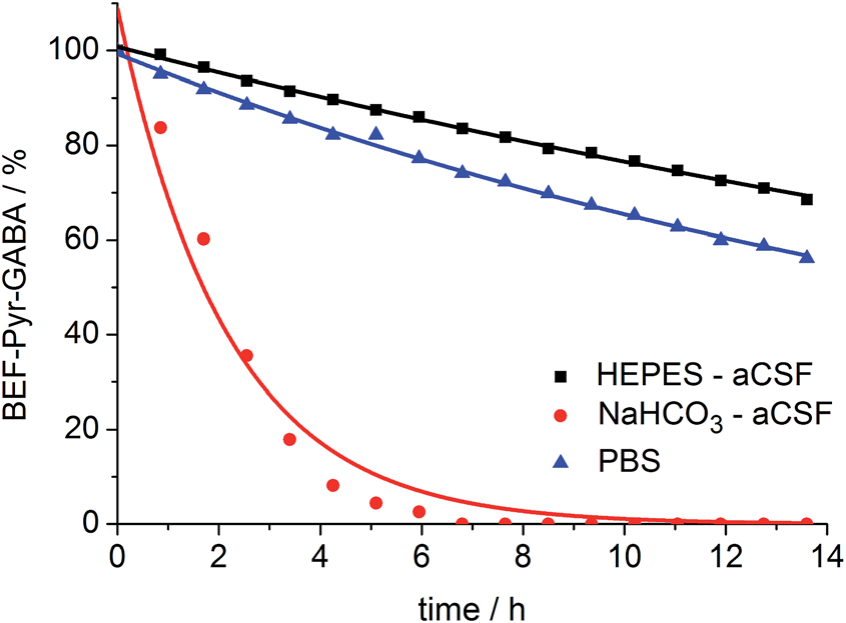
Stability of **BEF-Pyr-GABA** (20 μM) in aqueous buffers at pH 7.4 at 20 °C: HEPES-based aCSF – black, NaHCO_3_ based aCSF – red, PBS – blue; HEPES: 4-(2-hydroxyethyl)piperazine-1-ethanesulfonic acid, aCSF: artificial cerebrospinal fluid, PBS: phosphate-buffered saline. Changes in concentration of **BEF-Pyr-GABA** were determined by HPLC. Mono-exponential fitting curves were applied to the data.

A prerequisite for applications of a caged drug in physiological experiments is that it must be stable in aqueous media, in the absence of light, at least for a few hours. The stability of **BEF-Pyr-GABA** was assessed in aqueous buffers at pH 7.4 by HPLC and in non-buffered D_2_O by NMR at 20 °C. We found that its stability is sensitive to pH and to the composition of the buffer. The half-life of **BEF-Pyr-GABA** in phosphate-buffered saline (PBS) was found to be *ca.* 15 h (Fig. 6; [**BEF-Pyr-GABA**]: 20 μM). In contrast, in NaHCO_3_-based artificial cerebrospinal fluid (aCSF buffer, pH 7.4, which is routinely used as a medium in experiments with neurons) the half-life was reduced to 1.5 h. An extensive set of troubleshooting experiments, in which eight versions of aCSF-buffer were prepared (each missing one different component) allowed us to identify HCO_3_^–^ as the detrimental component. When NaHCO_3_ was replaced with HEPES [4-(2-hydroxyethyl)-1-piperazineethanesulfonic acid, an alternative component of standard buffers used in neuroscience] the half-life of **BEF-Pyr-GABA** rose to *ca.* 50 h. No hydrolysis was detected in non-buffered D_2_O (Fig. S31†). These observations highlight the necessity of evaluating the stability of caged compounds under conditions identical to those of the final target application. We have not investigated how bicarbonate catalyzes this hydrolysis reaction, but the hydrolysis of α-amino acid esters under similar conditions has been attributed to CO_2_-mediated carbamate formation and intramolecular cyclization.^25^ The low hydrolytic stability of GABA-pyridinium esters in standard aCSF buffer poses a limitation for the use of these compounds under strictly physiological conditions that will need to be addressed in future molecular designs.

The hydrolytic stability of **BEF-Pyr-Trp** was monitored only by HPLC and is different from that of **BEF-Pyr-GABA**. **BEF-Pyr-Trp** has a half-life of only about 2 h at pH 7.4 (in both PBS and NaHCO_3_ based aCSF buffers). The hydrolysis proceeds more slowly at pH 3.0 (citric acid/citrate buffer), with a half-life of 3 h (Fig. S36†). The close proximity of the protonated amino group to the ester functionality probably increases the electrophilicity of the carbonyl center, enhancing the hydrolytic instability of the tryptophan derivative. **BEF-Pyr-Trp** also undergoes decomposition at low pH, in a reaction that appears to be associated with the fluorene dye rather than ester hydrolysis (Fig. S37†).

### Uncaging studies

We evaluated the uncaging properties of **BEF-Pyr-Trp** and **BEF-Pyr-GABA** in a series of one-photon irradiation experiments. The method used to monitor release of the amino acid was dictated by properties of caged species. The high aqueous solubility of **BEF-Pyr-GABA** facilitated photolysis at mM concentration and we followed the reaction by ^1^H NMR spectroscopy. It was not possible to monitor the release of GABA by HPLC because this amino acid lacks a UV chromophore. In contrast, **BEF-Pyr-Trp** gave broad ^1^H NMR spectra at mM concentrations, presumably due to aggregation. Photolysis studies were conducted at μM concentration and the indole motif in the side chain of tryptophan enabled quantification of the released amino acid by HPLC.

**Fig. 7:**
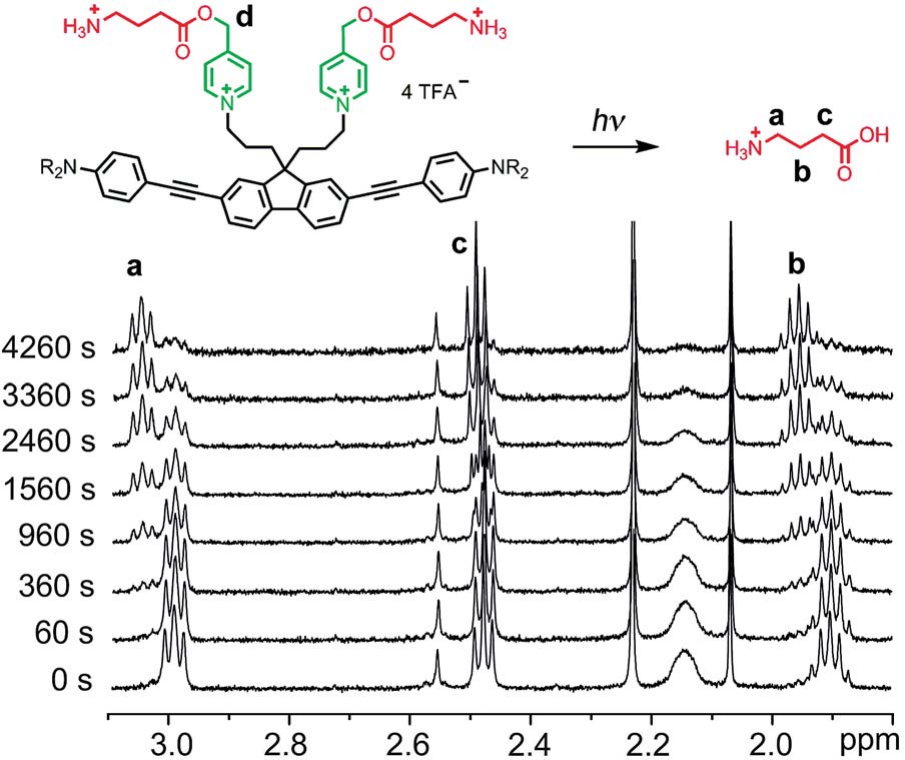
^1^H-NMR spectra from a representative uncaging experiment, which shows the disappearance of caged-GABA signals with a concurrent increase of a new set of multiplets later found to be free GABA (signals “a-c”). Decrease in intensity of signal “d” (5.29 ppm) is not shown.

**Fig. 8:**
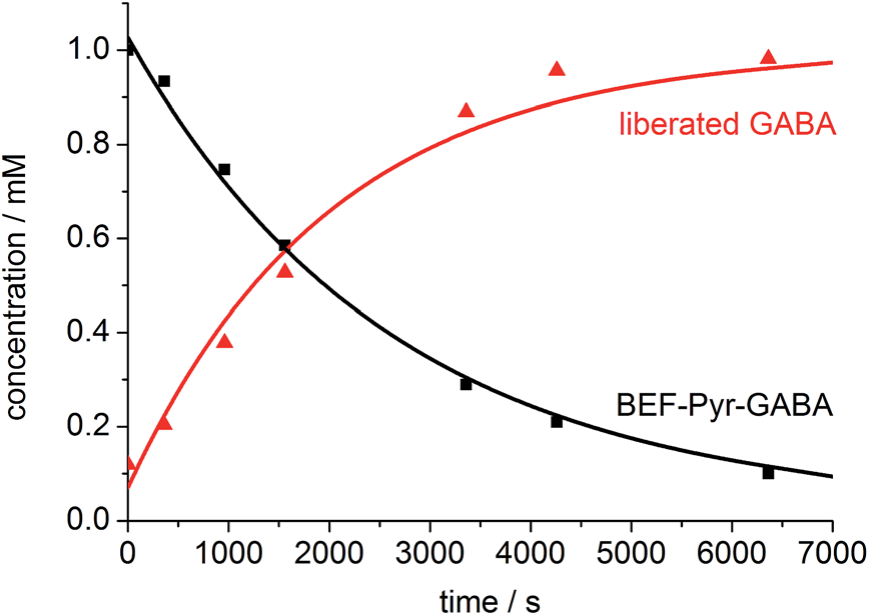
The change in concentration of **BEF-Pyr-GABA** (black squares) and free GABA (red triangles) over time. Concentration of free GABA and **BEF-Pyr-GABA** were determined by integration of signal “c” and “d” respectively relative to *t*-butanol. The presence of free GABA was confirmed by doping an irradiated solution with an authentic sample, which resulted in no new signals and an increase in the intensity of the suspected GABA signals (Fig. S30†).

One-photon uncaging of the pyridinium-based protecting group was initially investigated by irradiating a 1 mM D_2_O solution of **BEF-Pyr-GABA** with a broad UV-A source (300–400 nm, peak 350 nm) in an NMR tube. At this concentration, the transmittance of the solution is negligible across the entire wavelength range of the light source. ^1^H NMR spectroscopy (with *t*-butanol as an internal reference) demonstrated the release of GABA with a chemical yield of >95% (Fig. 7 and 8). Two GABA molecules are released from each molecule of **BEF-Pyr-GABA**, which demonstrates that the BEF chromophore is able to undergo two cycles of photoreduction. We were unable to identify the chemical products generated by photolysis of the caging group.

The photolysis of **BEF-Pyr-Trp** (20 μM in water) with 300–400 nm light (350 nm peak) was monitored by HPLC. Complete consumption of the starting material resulted in release of tryptophan with 83% chemical yield. No photochemically generated byproducts were detected but decomposition of the chromophore unit was observed after the uncaging events.

The quantum yield of uncaging of **BEF-Pyr-GABA** was determined by comparison with the commercially available DPNI-GABA, which has a known *φ*_u_ of 0.085.^26^ Solutions of **BEF-Pyr-GABA** and DPNI-GABA (1 mM, D_2_O) were irradiated simultaneously (300–400 nm, peak 350 nm), and their respective rates of uncaging (*k*_u_) were determined by ^1^H-NMR (see ESI†). Under these concentrated conditions, the rate of uncaging depends only on the light intensity and the quantum yield, but not on the molar absorption coefficient. The uncaging quantum yield of **BEF-Pyr-GABA** was calculated using eqn (4) to give *φ*_u_ = 0.009 ± 0.003.4
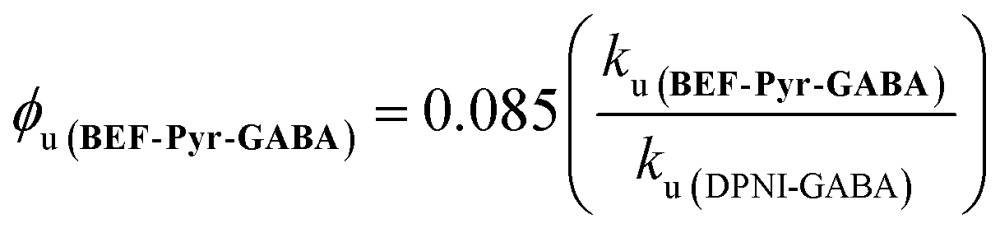



This determination of the uncaging quantum yield of **BEF-Pyr-GABA** was verified using ferric oxalate actinometry,^27^ which gave an uncaging quantum yield of *φ*_u_ = 0.088 ± 0.004 for DPNI-GABA (in close accord with the published value) and an uncaging quantum yield of *φ*_u_ = 0.009 ± 0.004 for **BEF-Pyr-GABA**.

The fluorescence quenching experiments showed that 98% of absorption events lead to charge transfer, while the overall uncaging quantum yield is only about 1%, which suggests that decay of the excited state is dominated by back electron transfer to the ground state. Nevertheless, due to the high value of the TPA cross section (1100 GM at 700 nm), the calculated two-photon uncaging cross section for **BEF-Pyr-GABA** is *δ*_u_ = *δ*_a_ *φ*_u_ = 10 ± 3 GM at 700 nm, which is comparable to the highest reported value (11 GM for the 2-(*o*-nitrophenyl)propyl caged GABA).^11*a*^

**Fig. 9:**
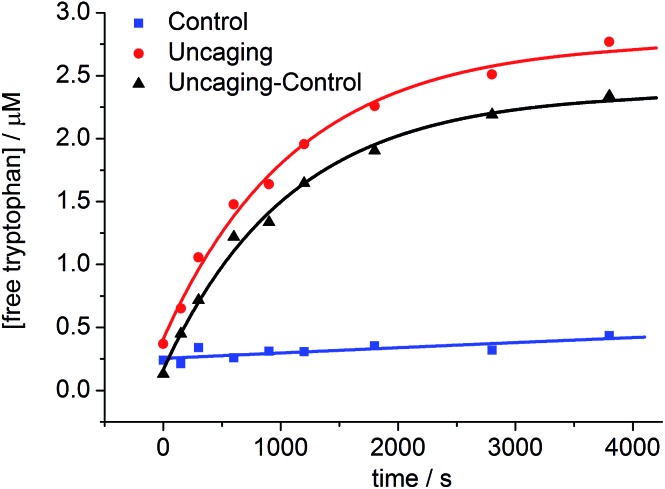
The change in concentration of tryptophan upon photolysis of **BEF-Pyr-Trp** at pH 3.0 and 360 nm; red circles – photolyzed sample, blue squares – control samples stored in the dark, black triangles – net concentration of tryptophan released upon uncaging. The concentration of tryptophan was determined by HPLC.

The uncaging quantum yield for **BEF-Pyr-Trp** was also determined by ferric oxalate actinometry. A solution of **BEF-Pyr-Trp** (∼1.5 μM, pH 3.0) was irradiated at 360 nm with a fluorimeter and photorelease of tryptophan was monitored by HPLC (Fig. 9), using a protocol designed to take account of competing background hydrolysis (see ESI†). The quantum yield of uncaging was measured for **BEF-Pyr-Trp** as *φ*_u_ = 0.0025 (at 360 nm), so the calculated two-photon uncaging cross-section for **BEF-Pyr-Trp** at 720 nm is only *δ*_u_ = 2.5 GM. The reasons for the difference in uncaging quantum yield between **BEF-Pyr-Trp** and **BEF-Pyr-GABA** are unclear and will require further investigation.

**Fig. 10:**
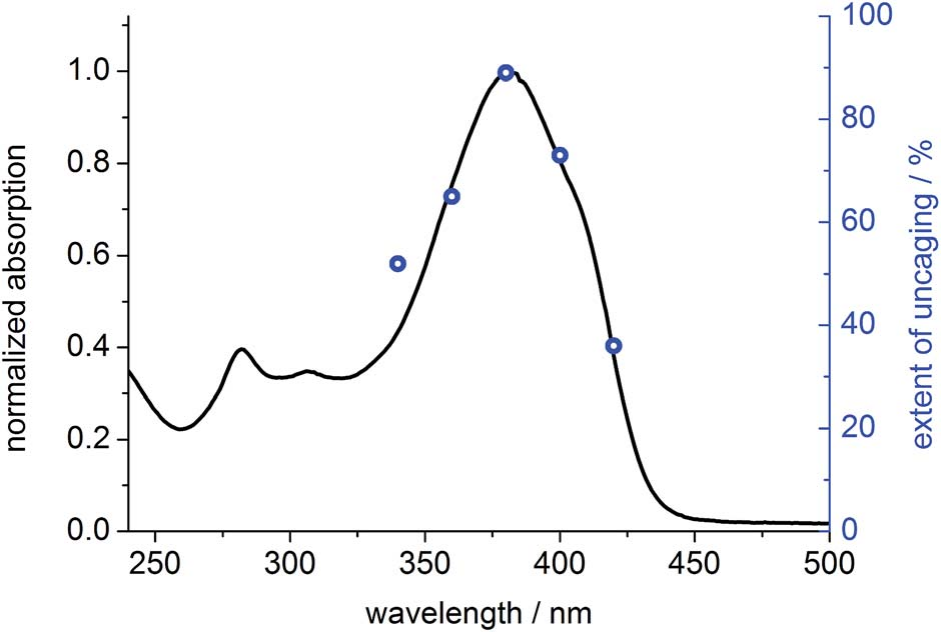
Normalized absorption spectrum of **BEF-Pyr-Trp** in citric acid/sodium citrate buffer (pH 3.0) (black line) plotted in the scale with extent of photolysis, expressed as yield of released tryptophan upon 3800 s of irradiation of **BEF-Pyr-Trp** at 340–420 nm (blue circles).

To prove that photo-cleavage of our new protecting group is sensitized by the absorption of the fluorene-based dye, rather than by direct excitation of pyridinium unit, we investigated the efficiency of uncaging as a function of irradiation wavelength. For this purpose, solutions of **BEF-Pyr-Trp** (1.5 μM, pH 3.0) were irradiated at 340, 360, 380, 400 and 420 nm for 3800 s, and dark control experiments were carried out, to account for background hydrolysis (Fig. 9). The extent of uncaging correlates closely with the absorption spectrum of the BEF chromophore (Fig. 10), confirming the active role of the fluorene-dye in uncaging and PeT mediated release of tryptophan from **BEF-Pyr-Trp**.

Preliminary experiments were carried out to test the two-photon excited release of GABA in the proximity of cultured neurons using **BEF-Pyr-GABA**. Upon two-photon excitation at 720 nm (300 fs, Ti:sapphire laser; 2–5 ms duration), we observed changes in membrane potential with kinetics consistent with activation of GABA-A receptors, indicating GABA release. Further experiments are needed to test the effectiveness of this caged compound, and to quantify side-effects, such as the biological activity of the caged drug.

### Non-pyridinium designs

**Fig. 11:**
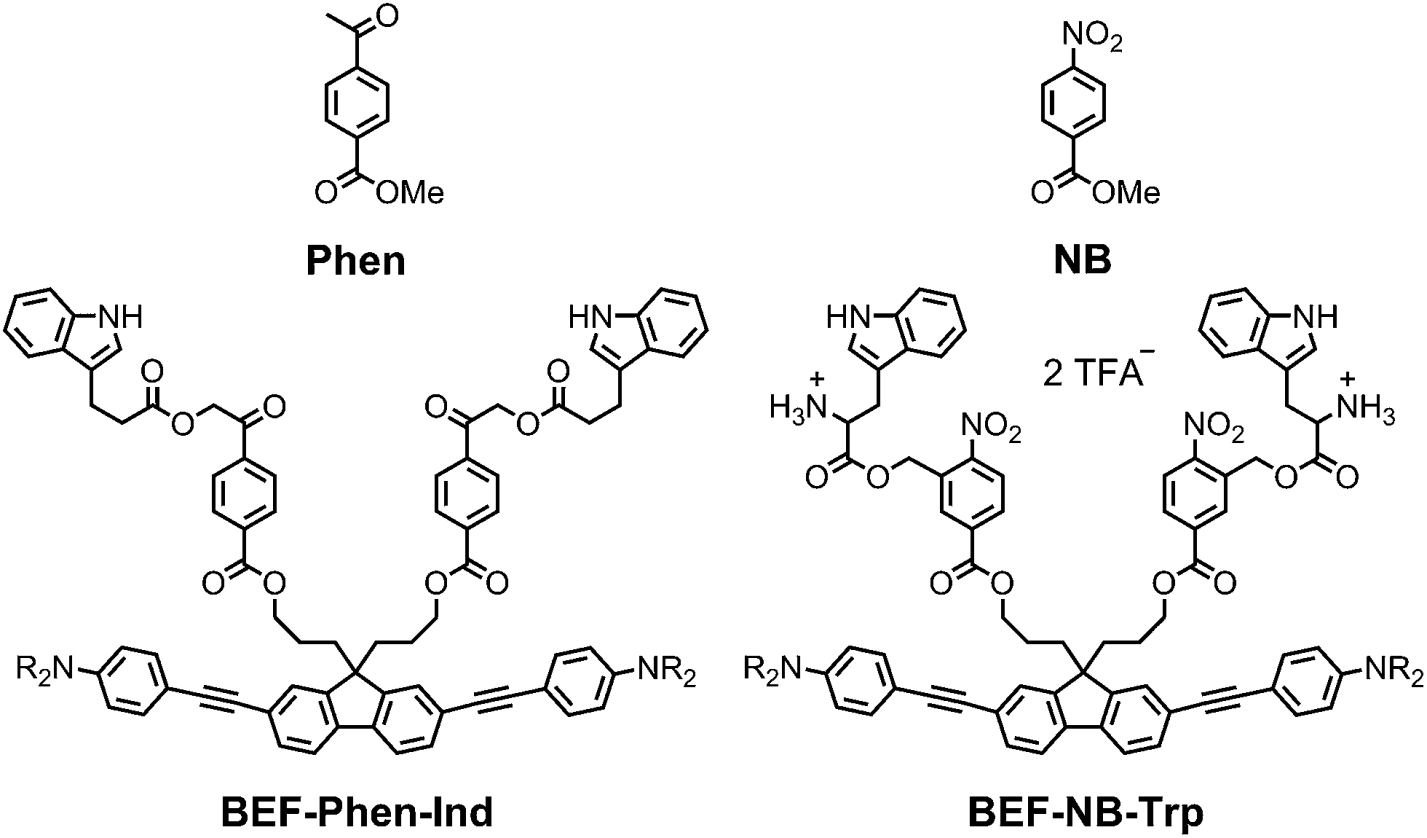
The structures of alternative electron acceptor units: methyl 4-acetylbenzoate (**Phen**) and methyl 4-nitrobenzoate (**NB**) and corresponding protecting groups based on the banana-shaped fluorene dye **BEF-Phen-Ind** and **BEF-NB-Trp**; R = (CH_2_CH_2_O)_7_CH_3_.

The pyridinium group is an excellent electron-acceptor, and the results presented above show that it gives very efficient PeT, however the efficiency of cleavage of the charge-separated state is disappointing (*φ*_u_ ≈ 1%). The pyridinium group may also exhibit undesirable reactivity towards nucleophiles, so we decided to explore other electron acceptors. We chose to investigate phenacyl, for which uncaging *via* PeT has been previously reported^13*b*^ and nitrobenzyl esters (Fig. 11). The reduction potentials of both methyl 4-acetylbenzoate (**Phen**) and methyl 4-nitrobenzoate (**NB**) were measured by cyclic and square wave voltammetry, giving *E*_RED_ of **Phen**: –2.18 V; *E*_RED_ of **NB**: –1.47 V (*vs.* Fc/Fc^+^ in THF with 0.1 M Bu_4_PF_6_). We estimated the values of the Coulombic term, eqn (2), in **BEF-Phen-Ind** and **BEF-NB-Trp** from the distance of photoinduced charge separation (*a*) using molecular mechanics calculations (see ESI†). This gave *w*(D^+^˙A^–^˙) = –0.21 eV for both compounds. Calculation of Gibbs energy of the photoinduced electron transfer (Δ*G*_ET_), according to eqn (1), revealed that it is energetically favorable in both systems, giving Δ*G*_ET_ of –0.54 eV and –1.25 eV for **Phen** and **NB** respectively. To evaluate the photo-release properties of phenacyl and nitrobenzyl derived groups, we synthesized their tryptophan analogues for HPLC-monitored uncaging experiments. In the case of the phenacyl group, attack of the free amino group of tryptophan on the ketone group to form a 6-membered ring posed a limitation for protection of α-amino acids. This issue was overcome by use of the alternative structure: 3-indolepropionic acid (**Ind**), which possesses an indole chromophore but lacks the amino group, allowing us to preserve the absorption properties of tryptophan while avoiding cyclization. Structures of nitrobenzyl tryptophan (**BEF-NB-Trp**) and phenacyl protected 3-indolepropionic acid (**BEF-Phen-Ind**) are shown in Fig. 11 (for synthesis see ESI†).

Photolysis of **BEF-Phen-Ind** was investigated by irradiating a solution in water (17 μM) with a broad UV-A light source (300–400 nm; peak: 350 nm), monitoring the progress of uncaging by HPLC. 3-Indolepropionic acid was liberated with a modest 20% chemical yield, despite full consumption of the starting material. HPLC analysis did not reveal formation of any other products of photolysis and we were unable to define the fate of the remaining 80% of starting material. The quantum yield of uncaging was determined by use of ferric oxalate actinometry, giving *φ*_u_ = 0.0022, corresponding to a two-photon uncaging cross section of *δ*_u_ = 2.4 GM at 700 nm. The limited scope of substrates that can be caged (due to the reactivity of the ketone group), the low chemical yield of photorelease and the susceptibility to hydrolysis under physiological conditions make ester-linked phenacyl platforms unattractive release units.

Photolysis of **BEF-NB-Trp** was tested in a range of solvents (5 μM concentration in water, NaHCO_3_-aCSF, acetonitrile, methanol and THF) using a broad UV-A light source (300–400 nm; peak: 350 nm), but no release of tryptophan was observed. Photochemical decomposition of **BEF-NB-Trp** occurred, but it did not result information of free tryptophan. The same result was observed when irradiation was carried out at 280 nm. Tryptophan was liberated cleanly in 75% yield by hydrolysis of **BEF-NB-Trp** in the dark over 40 h in NaHCO_3_-based aCSF buffer.

## Conclusions

In this study, we have investigated an approach to the rational design of two-photon photo-labile protecting groups. A protecting group has been developed which operates by PeT between an electron-rich fluorene-based dye and a pyridinium electron-acceptor. The fluorescence of the dye is quenched by electron transfer to the pyridinium; charge-transfer leads to bond scission, to liberate a carboxylic acid. Our protecting group has been demonstrated to release the neurotransmitter GABA and amino acid l-tryptophan upon irradiation with light of wavelength 340–420 nm, in aqueous solution in nearly quantitative chemical yields. This group exhibits a high TPA cross-section (1100 GM at 700 nm) and highly efficient charge-transfer between the electron donor and acceptor was observed (98%). The fast back electron transfer from the charge-shifted state reduces the overall quantum efficiency of uncaging to around 1% which, when combined with the TPA cross-section, results in a two-photon uncaging cross-section of approximately *δ*_u_ = 10 GM (700 nm) for **BEF-Pyr-GABA**. Wavelength-dependent uncaging experiments confirmed electron-transfer mediated release of caged tryptophan with efficiency of release proportional to the extinction coefficient of the fluorene dye within 340–420 nm.

A key objective for future research will be to apply the modular design strategy demonstrated in this study to create an electron donor–acceptor pair for which the back electron transfer is suppressed, so that bond-scission becomes the main decay pathway. The susceptibility of pyridinium esters towards hydrolysis can lead to practical difficulties for uncaging studies in aqueous media, and it would be useful to extend these systems to non-ester linking unit that is are more stable to aqueous hydrolysis, such as carbamates.
