# Supplementary material for: Two-photon sensitive protecting groups operating via intramolecular electron transfer: uncaging of GABA and tryptophan
Source: Chem Sci. 2015 Feb 3;6(4):2419–26. doi: 10.1039/c4sc03775h (PMC5488212; doi:10.1039/c4sc03775h)
Supplement: Supplementary file 1 [file SC-006-C4SC03775H-s001.pdf]

Electronic Supplementary Material (ESI) for Chemical Science.
This journal is © The Royal Society of Chemistry 2015

Supplementary Electronic Information

Two-photon sensitive protecting groups operating *via* intramolecular electron transfer: uncaging of GABA and tryptophan

Karolina A. Korzycka,[‡a] Philip M. Bennett,[‡a] Eduardo Jose Cueto-Diaz[b], Geoffrey Wicks,[c] Mikhail Drobizhev,[c] Mireille Blanchard-Desce,[b] Aleksander Rebane[cd] and Harry L. Anderson[a*]

[a] Oxford University, Department of Chemistry, Chemistry Research Laboratory, 12 Mansfield Road, Oxford, OX1 3TA, UK. E-mail: harry.anderson@chem.ox.ac.uk; Fax: +44 (0)1865 285002; Tel: +44 (0)1865 275704

[b] Université de Bordeaux, Institut des Sciences Moléculaires, CNRS UMR 5255, 33400 Bordeaux, France.

[c] Department of Physics, Montana State University, Bozeman, MT 59717, USA

[d] National Institute of Chemical Physics and Biophysics, Tallinn 12618, Estonia

[‡] K.A.K. and P.M.B. made equal contribution to this work.

Table of Contents

1. Synthesis **2**

1.1 General methods 2

1.2 Synthetic procedures 3

1.2.1. Molecular design 3

1.2.2 Pyridinium deriviatives 7

1.2.3 Phenacyl group 13

1.2.4 *o*-Nitrobenzyl group 16

2. NMR and MS spectra, HPLC chromatograms **19**

3. Evaluation of physical and photophysical properties of model compounds **37**

3.1 Electrochemical measurements 37

3.2.Absorption and emission spectra of model compounds 40

4. One-photon photolysis **42**

4.1 One-photon uncaging of **BEF-Pyr-GABA** 42

4.2 The determination of the quantum yield of uncaging 45

4.2.1 Comparison with the **DPNI-GABA** as a standard 45

4.2.2 Actinometry 46

4.3 One-photon photolysis of **BEF-Pyr-Trp**. 47

4.3.1 Wavelength dependent uncaging experiments. 47

4.3.2 Quantum yield of uncaging of **BEF-Pyr-Trp** 50

5. Stability experiments **51**

7. Thermodynamics of electron transfer in non-pyridinium design **52**

8. Quantum yield of uncaging of BEF-Phen-Ind **53**

9. Literature references **54**

1. Synthesis

1.1 General methods

All solvents and reagents were purchased from commercial suppliers (Fisher or Sigma-Aldrich). DPNI-GABA was purchased from Tocris Biosciences. Anhydrous THF, DCM and MeCN were obtained by passing solvents through a column of activated alumina. Diisopropylamine (DIPA) and triethylamine (Et_3N) were distilled from CaH_2. All other reagents were used as supplied by commercial agents. Analytical thin layer chromatography *TLC* was carried out on Merck silica gel 60 F_{254} aluminum supported plates and visualized by absorption of UV light. Flash column chromatography was performed with VWR silica gel 60 applying pressure of N_2. Size exclusion chromatography was carried out with use of Bio-rad Bio-beads S-X1. HPLC separation was conducted on an Agilent 1100 system equipped with a G1315B diode array detector, a G1311A quaternary pump and a G1316A fraction collector. Analytical HPLC was performed with C18 5 µm, 4.6 × 150 mm Eclipse XDB-C18 column (Agilent) and C18 5 µm, 2.1 × 50 mm Zorbax SB-C18 column (Agilent) using 1 mL min^{-1} flow and stepwise gradient at 40 °C. Semi-preparative purification was carried out with use of C8 5 µm, 9.4 × 250 mm Eclipse XDB-C8 column (Agilent) and 3 mL min^{-1} solvent flow. The chromatographic separations were monitored in the range 190–900 nm.

HPLC method 1:

Time [min]	H_2O (0.1% TFA) [v/v, %]	MeCN [v/v, %]
0.00	95	5
9.00	0	100
11.00	0	100

HPLC method 2:

Time [min]	H_2O (0.1% TFA) [v/v, %]	MeCN [v/v, %]
0.00	95	5
9.00	0	100
18.00	0	100

HPLC method 3:

Time [min]	H_2O (0.1% TFA) [v/v, %]	MeCN [v/v, %]
0.00	100	0
20	0	100

ESI measurements were performed operating in positive or negative mode on a Waters LCT Premier (LRMS) or Bruker µTOF (HRMS) from acetonitrile solutions. MALDI-ToF mass spectrometry was carried out using a Micromass MALDI micro MX spectrometer and following matrices: dithranol (1,8,9-anthracenetriol), DTCB (*trans*-2-[3-(4-*tert*-butylphenyl)-2-methyl-2-propenylidene]malononitrile), CHCA (α-cyano-4-hydroxycinnamic acid). ESI spectra in Figure S**14** and S**22** were measured at the EPSRC National Mass Spectrometry service (Swansea) using the positive nano-electrospray on the LTQ Orbitrap XL.

NMR spectra were acquired at ambient temperature with Bruker instruments DPX200 (200 MHz), DPX250 (250 MHz), DPX400 (400 MHz), AV400 (400 MHz), DRX500 (500 MHz). Chemical shifts for the ^{1}H-NMR and ^{13}C spectra are reported with $CDCl_3$ or DMSO-d_6 as reference. Data are displayed as follows: chemical shift δ (ppm), multiplicity, integration and coupling constants *J* (Hz).

1.2 Synthetic procedures

1.2.1. Molecular design

2,5,8,11,14,17-Hexaoxanonadecan-19-yl 4-methylbenzenesulfonate

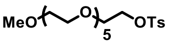

This compound was prepared by analogy with a reported procedure.[S1] To a cooled suspension of TsCl (4.1 g, 21.9 mmol) in DCM (5 mL) at 0 °C was added a solution of hexaethylene glycol monomethyl ether (5.0 g, 16.8 mmol) in DCM (5 mL) and Et_3N (4.7 mL, 33.6 mmol), under inert atmosphere. Reaction mixture was allowed to warm to 20 °C and was stirred for 16 h. After this time, it was poured into H_2O (20 mL) and extracted with EtOAc (50 mL), Et_2O (50 mL) and $CHCl_3$ (50 mL). The organic washings were combined, dried over $MgSO_4$ and evaporated to give 7.04 g (15.6 mmol, 93% yield) of compound as a yellow oil.

^{1}H NMR (400 MHz, $CDCl_3$): *δ* 2.20 (s, 3H), 3.10 (s, 3H), 3.26–3.43 (m, 22H), 3.88–3.91 (m, 2H), 7.12 (d, *J*=8.1 Hz, 2H), 7.53 (d, *J*=8.1 Hz, 2H); ^{13}C NMR (62 MHz, $CDCl_3$): *δ* 21.7, 59.0, 68.7, 69.7, 70.6–70.7, 72.0, 128.1, 130.2, 133.3, 145.0; *m/z* ESI+ 473.2, $[M+Na]^+$, $C_{20}H_{34}NaO_9S^+$ requires 473.18 (100%).

2,2'-((4-Iodophenyl)azanediyl)bis(ethan-1-ol) (1)

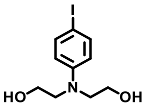

This compound was prepared according to the literature procedure.[S2] Iodine (10.4 g, 41.4 mmol) was added to a cooled solution of *N,N'*-phenyldiethanoloamine (2.5 g, 13.8 mmol) in dioxane and pyridine (1:1, 30 mL) at 0 °C . After 1 h of stirring at 0 °C the reaction mixture was allowed to warm to 20 °C and stirred for next 16 h. After this time, a saturated solution of $Na_2S_2O_3$ (15 mL) was added and the mixture was washed with EtOAc (20 mL). The organic

phase was dried over $MgSO_4$ and evaporated. The crude product was dissolved in DCM and precipitated with hexane to give 3.47 g (11.3 mmol, 81% yield) of compound **1**.

^{1}H NMR (200 MHz, $CDCl_3$): δ 3.37–3.44 (m, 4H), 3.52–3.58 (m, 4H), 6.55 (d, *J*=9.1 Hz, 2H), 7.39 (d, *J*=9.1 Hz, 2H); ^{13}C NMR (50 MHz, $CDCl_3$): δ 53.7, 58.5, 75.8, 114.3, 137.6, 148.0; *m/z* ESI+ 330.0, $[M+Na]^+$, $C_{10}H_{14}INNaO_2^+$ requires 329.9 (100%).

2,2'-((4-((Triisopropylsilyl)ethynyl)phenyl)azanediyl)bis(ethan-1-ol) (2)

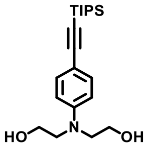

This compound was synthesized by adapting a reported procedure.[S3] 2,2'-((4-Iodophenyl)azanediyl)bis(ethan-1-ol) (**1**) (0.80 g, 2.6 mmol), $Pd(OAc)_2$ (116 mg, 0.50 mmol), PPh_3 (270 mg, 1.0 mmol) and CuI (65 mg, 0.30 mmol) were placed in a Schlenk tube and the system was flushed with nitrogen. Distilled DIPA (12 mL) was added and reaction mixture was degassed by two freeze-thaw cycles. Ethynyltriisopropylsilane (1.6 mL, 7 mmol) was added and additional freeze-thaw cycle was run. Reaction was left stirring at 50 °C for 12 h. After this time, the reaction mixture was allowed to cool to 20 °C and EtOAc (20 mL) was added. The organic phase was washed with H_2O (20 mL), saturated solution of NH_4Cl (20 mL) and H_2O (20 mL). Organic layer was dried over $MgSO_4$ and evaporated. The product was isolated by column chromatography on silica ($CHCl_3$ - MeOH in gradient 0–4% of MeOH) to give 740 mg (2.0 mmol, 78%) of compound **2**.

^{1}H NMR (400 MHz, $CDCl_3$): δ 1.27 (m, 21H), 3.54–3.57 (m, 4H), 3.78–3.82 (m, 4H), 3.99 (bs, 2H), 6.56 (d, *J*=8.5 Hz, 2H), 7.34 (d, *J*=8.5 Hz, 2H); ^{13}C NMR (100 MHz, CDCl3): δ 11.4, 18.7, 55.2, 60.6, 87.8, 107.9, 111.1, 111.9, 133.3, 147.5; *m/z* EI+ 361.24, $[M]^+$, $C_{21}H_{35}NO_2Si^+$ requires 361.24 (100%).

***N,N*-Di-(2,5,8,11,14,17,20-heptaoxadocosan-22-yl)-*N*-(4-((triisopropylsilyl)ethynyl) phenyl)-amine (3)**

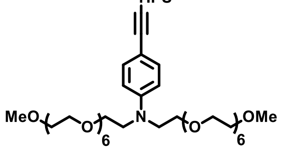

This novel compound was prepared by analogy with a reported procedure.[S4] 2,2'-((4-((triisopropylsilyl)ethynyl)phenyl)azanediyl)bis(ethan-1-ol) (**2**) (1.25 g, 3.5 mmol) and NaH (0.63 g of 60% dispersion in mineral oil, 15.9 mmol) were suspended in THF (5 mL) and the reaction mixture was refluxed for 1 h under inert atmosphere followed by the addition of 2,5,8,11,14,17-hexaoxanonadecan-19-yl 4-methylbenzenesulfonate (4.70 g, 10.4 mmol) in THF (5 mL). After 48 h of refluxing, the mixture was allowed to cool to 20 °C and H_2O (10 mL) was added. Aqueous phase was extracted with DCM (50 mL); the organic layer was

dried over $MgSO_4$ and evaporated. The product was isolated from the reaction mixture by column chromatography on silica (EtOAc – MeOH, in gradient 0–10% of MeOH) to give 1.90 g (2.07 mmol, 60%) of compound **3**.

^{1}H NMR (200 MHz, $CDCl_3$): δ 1.07 (m, 21H), 3.34 (s, 6H), 3.51–3.63 (m, 56H), 6.59 (d, *J*=9.3 Hz, 2H), 7.27 (d, *J*=9.3 Hz, 2H); ^{13}C NMR (100 MHz, $CDCl_3$): δ 11.4, 18.7, 50.9, 59.0, 68.4, 70.5, 70.5–70.6, 70.7, 71.9, 87.3, 108.3, 110.4, 111.3, 133.3, 147.6; *m/z* ESI+ 940.5, $[M+Na]^+$, $C_{47}H_{87}NNaO_{14}Si^+$ requires 940.6 (100%).

_N,N_-Di-(2,5,8,11,14,17,20-heptaoxadocosan-22-yl)-_N_-(4-ethynylphenyl)-amine (4)

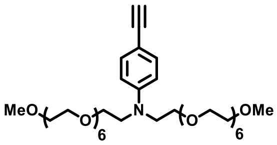

To a solution of *N,N*-di-(2,5,8,11,14,17,20-heptaoxadocosan-22-yl)-*N*-(4-((triisopropylsilyl) ethynyl)phenyl)-amine (**3**) (2.2 g, 2.3 mmol) in THF (2 mL) a solution of TBAF (1.0 M in THF, 11.0 mL, 11.0 mmol) was added and the solution was stirred for 12 h at 20 °C. After this time, the solvent was evaporated and the compound was isolated from the mixture by column chromatography on silica (EtOAc – MeOH, in gradient 0–10% of MeOH) to give 1.5 g (1.9 mmol, 85%) of **4**.

^{1}H NMR (500 MHz, $CDCl_3$):δ 2.90 (s, 1H), 3.25 (s, 6H), 3.42–3.53 (m, 56H), 6.50 (d, *J*=8.0 Hz, 2H), 7.17 (d, *J*=8.0 Hz, 2H); ^{13}C NMR (125 MHz, $CDCl_3$): δ 50.8, 58.8, 68.3, 70.4, 70.4–70.5, 70.6, 71.8, 75.0, 84.6, 108.4, 111.2, 133.2, 147.9; *m/z* ESI+ 784.3, $[M+Na]^+$, $C_{38}H_{67}NNaO_{14}^+$ requires 784.4 (100%)

3,3'-(2,7-Diiodo-9H-fluorene-9,9-diyl)bis(propan-1-ol) (5)

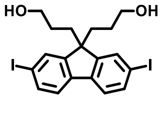

This compound was prepared by analogy with a reported procedure.[S5] A solution of borane-THF complex (1.0 M, 56 mL, 56 mmol) was added slowly to a cooled to 0 °C solution of 3,3'-(2,7-diiodo-9H-fluorene-9,9-diyl)dipropionic acid[S6] (7.2 g, 12.8 mmol) in anhydrous THF (10 mL). After 2 h of stirring at 20 °C reaction mixture was poured into ice (500 mL) and precipitated solid was removed upon filtration. The solid was dissolved in THF and precipitated with Et_2O to give 6.00 g (11.2 mmol, 87% yield) of compound **5** as a white powder.

^{1}H NMR (400 MHz, DMSO-*d*$_6$): δ 0.59–0.65 (m, 4H), 1.96–2.00 (m, 4H), 3.10–3.14 (m, 4H), 7.66 (d, *J*=7.8 Hz, 2H), 7.71 (dd, *J*=1.3 Hz, *J*=7.8 Hz, 2H), 7.83 (d, *J*=1.3 Hz, 2H); ^{13}C NMR (50 MHz, DMSO-*d*$_6$): δ 27.7, 35.7, 55.2, 61.2, 94.4, 122.6, 132.1, 136.3, 139.7, 152.4; *m/z* ESI+ 557.0, $[M+Na]^+$, $C_{19}H_{20}I_2NaO_2^+$ requires 556.9 (100%).

BEF-OH

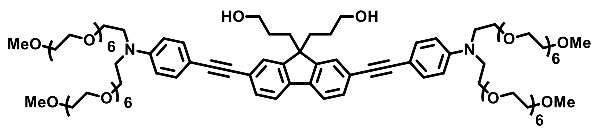

This compound was synthesized by adapting a reported procedure.[S7] The following solids were dried under vacuum: CuI (1 mg, 0.005 mmol), Pd(OAc)$_2$ (1 mg, 0.004 mmol) PPh$_3$ (2 mg, 0.008 mmol) and 3,3'-(2,7-diiodo-9H-fluorene-9,9-diyl)bis(propan-1-ol) (**5**) (50 mg, 0.1 mmol). Distilled DIPA (0.5 mL) was added and two freeze-thaw cycles were carried out. A solution of *N,N*-di-(2,5,8,11,14,17,20-heptaoxadocosan-22-yl)-*N*-(4-ethynylphenyl)-amine (**4**) (157 mg, 0.21 mmol) in anhydrous MeCN (0.5 mL) was added and an additional freeze-thaw cycle was run. The progress of the reaction was monitored by HPLC (*method 1*). After 2 h of stirring at 20 °C the reaction mixture was diluted with DCM (50 mL) and the crude mixture was washed with H_2O (20 mL) and saturated aqueous solution of NH_4Cl (20 mL). The organic layer was dried over $MgSO_4$ and evaporated to dryness. The final compound was isolated from the mixture with use of reverse phase column chromatography (in gradient from 100% of H_2O (0.1% TFA v/v) to 100% MeCN). **BEF-OH** was obtained in 33% yield (55 mg, 0.03 mmol).

^{1}H NMR (400 MHz, CDCl$_3$) δ: 0.87–0.94 (m, 4H), 2.09–2.13 (m, 4H), 3.38 (m, 16H), 3.53–3.55 (m, 8H), 3.60–3.67 (m, 104H), 6.70 (d, *J*=8.5 Hz, 4H), 7.39 (d, *J*=8.5 Hz, 4H), 7.47–7.49 (m, 4H), 7.63 (d, *J*=8.2 Hz, 2H); *m/z* MALDI-TOF$^+$ 1825.08 [M+Na]$^+$, $C_{95}H_{152}N_2O_{30}Na^+$ requires 1825.04 (100%).

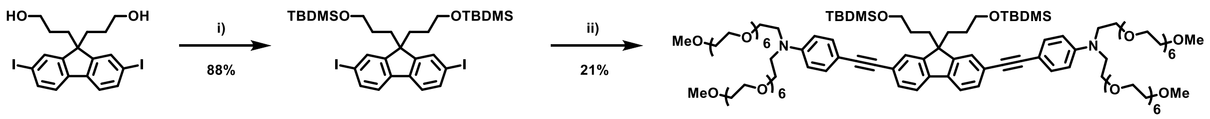

5 21 BEF-OTBDMS

Scheme S1. Reagents: *i)* TBDMSCl, imidazole, DMF, 20 °C, 4 h, *ii)* **4**, Pd(OAc)$_2$, PPh$_3$, CuI, DIPA, 50 °C, 2 h.

(((2,7-diiodo-9H-fluorene-9,9-diyl)bis(propane-3,1-diyl))bis(oxy))bis(*tert*-butyldimethylsilane) (21)

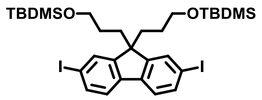

This compound was prepared by analogy with a reported procedure.[S8] To a solution of 3,3'-(2,7-diiodo-9H-fluorene-9,9-diyl)bis(propan-1-ol) (**5**) (100 mg, 0.18 mmol) in DMF (4 mL) TBDMSCl (73 mg, 0.46 mmol) and imidazole (32 mg, 0.47 mmol) were added and the mixture was stirred at room temperature for 3.5 h. After that time, the solvent was evaporated and the product was isolated from the crude reaction mixture by use of column chromatography (PE – DCM; 1:1, v/v) to give 125 mg (0.16 mmol, 88 %) of white solid.

^{1}H NMR (400 MHz, $CDCl_3$): δ 0.01 (s, 12H), 0.77–0.81 (m, 4H), 0.85 (s, 18H), 1.99–2.04 (m, 4H), 3.34 (t, *J*=6.3 Hz, 4H), 7.41 (d, *J*=8.4 Hz, 2H), 7.64–7.67 (m, 4H); ^{13}C NMR (100 MHz, $CDCl_3$): δ -5.0, 18.5, 26.0, 27.2, 36.2, 55.2, 62.9, 93.2, 121.7, 132.2, 136.4, 139.7. 152.2; *m/z* ESI+ 763.4, $[M]^+$, $C_{31}H_{49}I_2O_2Si_2^+$ requires 763.1 (100 %).

BEF-OTBDMS

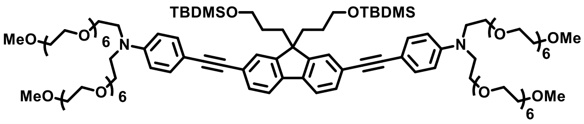

This compound was synthesized by adapting a reported procedure.[S7] To the predried under vacuum CuI (5 mg, 2.6 µmol), $Pd(OAc)_2$ (6 mg, 2.6 µmol), PPh_3 (13 mg, 5.0 µmol) and (((2,7-diiodo-9H-fluorene-9,9-diyl)bis(propane-3,1-diyl))bis(oxy))bis(*tert*-butyldimethylsilane) (**21**) (50 mg, 65 µmol), distilled DIPA (3.0 mL) was added and two freeze-thaw cycles were carried out. A suspension of acetylene **4** (110 mg, 0.14 mmol) in DIPA (1 mL) was added and additional freeze-thaw cycle was run. After 2 h of stirring at 50 °C reaction was diluted with DCM (50 mL) and crude mixture was washed with H_2O (20 mL) and NH_4Cl (20 mL). Organic layer was dried over $MgSO_4$ and evaporated to dryness. The final compound was isolated from the mixture with use of column chromatography on silica (EtOAc – MeOH, in gradient 0-25 % of MeOH). Compound was purified further by size exclusion chromatography (DCM as eluent) and 30 mg (15 µmol, 21 %) of yellow film were obtained.

^{1}H NMR (400 MHz, $CDCl_3$): δ 0.0 (s, 12H), 0.89–0.91 (m, 22H), 2.11–2.15 (m, 4H), 3.40 (t, *J*=6.4 Hz, 4H), 3.44 (s, 12 H), 3.58–3.62 (m, 8H), 3.64-3.74 (m, 104H), 6.74 (d, *J*=8.9 Hz, 4H), 7.44 (d, *J*=8.9 Hz, 4H), 7.50–7.53 (m, 4H), 7.67 (d, *J*=8.4 Hz, 2H); ^{13}C NMR (100 MHz, $CDCl_3$) δ: -5.3, 18.3, 26.0, 27.2, 36.4, 50.8, 54.4, 59.0, 63.2, 68.4, 70.5, 70.5, 70.6-70.7, 71.9, 88.3, 90.8, 110.0, 111.4, 119.7, 122.8, 125.7, 130.5, 132.9, 140.1, 147.6, 150.3; *m/z* MALDI-TOF^+ 2052.15, $[M+Na]^+$, $C_{107}H_{180}N_2O_{30}Si_2Na^+$ requires 2053.21 (100 %).

1.2.2 Pyridinium deriviatives

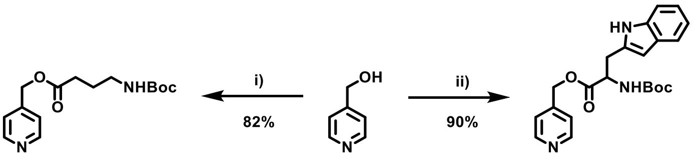

7 10

Scheme S2. Reagents: *i)* Boc-GABA-OH, EDC, DMAP, THF, MeCN, 20 °C, 16 h, *ii)* Boc-Trp-OH, EDC, DMAP, MeCN, 20 °C, 16 h.

Pyridin-4-ylmethyl (*tert*-butoxycarbonyl)tryptophanate (10)

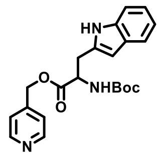

This compound was synthesized by adapting a reported procedure.[S9] 4-Pyridine methanol (0.50 g, 4.6 mmol), EDC (1.07 g, 5.6 mmol), DMAP (56 mg, 0.46 mmol) and Boc-Trp-OH (2.1 g, 6.9 mmol) were dissolved in anhydrous MeCN (5 mL) and mixture was stirred for 8 h. After this time, the solvent was evaporated, the mixture was dissolved in DCM (30 mL) and washed with H_2O (30 mL). The organic phase was dried over $MgSO_4$ and the solvent was evaporated to dryness. The product was isolated by column chromatography on silica (EtOAc as eluent) to yield 1.65 g (4.17 mmol, 90% yield) of compound **10** as a white solid.

^{1}H NMR (400 MHz, $CDCl_3$) *δ*: 1.44 (s, 9H), 3.28 (dd, *J*=6.3 Hz, *J*=14.5 Hz, 1H), 3.35 (dd, *J*=5.4 Hz, *J*=14.5 Hz, 1H), 4.72–4.77 (m, 1H), 5.02 (d, *J*=13.7 Hz, 1H), 5.08 (d, *J*=13.7 Hz, 1H), 5.14 (d, *J*=7.9 Hz, 1H), 6.93 (s, 1H), 7.00 (d, *J*=5.0 Hz, 2H), 7.09–7.13 (m, 1H), 7.18–7.22 (m, 1H), 7.33 (d, *J*=8.1 Hz, 1H), 7.56 (*d*, J=8.1 Hz, 1H), 8.35 (s, 1H), 8.51 (bs, 2H); ^{13}C NMR (100 MHz, $CDCl_3$) δ: 28.1, 28.4, 54.8, 64.8, 80.3, 109.3, 111.7, 118.6, 119.5, 122.0, 123.4, 127.5, 136.5, 145.1, 149.1, 155.6, 172.4; *m/z* ESI-MS+ 396.2, $[M+H]^+$, $C_{22}H_{26}N_3O_4^+$ requires 396.2 (100%).

Pyridin-4-ylmethyl 4-((*tert*-butoxycarbonyl)amino)butanoate (7)

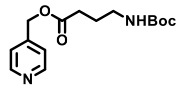

This compound was synthesized by adapting a reported procedure.[S9] 4-Pyridine methanol (130 mg, 1.19 mmol), Boc-GABA-OH (300 mg, 1.47 mmol), EDC (280 mg, 1.47 mmol) and DMAP (14 mg, 0.11 mmol) were dissolved in anhydrous THF and MeCN (4.0 mL, 1: 1, v/v). The mixture was stirred at 20 °C for 16 h. After this time, the mixture was diluted with H_2O (15 mL) and extracted with DCM (3 × 25 mL). The organic extracts were combined, dried over $MgSO_4$ and evaporated to dryness. The product was isolated by column chromatography on silica (eluent: $CHCl_3$) to yield 290 mg (0.98 mmol, 82% yield) of compound **7** as a white solid.

^{1}H NMR (400 MHz, $CDCl_3$): *δ* 1.41 (s, 9H), 1.84 (quin, *J*=7.2 Hz, 2H), 2.45 (t, *J*=7.2 Hz, 2H), 3.14-3.19 (m, 2H), 4.74 (bs, 1H), 5.11 (s, 2H), 7.23 (d, *J*=5.9 Hz, 2H), 8.58 (d, *J*=5.9 Hz, 2H); ^{13}C NMR (100 MHz,$CDCl_3$): *δ* 25.3, 28.4, 31.3, 39.7, 64.2, 79.2, 121.8, 144.8, 150.0, 156.0, 172.7; *m/z* ESI-MS+ 295.1, $[M+H]^+$, $C_{15}H_{23}N_2O_4^+$ requires 295.1 (100%).

(2,7-Diiodo-9H-fluorene-9,9-diyl)bis(propane-3,1-diyl dimethanesulfonate) (6)

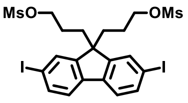

This novel compound was prepared using reaction conditions reported in the literature.[S10] Methanesulfonyl chloride (580 μL, 7.50 mmol) was added dropwise to a cooled (0 °C) solution of 3,3'-(2,7-diiodo-9H-fluorene-9,9-diyl)bis(propan-1-ol) (**5**) (1.00 g, 1.87 mmol) in anhydrous DCM (10 mL), Et_3N (1.6 mL, 11.48 mmol). After 1 h of stirring at 20 °C the mixture was diluted with H_2O and extracted with $CHCl_3$ (50 mL). The organic extracts were combined, dried over $MgSO_4$ and evaporated to dryness. The product was isolated by column chromatography on silica with $CHCl_3$ as eluent to give 900 mg (1.3 mmol, 70% yield) of compound **6** as a white solid.

^{1}H NMR (400 MHz, $CDCl_3$) *δ*: 0.94–1.00 (m, 4H), 2.04–2.09 (m, 4H), 2.89 (s, 6H), 3.89–3.93 (m, 4H), 7.42 (d, *J*=8.5 Hz, 2H), 7.64–7.67 (m, 4H); ^{13}C NMR (100 MHz, $CDCl_3$) *δ*: 23.6, 35.5, 37.4, 54.3, 69.8, 93.8, 122.0, 131.9, 137.0, 139.7, 150.2; *m/z* EI MS+ 689.89, $[M]^+$, $C_{21}H_{24}I_2O_6S_2^+$ requires 689.91 (100%).

1,1'-((2,7-Diiodo-9H-fluorene-9,9-diyl)bis(propane-3,1-diyl)bis(4-(((4-((*tert*-butoxycarbonyl)amino)butanoyl)oxy)methyl)pyridin-1-ium)propyl)methanesulfonate (8)

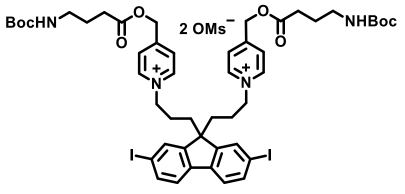

This compound was synthesized by adapting a reported procedure.[S11] A solution of (2,7-diiodo-9H-fluorene-9,9-diyl)bis(propane-3,1-diyl dimethanesulfonate) (**6**) (300 mg, 0.43 mmol) and pyridin-4-ylmethyl 4-((*tert*-butoxycarbonyl)amino)butanoate (**7**) (319 mg, 1.09 mmol) was refluxed in dry MeCN (5.0 mL) for 36 h. The solution became a deep red color and NMR spectroscopy showed the reaction was complete. The solvent was concentrated and the residue taken up in H_2O. The aqueous solution was washed with toluene (3 × 5 mL) and $CHCl_3$ (3 × 5 mL) to remove the excess starting reagent, then concentrated *in vacuo* to give compound **8** as a red solid (CARE: Very hygroscopic) (457 mg, 0.37 mmol, 86%).

^{1}H NMR (400 MHz, $CDCl_3$) *δ*: 1.30–1.43 (m, 22H), 1.77–1.84 (m, 4H), 2.03–2.10 (m, 4H), 2.49 (t, *J*=7.3 Hz, 4H), 2.69 (s, 6H), 3.09–3.14 (m, 4H), 4.52–4.59 (m, 4H), 5.26–5.28 (m, 2H), 5.36 (s, 4H), 7.37 (d, *J*=7.9 Hz, 2H), 7.60–7.63 (m, 4H), 7.98 (d, *J*=6.3 Hz, 4H), 8.95 (d, *J*=6.3 Hz, 4H); ^{13}C NMR (100 MHz, $CDCl_3/CD_3CN$) *δ*: 25.0, 26.1, 28.2, 30.8, 34.4, 39.4, 39.5, 53.9, 60.9, 63.0, 93.6, 116.7, 121.9, 125.5, 132.1, 136.9, 139.3, 144.6, 150.1, 156.1, 156.3, 172.3.

BEF-Pyr-(Boc)GABA (9)

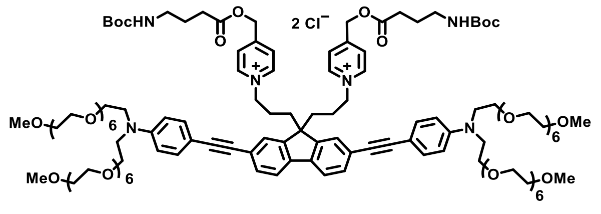

This compound was synthesized by adapting a reported procedure.[S7] The following solids were dried in Schlenk tube under vacuum: 1,1'-((2,7-diiodo-9H-fluorene-9,9-diyl)bis(propane-3,1-diyl))bis(4-(((4-((*tert*-butoxycarbonyl)amino)butanoyl)oxy)methyl)pyridin-1-ium) methanesulfonate (**8**) (114 mg, 0.09 mmol), $Pd(OAc)_2$ (1.00 mg, 4 μmol), PPh_3 (2.10 mg, 8 μmol) and CuI (0.76 mg, 4 μmol) in DIPA (2 mL). A solution of *N,N*-di-(2,5,8,11,14,17,20-heptaoxadocosan-22-yl)-*N*-(4-ethynylphenyl)-amine (**4**) (149 mg, 0.20 mmol) in dry MeCN (2.0 mL) was transferred to the Schlenk tube and the resulting solution was freeze-thaw degassed three times and allowed to warm to 20 °C. After 1 h, HPLC analysis showed a single peak at 14.8 min (*HPLC Method 3*) with the desired absorption spectrum. The reaction mixture was concentrated *in vacuo* and the residue taken up in H_2O. The aqueous suspension was washed with toluene (3 × 10 mL) to remove PPh_3, then a small volume of brine was added and the solution washed with $CHCl_3$ until the aqueous phase became colorless. The organic washings were combined, dried over magnesium sulfate, filtered and the solvents removed *in vacuo* to afford **BEF-Pyr-(Boc)GABA 9** as a dark red oil (150 mg, 0.062 mmol, 69% yield). NMR spectra showed that the methanesulfonate counterion had been removed during the work-up. Considering the catalytic amount of CuI used in the reaction, we assume that the chloride counterion came from a brine wash.

^1H NMR (400 MHz,CD_3CN) δ: 1.14–1.23 (m, 4H), 1.39 (s, 18H), 1.70–1.77 (m, 4H), 2.12–2.12–2.16 (m, 4H), 2.44 (t, *J*=7.4 Hz, 4H), 3.03–3.09 (m, 4H), 3.29 (s, 12H), 3.46–3.66 (m, 112H), 4.26 (t, *J*=7.4 Hz, 4H), 5.30 (s, 4H), 5.37–5.48 (m, 2H), 6.79 (d, *J* = 8.9 Hz, 4H), 7.35 (d, *J* = 8.9 Hz, 4H), 7.42 (s, 2H), 7.50 (dd, *J*=1.1 Hz, *J* = 7.9 Hz, 2H), 7.75 (d, *J* = 7.9 Hz, 2H), 7.9 (d, *J* = 6.4 Hz, 4H), 8.40 (d, *J* = 6.4 Hz, 4H); ^{13}C NMR (125 MHz, CD_3CN) 18.1, 25.0, 25.7, 27.7, 30.5, 35.1, 39.2, 50.5, 53.9, 57.9, 60.8, 62.9, 68.0, 68.1, 69.9, 70.1, 70.2, 70.2, 70.4, 71.6, 78.2, 88.0, 91.6, 108.7, 111.9, 120.4, 123.3, 125.3, 125.5, 131.0, 132.6, 140.0, 144.1, 148.4, 148.5, 156.1, 157.0, 172.4;

BEF-Pyr-GABA

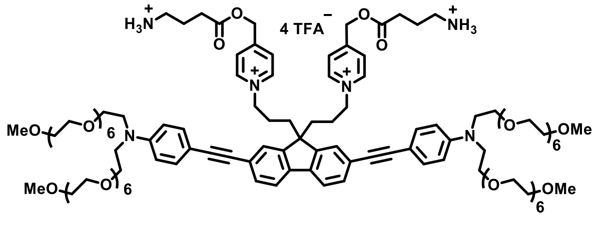

This compound was synthesized by adapting a reported procedure.[S12] **BEF-Pyr-(Boc)GABA 9** (30 mg, 11 μmol) was dissolved in dry DCM (2 mL) in a flame-dried round-bottomed flask with approximately eight 4-Å molecular sieves. The mixture was cooled to 0 °C and $BF_3{\cdot}Et_2O$ (56 μL, 0.58 mmol) was added slowly *via* syringe. The mixture was stirred vigorously for 5 h, after which time, HPLC analysis showed no starting material at 14 min and a new peak at 10.6 min (*HPLC Method 3*). The reaction was quenched with saturated aqueous $NaHCO_3$ and washed with DCM (3 × 5 mL). The organic washings were combined, dried over $MgSO_4$, filtered and concentrated *in vacuo*. The residue was taken up in the

minimum amount of water and purified by semi-preparative HPLC (*HPLC Method 3*) to afford **BEF-Pyr-GABA** as a yellow oil (3 mg, 1.1 μmol, 10%).

^{1}H NMR (500 MHz, D_2O) δ: 1.05–1.11 (m, 4H), 1.78–1.84 (m, 4H), 2.03–2.09 (m, 4H), 2.39 (t, *J*=7.3 Hz, 4H), 2.89–2.92 (m, 4H), 3.26 (s, 12H), 3.48–3.51 (m, 8H), 3.54–3.64 (m, 96H), 3.69–3.72 (m, 8H), 4.16–4.19 (m, 4H), 5.29 (s, 4H), 6.83 (d, *J*=8.9 Hz, 4H), 7.04 (s, 2H), 7.39 (d, *J*=8.9 Hz, 4H), 7.47 (d, *J*=8.0 Hz, 2H), 7.77 (d, *J*=8.0 Hz, 2H), 7.81 (d, *J*=6.5 Hz, 4H), 8.34 (d, *J*=6.5 Hz, 4H); ^{19}F NMR (470 MHz, D_2O) δ: –75.7; *m/z* ESI-MS+ 1078.1286, $[M-2H]^{2+}$, $C_{115}H_{178}N_6O_{32}{}^{2+}$ requires 1078.1254 (100%);

1,1'-((2,7-Diiodo-9H-fluorene-9,9-diyl)bis(propane-3,1-diyl)bis(4-(((4-((*tert*-butoxycarbonyl) tryptophyl)oxy)methyl)pyridin-1-ium)propyl) methanesulfonate (11)

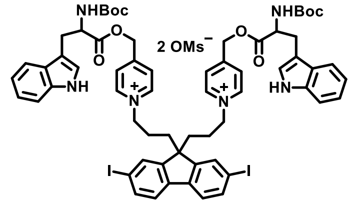

This compound was synthesized by adapting a reported procedure.[S11] (2,7-Diiodo-9H-fluorene-9,9-diyl)bis(propane-3,1-diyl dimethanesulfonate) (**6**) (250 mg, 0.36 mmol), pyridin-4-ylmethyl (*tert*-butoxycarbonyl)tryptophanate (**10**) (426 mg, 1.08 mmol) and molecular sieves were placed in as flask and dried under vacuum. MeCN (5.0 mL) was added and the reaction mixture was heated at 100 °C under N_2 atmosphere. After 12 h the mixture was allowed to cool down to 20 °C and filtered. Collected liquid was evaporated to dryness. Resulting oil was dissolved in MeCN and product was precipitated with Et_2O. Precipitant was decanted and sonicated with Et_2O. The procedure of precipitation and sonication was repeated until the compound **11** formed red solid (320 mg, 0.22 mmol, 60% yield).

^{1}H NMR (500 MHz, DMSO-*d$_6$*) δ: 1.01–1.08 (m, 4H), 1.35 (s, 18H), 1.99–2.04 (m, 4H), 2.32 (s, 6H), 3.12 (dd, *J*=8.7 Hz, *J*=14.3 Hz, 2H), 3.18 (dd, *J*=6.5Hz, J=14.3 Hz, 2H), 4.30–4.40 (m, 6H), 5.31 (d, *J*=16.1 Hz, 2H), 5.38 (d, *J*=16.1 Hz, 2H), 6.94–6.97 (m, 2H), 7.00–7.03 (m, 2H), 7.20 (s, 2H), 7.30 (d, *J*=7.8 Hz, 2H), 7.49–7.52 (m, 4H), 7.66–7.77 (m, 10H), 8.78 (d, *J*=6.5 Hz, 4H), 10.89 (s, 2H); ^{13}C NMR (125 MHz, DMSO-*d$_6$*) δ: 26.0, 27.1, 28.5, 34.8, 40.3, 54.4, 55.3, 60.3, 63.7, 78.8, 94.7, 109.7, 112.0, 118.4, 118.8, 121.3, 122.8, 124.4, 125.0, 127.4, 132.1, 136.5, 136.9, 139.6, 144.5, 150.5, 155.8, 155.9, 172.6; *m/z* ESI-MS+ 645.3, $[M]^{2+}$, $C_{63}H_{68}I_2N_6O_8{}^{2+}$ requires 645.1 (100%).

BEF-Pyr-(Boc)Trp 12

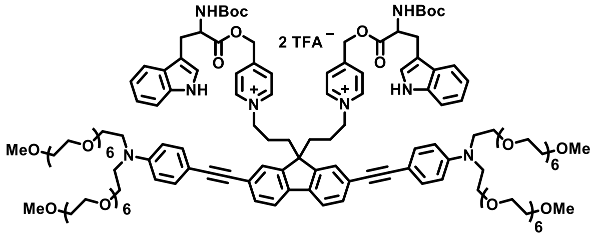

This compound was synthesized by adapting a reported procedure.[S7] $Pd(OAc)_2$ (1.2 mg, 6 µmol), PPh_3 (2.8 mg, 0.01 mmol) and CuI (0.8 mg, 6 µmol) were placed in a Schlenk tube and dried under vacuum. 1,1'-((2,7-Diiodo-9H-fluorene-9,9-diyl)bis(propane-3,1-diyl)bis(4-(((4-((*tert*-butoxycarbonyl) tryptophyl)oxy)methyl)pyridin-1-ium)propyl) methanesulfonate (**11**) (80 mg, 54 µmol) was dissolved in mixture of MeCN and CH_2Cl_2 (0.2 mL, 1:1, v/v) and transferred to the Schlenk tube. DIPA (0.2 mL) was added and the reaction mixture was degassed by two freeze-thaw cycles. A solution of *N,N*-di-(2,5,8,11,14,17,20-heptaoxadocosan-22-yl)-*N*-(4-ethynylphenyl)-amine (**4**) (94 mg, 0.12 mmol) in MeCN (0.1 mL) was added to the reaction mixture and additional freeze-thaw cycle was performed. Reaction was stirred at 20 °C and its progress was monitored by HPLC (*HPLC Method 1*). After 2 h the mixture was diluted with EtOAc (20 mL), organic phase was washed with H_2O (20 mL), followed by saturated aqueous solution of NH_4Cl (20 mL) and H_2O (20 mL). Organic layer was dried over $MgSO_4$ and evaporated. Product was isolated with use of reverse phase column chromatography (gradient from 100% H_2O with 0.1% TFA to 100% acetonitrile) to give 50 mg (0.017 mmol, 33% yield) of **BEF-Pyr-(Boc)Trp 12** as an oil. It is assumed that methanefulfonate counterion was replaced with TFA anion during the column chromatography with TFA-buffer. NMR spectra confirmed the absence of methanesulfonate.

^1H NMR (500 MHz, DMSO-d_6) δ: 1.12–1.27 (m, 4H), 1.37 (s, 18H), 2.11–2.25 (m, 4H), 3.13–3.22 (m, 4H), 3.29 (s, 12H), 3.34–3.63(m, 112H), 4.38–4.50 (m, 6H), 5.32 (d, *J*=16.5 Hz, 2H), 5.38 (d, *J*=16.5 Hz, 2H), 6.79 (d, *J*=8.5 Hz, 4H), 6.97–7.00 (m, 2H), 7.05–7.08 (m, 2H), 7.23 (s, 2H), 7.34–7.39 (m, 6H), 7.51–7.59 (m, 8H), 7.77 (d, *J*=6.3 Hz, 4H), 7.90 (d, *J*=7.9 Hz, 2H), 8.88 (d, *J*=6.3Hz, 4H), 11.02 (s, 2H); ^{13}C NMR (125 MHz, DMSO-d_6) δ: 26.2, 27.0, 28.4, 35.3, 50.4, 54.1, 55.2, 58.4, 60.4, 63.6, 68.1, 69.9, 70.1–70.2, 70.3, 71.6, 78.9, 88.6, 92.1, 108.3, 109.6, 111.8, 118.3, 118.7, 121.0, 121.3, 122.9, 124.3, 124.9, 125.6, 127.3, 131.2, 132.8, 136.4, 139.8, 144.5, 148.3, 149.0, 155.8, 155.9, 172.6.

BEF-Pyr-Trp

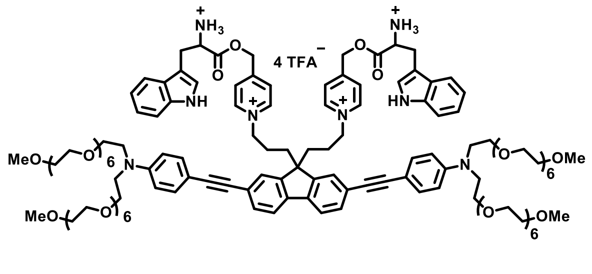

This compound was synthesized by adapting a reported procedure.[S13] Triisopropylsilane (0.1 mL) was added to a solution of **BEF-Pyr-(Boc)Trp 12** (15 mg, 5.3 µmol) in formic acid (1 mL) and reaction was stirred at 20 °C. The progress of the reaction was monitored by HPLC (*HPLC Method 1*) and after 2 h, the solvent was evaporated under a stream of nitrogen. **BEF-Pyr-Trp** was isolated from the crude reaction mixture by semi-preparative HPLC (*HPLC Method 1*). Product containing fractions were combined and freeze-dried. 1.08 mg of **BEF-Pyr-Trp** (0.4 µmol, 8% yield) was obtained.

^{1}H NMR (500 MHz, DMSO-d_6) δ: 0.96–1.05 (m, 4H), 2.07–2.17 (m, 4H), 3.16 (s, 12H), 3.24 (under H_2O, 4H), 3.34–3.58 (m, 112H), 4.25–4.33 (m, 4H), 4.36–4.42 (m, 2H), 5.26 (d, J=16.7 Hz, 2H), 5.32 (d, J=16.7 Hz, 2H), 6.67 (d, J=8.7 Hz, 4H), 6.81–6.85 (m, 2H), 6.89–6.93 (m, 2H), 7.15–7.17 (m, 2H), 7.21 (d, J=8.4 Hz, 2H), 7.25 (d, J=8.7 Hz, 4H), 7.36 (d, J=7.8 Hz, 2H), 7.43 (d, J=8.1 Hz, 2H), 7.51 (s, 2H), 7.61 (d, J=6.7 Hz, 4H), 7.78 (d, J=8.1 Hz, 2H), 8.42 (bs, 6H), 8.68 (d, J=6.7 Hz, 4H), 10.95 (s, 2H); *m/z* ESI-MS+ 590.0792, $[M]^{4+}$, $C_{129}H_{186}N_8O_{32}^{4+}$ requires 590.0797 (100%).

1.2.3 Phenacyl group

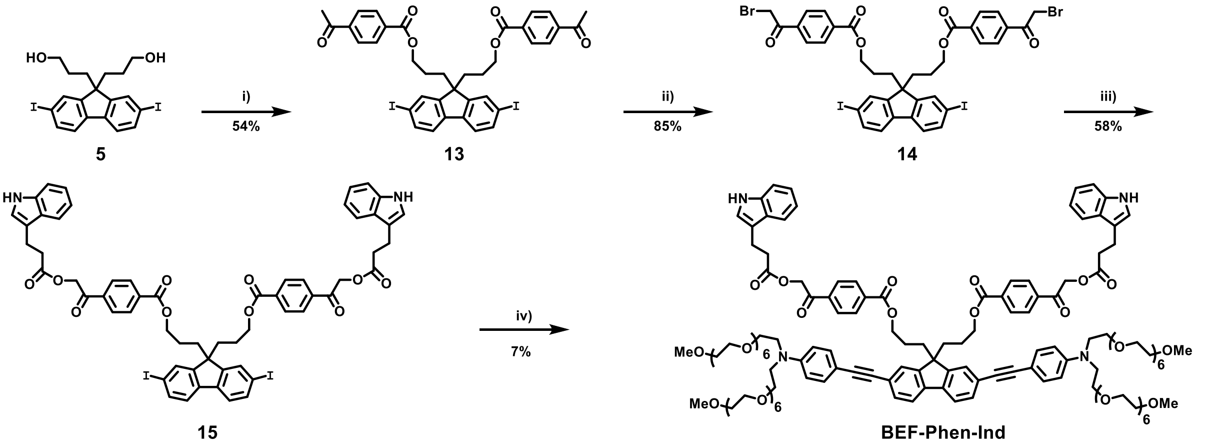

Scheme S3. Reagents: *i)* 4-acetylbenzoic acid, EDC, DMAP, THF, MeCN, 20 °C, 16 h, *ii)* Br_2, $CHCl_3$, 0 °C → 20 °C, *iii)* 3-indolepropionic acid, DBU, THF, 20 °C, 16 h, *iv)* **4**, $Pd(OAc)_2$, PPh_3, CuI, DIPA, DCM, 20 °C, 3 h.

(2,7-Diiodo-9H-fluorene-9,9-diyl)bis(propane-3,1-diyl) bis(4-acetylbenzoate) (13)

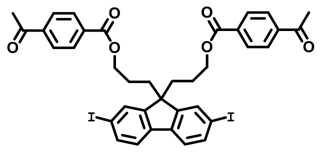

This compound was synthesized by adapting a reported procedure.[S14] 3,3'-(2,7-Diiodo-9H-fluorene-9,9-diyl)bis(propan-1-ol) (**5**) (300 mg, 0.56 mmol), 4-acetylbenzoic acid (200 mg, 1.22 mmol), EDC (234 mg, 1.23 mmol) and DMAP (25 mg, 0.20 mmol) were dissolved in anhydrous DCM and stirred for 2 h at 20 °C. The mixture was diluted with water and extracted with DCM (50 mL). Organic extracts were combined and dried over $MgSO_4$ and evaporated to dryness. The residue was dissolved in $CHCl_3$ and passed through a silica plug with $CHCl_3$ as eluent. Product containing fractions were combined and evaporated *in vacuo*. The product was purified further by dissolving in MeOH and precipitating with Et_2O/hexane (1:1 v/v) to give compound **13** as a white solid in 54% yield (250 mg, 0.3 mmol).

^{1}H NMR (400 MHz, $CDCl_3$) *δ*: 1.04–1.16 (m, 4H), 2.13–2.17 (m, 4H), 2.65 (s, 6H), 4.07 (t, *J*=6.2 Hz, 4H), 7.47 (d, *J*=8.4 Hz, 2H), 7.72–7.73(m, 4H), 8.02 (d, *J*=8.7 Hz, 4H), 8.06 (d, *J*=8.7 Hz, 4H); ^{13}C NMR (100 MHz, $CDCl_3$) *δ*: 22.1, 25.9, 35.3, 53.6, 63.9, 92.5, 120.9, 127.3, 128.8, 130.8, 132.9, 135.9, 138.9, 139.2, 149.7, 164.5, 196.5; *m/z* EI^+ 826.02, $[M]^+$, $C_{37}H_{32}I_2O_6^+$ requires 826.02 (100%).

(2,7-Diiodo-9H-fluorene-9,9-diyl)bis(propane-3,1-diyl) bis(4-(2-bromoacetyl)benzoate) (14)

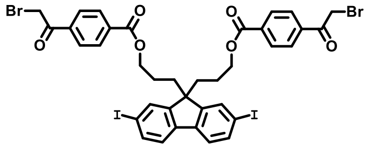

This compound was prepared by analogy with a reported procedure.[S15] To the cooled to 0 °C solution of 2,7-diiodo-9H-fluorene-9,9-diyl)bis(propane-3,1-diyl) bis(4-acetylbenzoate) (**13**) (550 mg, 0.6 6mmol) in $CHCl_3$ (11 mL), a solution of bromine (72 µL, 1.39 mmol) in $CHCl_3$ (5 mL) was added dropwise. Reaction was stirred at 20 °C until the color changed from intensively red to pale yellow. Crude mixture was washed with $NaHCO_3$, followed by washing with H_2O. Organic phase was dried over $MgSO_4$ and evaporated to dryness. The product was purified by column chromatography on silica (DCM : MeOH in gradient 0% to 5% of MeOH) to give 556 mg (0.56 mmol, 85%) of compound **14** as a white solid.

^{1}H NMR (400 MHz, $CDCl_3$) *δ*: 0.96–1.03 (m, 4H), 2.05–2.09 (m, 4H),4.00 (t, *J*=6.3 Hz, 4H), 4.38 (s, 4H), 7.39 (d, *J*=8.6 Hz, 2H), 7.64–7.65 (m, 4H), 7.97–8.02 (m, 8H); ^{13}C NMR (100 MHz, $CDCl_3$) δ: 23.2, 30.6, 36.3, 54.6, 65.0, 93.6, 122.0, 129.0, 130.0, 131.8, 134.6, 136.9, 137.1, 139.9, 150.7, 165.2, 190.8; *m/z* EI-MS+ 983.8 $[M]^+$, $C_{37}H_{30}Br_2I_2O_6^+$ requires 983.8 (100%).

(2,7-Diiodo-9H-fluorene-9,9-diyl)bis(propane-3,1-diyl)bis(4-(2-((3-(1H-indol-3-yl) propanoyl)oxy)acetyl)benzoate) (15)

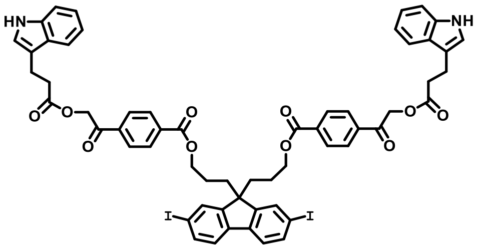

This compound was prepared by analogy with a reported procedure.[S16] To a solution of (2,7-diiodo-9H-fluorene-9,9-diyl)bis(propane-3,1-diyl) bis(4-(2-bromoacetyl)benzoate) (**14**) (100 mg, 0.1 mmol) in THF (0.5 mL) a solution of 3-indolepropionic acid (38 mg, 0.2 mmol) in THF (2.0 mL) was added followed by DBU (32 µL, 0.21 mmol). After 16 h of stirring at 20 °C the solvent was evaporated *in vacuo* and the product was isolated from the crude reaction mixture with use of the column chromatography ($CHCl_3$ – MeOH, 99:1 v/v). Compound **15** was obtained as white solid in 58% yield (70 mg, 0.058 mmol).

^{1}H NMR (400 MHz, $CDCl_3$) δ: 0.94–1.04 (m, 4H), 2.03–2.10 (m, 4H), 2.83 (t, J=7.7 Hz, 4H), 3.10 (t, J=7.6 Hz, 4H), 3.99 (t, J=6.2 Hz, 4H), 5.25 (s, 4H), 6.97 (s, 2H), 7.01–7.07 (m, 2H), 7.09–7.14 (m, 2H), 7.27 (d, J=8.2 Hz, 2H), 7.40 (d, J=7.9, 2H), 7.54 (d, J=7.7 Hz, 2H), 7.62–7.67 (m, 4H), 7.89 (d, J=8.2 Hz, 4H), 7.99 (d, J=8.2 Hz, 4H); ^{13}C NMR (100 MHz, $CDCl_3$) δ: 20.5, 23.2, 34.5, 36.3, 54.6, 65.0, 66.1, 93.6, 111.1, 114.8, 118.7, 119.4, 121.5, 121.9, 122.1, 127.2, 127.8, 130.0, 131.8, 134.6, 136.3, 136.9, 137.4, 140.0, 150.7, 165.3, 172.7, 192.1; *m/z* MALDI TOF^{+} 1201.4, $[M+H]^{+}$, $C_{59}H_{51}I_2N_2O_{10}^{+}$, requires 1201.1 (100%).

BEF-Phen-Ind

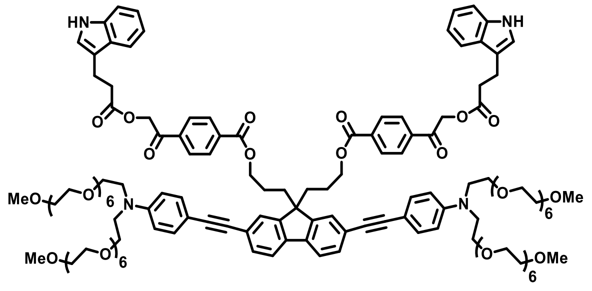

This compound was synthesized by adapting a reported procedure.[S7] To vacuum-dried CuI (1.1 mg, 6 µmol), $Pd(OAc)_2$ (1.2 mg, 5 µmol) and PPh_3 (3.0 mg, 11 µmol), (2,7-diiodo-9H-fluorene-9,9-diyl)bis(propane-3,1-diyl)bis(4-(2-((3-(1H-indol-3-yl)propanoyl)oxy) acetyl)benzoate) (**15**) (70 mg, 0.058 mmol) in DCM (1 mL) and distilled DIPA (1.0 mL) was added and two freeze-thaw cycles were carried out. A solution of *N,N*-di-(2,5,8,11,14,17,20-heptaoxadocosan-22-yl)-*N*-(4-ethynylphenyl)-amine (**4**) (93 mg, 0.122 mmol) in DCM (1 mL) was added and additional freeze-thaw cycle was run. The progress of the reaction was monitored by HPLC (*HPLC Method 2*). After 2 h of stirring at 20 °C the reaction mixture was diluted with $CHCl_3$ (50 mL) and crude mixture was washed with H_2O (20 mL) and NH_4Cl (20 mL). The organic layer was dried over $MgSO_4$ and evaporated to dryness. The crude reaction mixture was dissolved in MeCN and passed through a pre-packed reverse phase column with MeCN as eluent. The organic phase was collected and solvent was removed *in vacuo* to give 180 mg of the crude reaction mixture. 10 mg of the crude mixture were purified with use of semi-prep HPLC to give 0.6 mg of **BEF-Phen-Ind** as a yellow oil (7% yield calculated for the 10 mg of purified crude, 0.24 µmol).

^{1}H NMR (400 MHz, $CDCl_3$) δ: 0.92–1.00 (4H, m), 2.25–2.33 (m, 4H), 2.82 (t, J=7.7 Hz, 4H), 3.01 (t, J=7.6 Hz, 4H), 3.22 (s, 12H), 3.39–3.42 (under H_2O, m, 8H), 3.47–3.59 (m, 104 H), 3.99–4.04 (m, 4H), 5.36 (s, 4H), 6.72 (d, J=8.7 Hz, 4H), 6.97–7.00 (m, 2H), 7.06–7.09 (m, 2H), 7.16–7.19 (m, 2H), 7.29–7.34 (m, 6H), 7.49–7.53 (m, 4 H), 7.69 (s, 2H), 7.89 (d, J=7.8 Hz, 2H), 8.00–8.04 (m, 8H), 10.83 (s, 2H); *m/z* MALDI TOF^{+} 2468.81, $[M]^{+}$, $C_{135}H_{182}N_4O_{38}^{+}$ requires 2468.25 (100%); 2491.92, $[M+Na]^{+}$, $C_{135}H_{182}N_4O_{38}Na^{+}$ requires 2491.24 (100%); 2507.90, $[M+K]^{+}$, $C_{135}H_{182}N_4O_{38}K^{+}$ requires 2507.21 (100%).

1.2.4 *o*-Nitrobenzyl group

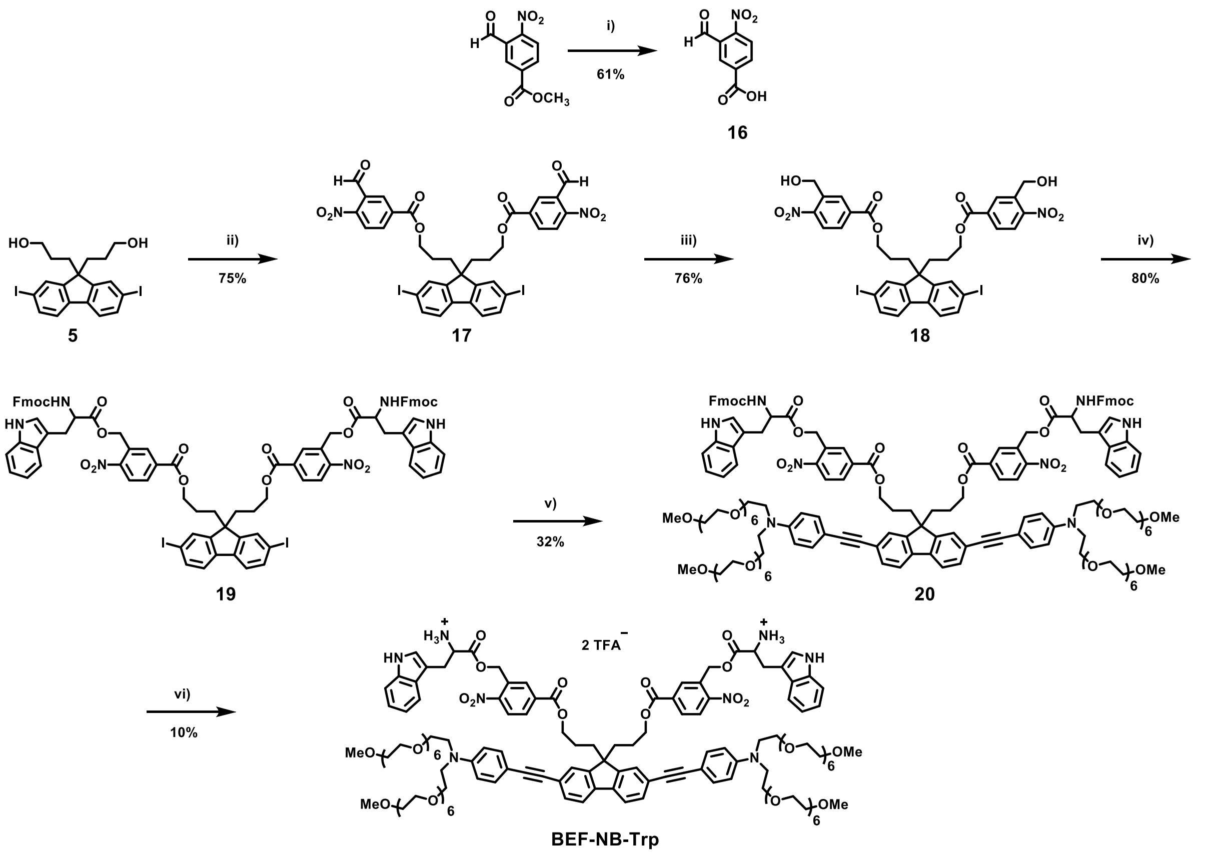

Scheme S4. Reagents: *i)* conc. H_2SO_4, H_2O, 100 °C, 24 h, *ii)* **16**, EDC, DMAP, DCM, 20 °C, 2 h, *iii)* $NaBH_4$, MeOH, 0 °C → 20 °C, *iv)* Fmoc-Trp-OH, EDC, DMAP, DCM, 20 °C, 48 h, *v)* **4**, $Pd(OAc)_2$, PPh_3, CuI, DIPA, DCM, 20 °C, 2 h, *vi)* piperidine, MeCN, 20 °C, 2 h, then purified with 0.1% TFA solvent system.

3-Formyl-4-nitrobenzoic acid (16)

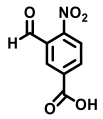

This compound was synthesized by adapting a reported procedure.[S17] To a suspension of methy-3-formyl-nitrobenzoate (0.70 g, 3.35 mmol) in H_2O (5 mL) conc. H_2SO_4 (0.5 mL) was added and the reaction mixture was stirred at 100 °C for 24 h. After this time reaction was allowed to cool, the precipitate was filtered, washed thoroughly with H_2O and dried. Compound **16** was obtained as a white powder in 61% yield (0.40 g, 2.0 mmol).

^{1}H NMR (250 MHz, DMSO-d_6) δ: 8.24 (d, J=8.1 Hz, 1H), 8.37–8.40 (m, 2H), 10.25 (s, 1H); ^{13}C NMR (62 MHz, DMSO-d_6) δ: 125.8, 131.1, 131.6, 135.6, 136.2, 152.0, 166.0, 190.1.

(2,7-Diiodo-9H-fluorene-9,9-diyl)bis(propane-3,1-diyl) bis(3-formyl-4-nitrobenzoate) (17)

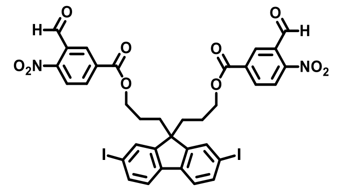

This compound was synthesized by adapting a reported procedure.[S14] 3,3'-(2,7-Diiodo-9H-fluorene-9,9-diyl)bis(propan-1-ol) (**5**) (200 mg, 0.37 mmol), 3-formyl-4-nitrobenzoic acid (**16**) (160 mg, 0.82 mmol), EDC (156 mg, 0.81 mmol) and DMAP (9 mg, 0.07 mmol) were dissolved in anhydrous DCM (5 mL) and stirred for 2 h at 20 °C. The mixture was diluted with water and extracted with DCM (50 mL). The organic extracts were combined and dried over $MgSO_4$ and evaporated to dryness. The residue was dissolved in $CHCl_3$ and passed through a silica plug with $CHCl_3$ as eluent. The product containing fractions were combined and evaporated *in vacuo*. The product was further purified by dissolving in DCM and precipitating with PE to give compound **17** as a white solid in 75% yield (250 mg, 0.28 mmol).

^{1}H NMR (500 MHz, $CDCl_3$) *δ:* 1.10–1.16 (m, 4H), 2.18–2.20 (m, 4H), 4.15 (t, *J*=6.23 Hz, 4H), 7.52 (d, *J*=7.8 Hz, 2H), 7.73–7.77 (m, 4H), 8.21 (d, *J*=8.6 Hz, 2H), 8.36 (dd, *J*=1.6 Hz, *J*=8.6 Hz, 2H), 8.53 (d, *J*=1.6 Hz, 2H), 10.44 (s, 2H); ^{13}C NMR (125 MHz, $CDCl_3$) *δ:* 23.1, 36.1, 54.7, 65.9, 93.8, 122.2, 124.9, 131.0, 131.2, 131.8, 134.6, 135.2, 137.0, 140.0, 150.5, 151.7, 163.5, 187.1; *m/z* EI-MS+ 887.84, $[M]^+$, $C_{35}H_{26}I_2N_2O_{10}^+$ requires 887.96 (100%).

(2,7-Diiodo-9H-fluorene-9,9-diyl)bis(propane-3,1-diyl) bis(3-(hydroxymethyl)-4-nitrobenzoate) (18)

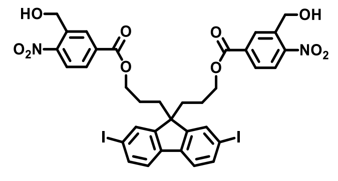

This compound was synthesized by adapting a reported procedure.[S18] $NaBH_4$ (20 mg, 0.53 mmol) was added to a cooled solution of (2,7-diiodo-9H-fluorene-9,9-diyl)bis(propane-3,1-diyl) bis(3-formyl-4-nitrobenzoate) (**17**) (250 mg, 0.28 mmol) in MeOH (3 mL) at 0 °C, and reaction mixture was stirred at 20 °C. After 15 min DCM (3 mL) was added to keep the material in solution. After an additional 1 h of stirring, DCM was evaporated and reaction was poured into the beaker with ice (250 mL). Ice was allowed to melt and aqueous phase was extracted with EtOAc (50 mL) and $CHCl_3$ (50 mL). Organic extracts were combined, dried over $MgSO_4$ and evaporated to dryness. The residue was dissolved in DCM and precipitated with hexane to give 190 mg of compound **18** as a white foam (0.21 mmol, 76% yield).

^{1}H NMR (400 MHz, $CDCl_3$) *δ:* 1.02–1.12 (m, 4H), 2.14–2.19 (m, 4H), 4.08 (m, 4H), 5.03 (s, 4H), 7.47 (d, *J*=7.9 Hz, 2H), 7.69–7.72 (m, 4H), 8.00 (d, *J*=8.7 Hz, 2H), 8.11 (d, *J*=8.7 Hz,

2H), 8.37 (s, 2H); ^{13}C NMR (100 MHz, $CDCl_3$) *δ:* 23.2, 36.3, 54.6, 61.9, 65.4, 93.7, 122.0, 125.1, 129.4, 130.6, 131.8, 134.7, 136.9, 137.2, 139.9, 149.7, 150.7, 164.6; *m/z* EI-MS+ 891.87, $[M]^+$,$C_{35}H_{30}I_2N_2O_{10}^+$ requires 891.99 (100%).

(2,7-Diiodo-9H-fluorene-9,9-diyl)bis(propane-3,1-diyl)bis(((((methoxy)carbonyl)Fmoc-tryptophyl)oxy)methyl)-4-nitrobenzoate (19)

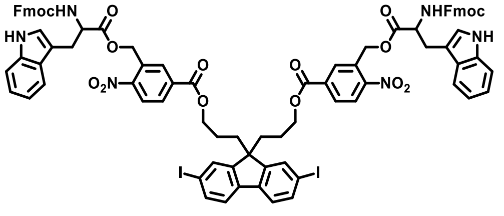

This compound was synthesized by adapting a reported procedure.[S14] (2,7-Diiodo-9H-fluorene-9,9-diyl)bis(propane-3,1-diyl)bis(3-(hydroxymethyl)-4-nitrobenzoate) (**18**) (100 mg, 0.11 mmol), Fmoc-Trp-OH (95 mg, 0.22 mmo), DMAP (3 mg, 0.025 mmol) and EDC (42 mg, 0.22 mmol) were dissolved in DCM (3 mL) and stirred at 20 °C. After 2 days the reaction mixture was diluted with DCM (30 mL) and was washed with water (50 mL). Organic layer was dried over $MgSO_4$ and evaporated to dryness. Product was isolated with use of column chromatography on silica (eluent $CHCl_3$ – MeOH, in gradient 0–2% MeOH) to give 150 mg (0.088 mmol, 80% yield) of compound **19**.

^{1}H NMR (400 MHz, $CDCl_3$) *δ:* 0.87–0.94 (m, 4H), 1.92–1.96 (m, 4H), 3.85–3.92 (m, 4H), 3.85–3.92 (m, 4H), 4.05–4.09 (m, 2H), 4.23–4.31 (m, 4H), 4.69–4.74 (m, 2H), 5.30–5.36 (m, 6H), 6.86 (s,2H), 6.95–6.99 (m, 2H), 7.02–7.05 (m, 2H), 7.12–7.19 (m, 6H), 7.24–7.31(m, 6H), 7.39–7.47 (m, 6H), 7.57–7.58 (m, 4H), 7.63 (d, *J*=7.4 Hz, 4H), 7.87 (d, *J*=8.4 Hz, 2H), 7.91–7.98 (m, 4H), 8.07 (s, 2H); ^{13}C NMR (100 MHz, $CDCl_3$) *δ:* 23.1, 28.0, 36.1, 47.1, 54.5, 54.9, 63.4, 65.4, 67.1, 93.6, 109.6, 111.3, 118.4, 119.8, 119.9, 122.0, 122.4, 123.0, 125.2, 127.1, 127.5, 127.7, 130.0, 130.9, 131.3, 131.8, 134.5, 136.0, 136.9, 139.8, 141.3, 143.7, 143.9, 150.0, 150.6, 155.8, 164.1, 171.6.

BEF-NB(Fmoc)Trp 20

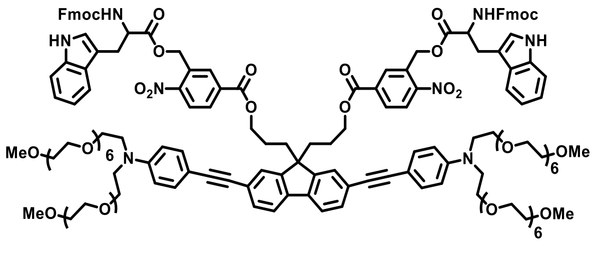

This compound was synthesized by adapting a reported procedure.[S7] To the vacuum-dried CuI (0.5 mg, 2.6 μmol), $Pd(OAc)_2$ (0.6 mg, 2.6 μmol) and PPh_3 (1.5 mg, 5.7μmol) and (2,7-diiodo-9H-fluorene-9,9-diyl)bis(propane-3,1-diyl)bis(((((methoxy)carbonyl)Fmoc-tryptophyl)oxy)methyl)-4-nitrobenzoate (**19**) (100 mg, 0.058 mmol) distilled DIPA (1.5 mL) was added and two freeze-thaw cycles were carried out. A solution of *N,N*-di-(2,5,8,11,14,17,20-heptaoxadocosan-22-yl)-*N*-(4-ethynylphenyl)-amine (**4**) (100 mg, 0.13

mmol) in anhydrous DCM (1 mL) was added and additional freeze-thaw cycle was run. The progress of the reaction was monitored by HPLC (*HPLC Method 3*). After 1 h of stirring at 20 °C the reaction mixture was diluted with DCM (50 mL) and crude mixture was washed with H_2O (20 mL) and NH_4Cl (20 mL). Organic layer was dried over $MgSO_4$ and evaporated to dryness. The major impurities were removed from the crude reaction mixture with use of the reverse phase column chromatography (in gradient from 100% of H_2O (0.1% TFA v/v) to 100% MeCN). Product containing fractions were combined and evaporated to dryness. **BEF-NB-(Fmoc)Trp 20** was obtained a yellow film (50 mg, 0.017 mmol, 32% yield)

^{1}H NMR (400 MHz, DMSO-d_6) δ: 0.88–0.95 (m, 4H), 2.11–2.20 (m, 4H), 3.17–3.24 (m, 16H), 3.39–3.42 (m, 8H), 3.47–3.58 (m, 104H), 3.87–3.91 (m, 4H), 4.10–4.16 (m, 4H), 4.21–4.26 (m, 2H), 4.34–4.38 (m, 2H), 5.37 (s, 4H), 6.69 (d, *J*=8.8 Hz, 4H), 6.92–6.95 (m, 2H), 7.00–7.05 (m, 2H), 7.15 (s, 2H), 7.19–7.30 (m, 10H), 7.34–7.38 (m, 4H), 7.45 (d, *J*=8.3 Hz, 2H), 7.50 (d, *J*=7.9 Hz, 2H), 7.56–7.62 (m, 6H), 7.80–7.83 (m, 6H), 7.91 (d, *J*=7.9 Hz, 2H), 7.98 (d, *J*=8.3 Hz, 2H), 8.05–8.09 (m, 4H), 10.81 (s, 2H); *m/z* ESI MS+ 1489.70663, $[M+2H]^{2+}$, $C_{163}H_{204}N_8O_{44}^{2+}$ requires 1489.19970 (100%).

BEF-NB-Trp

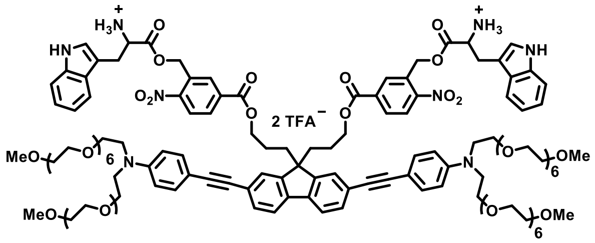

This compound was synthesized by adapting a reported procedure.[S19] To the solution of **BEF-NB-(Fmoc)Trp 20** (10 mg, 3.3 µmol) in MeCN (0.25 mL) piperidine (30 µL, 0.35 mmol) was added and reaction was stirred at 20 °C for 2 h. After this time solvent was blew down by the stream of nitrogen and product was isolated from the crude reaction mixture by semi-preparative HPLC in TFA-buffered solvent system (*HPLC Method 3*). Product containing fractions were combined and freeze-dried to give **BEF-NB-Trp** in 10% yield (0.92 mg, 0.33 µM).

^{1}H NMR (500 MHz, DMSO-d_6) δ: 0.93–1.00 (m, 4H), 2.20–2.28 (m, 4H), 3.19–3.26 (m, 16H), 3.47–3.58 (m, 112H), 3.99–4.05 (m, 4H), 4.31–4.37 (m, 2H), 5. 39 (d, *J*=13.9 Hz, 2H), 5.46 (d, *J*=13.9 Hz, 2H), 6.71 (d, *J*=8.4 Hz, 4H), 6.91–6.94 (m, 2H), 7.01–7.04 (m, 2H), 7.15 (m, 2H), 7.27–7.30 (m, 4H), 7.42 (d, *J*=7.7 Hz, 2H), 7.48 (d, *J*=8.1 Hz, 2H), 7.66 (s, 2H), 7.85 (d, *J*=7.7 Hz, 2H), 8.05–8.09 (m, 4H), 8.13 (m, 2H), 8.36 (bs, 6H), 10.97 (s, 2H); ; *m/z* ESI-MS+ 1267.1304, $[M]^{2+}$, $C_{133}H_{184}N_8O_{40}^{2+}$ requires 1267.1314 (100%).

2. NMR and MS spectra, HPLC chromatograms

In this section we present ^{1}H-NMR and ^{1}H-^{1}H COSY spectra of all final compounds. 2D NMR experiments aided assignment of ^{1}H NMR peaks to the corresponding protons in each molecule.

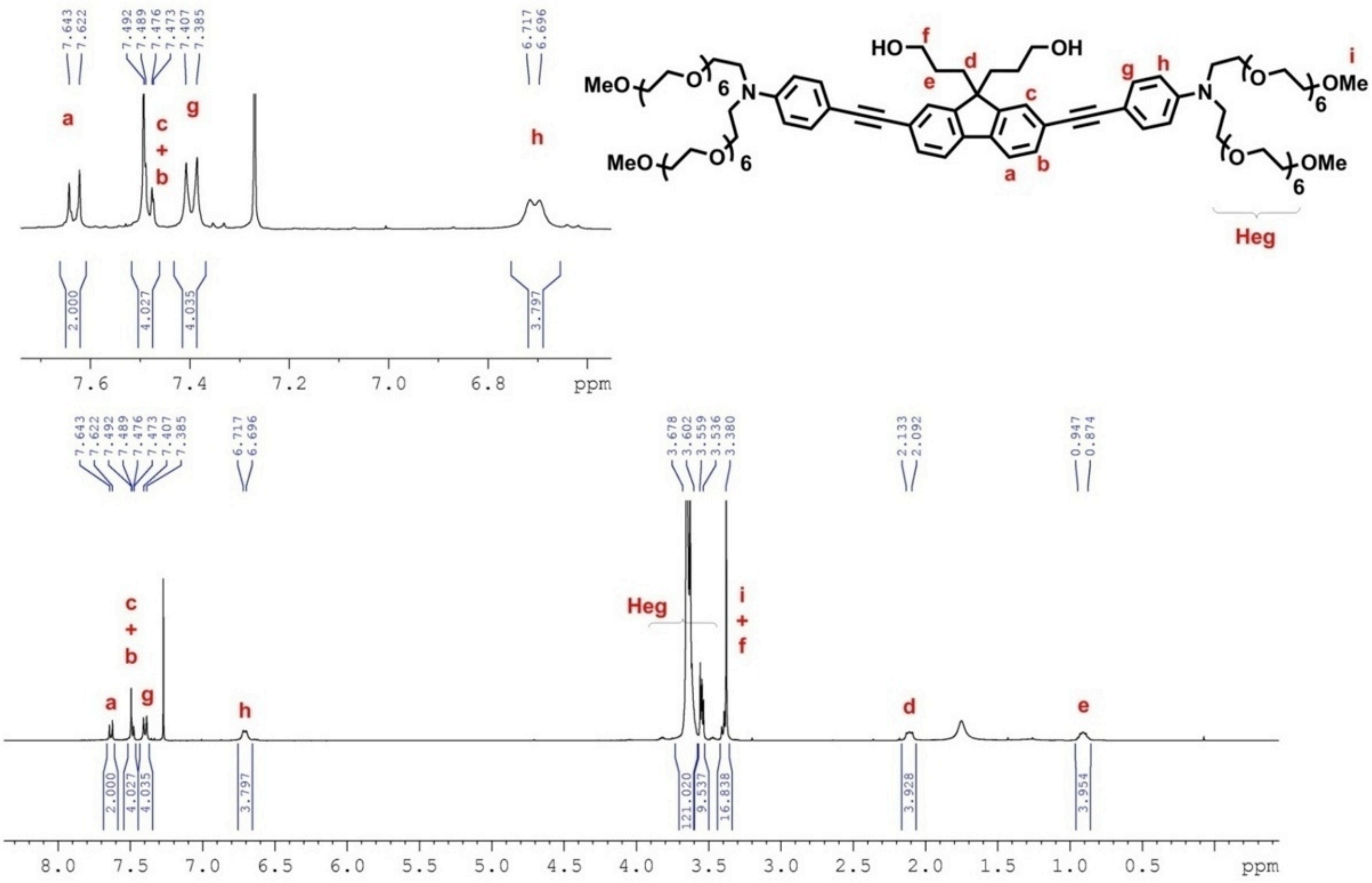

Figure S1. ^{1}H NMR spectrum of **BEF-OH** with zoom on the aromatic region (400 MHz, $CDCl_3$, 298 K).

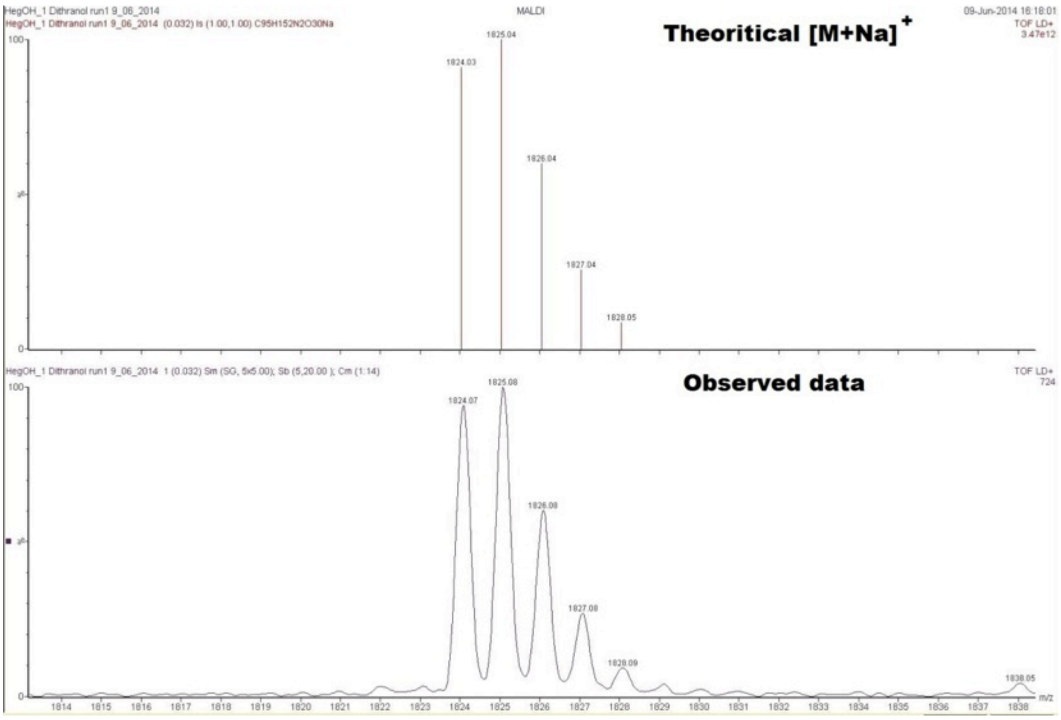

Figure S2. MALDI-TOF isotope patterning of [**BEF-OH**+Na]$^{+}$*(bottom)* and theoretical pattern calculated for $C_{95}H_{152}N_2O_{30}Na$ *(top)*.

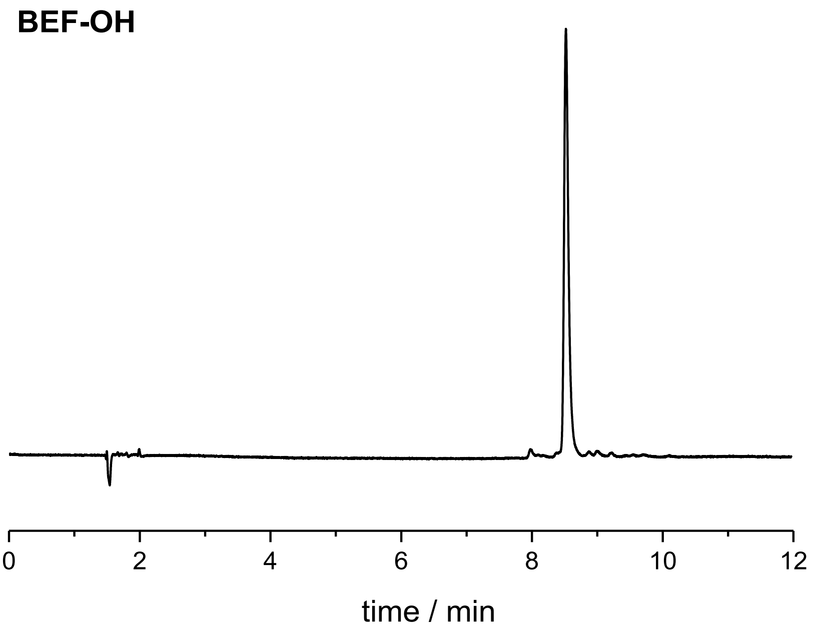

Figure S3.HPLC chromatogram of **BEF-OH**, absorption recorded at 375 nm, *HPLC method 1*. Analysis was performed with C18 5 µm, 4.6 × 150 mm Eclipse XDB-C18 column (Agilent).

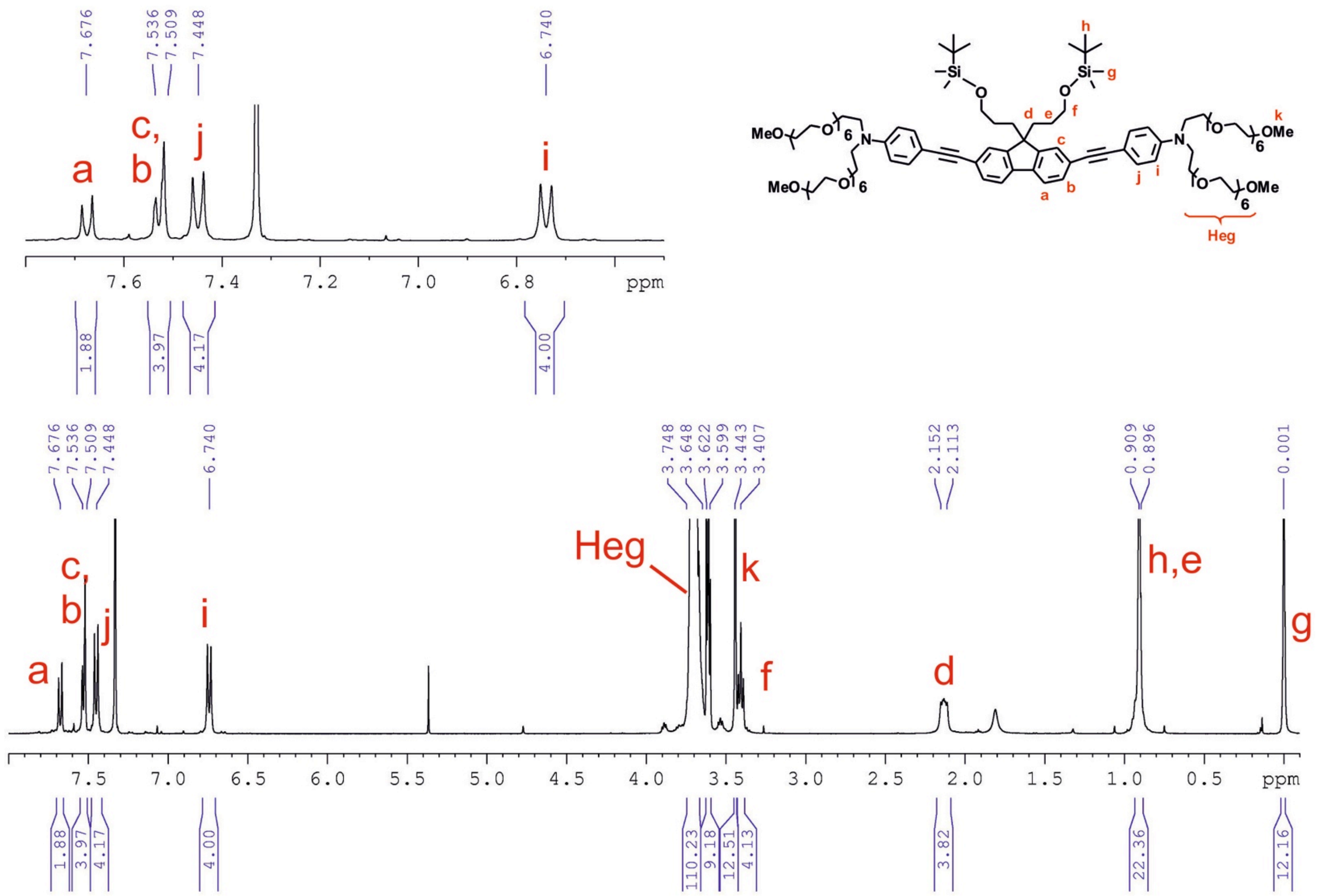

Figure S4. ^{1}H NMR spectrum of **BEF-OTBDMS** with zoom on the aromatic region (400 MHz, $CDCl_3$, 298 K).

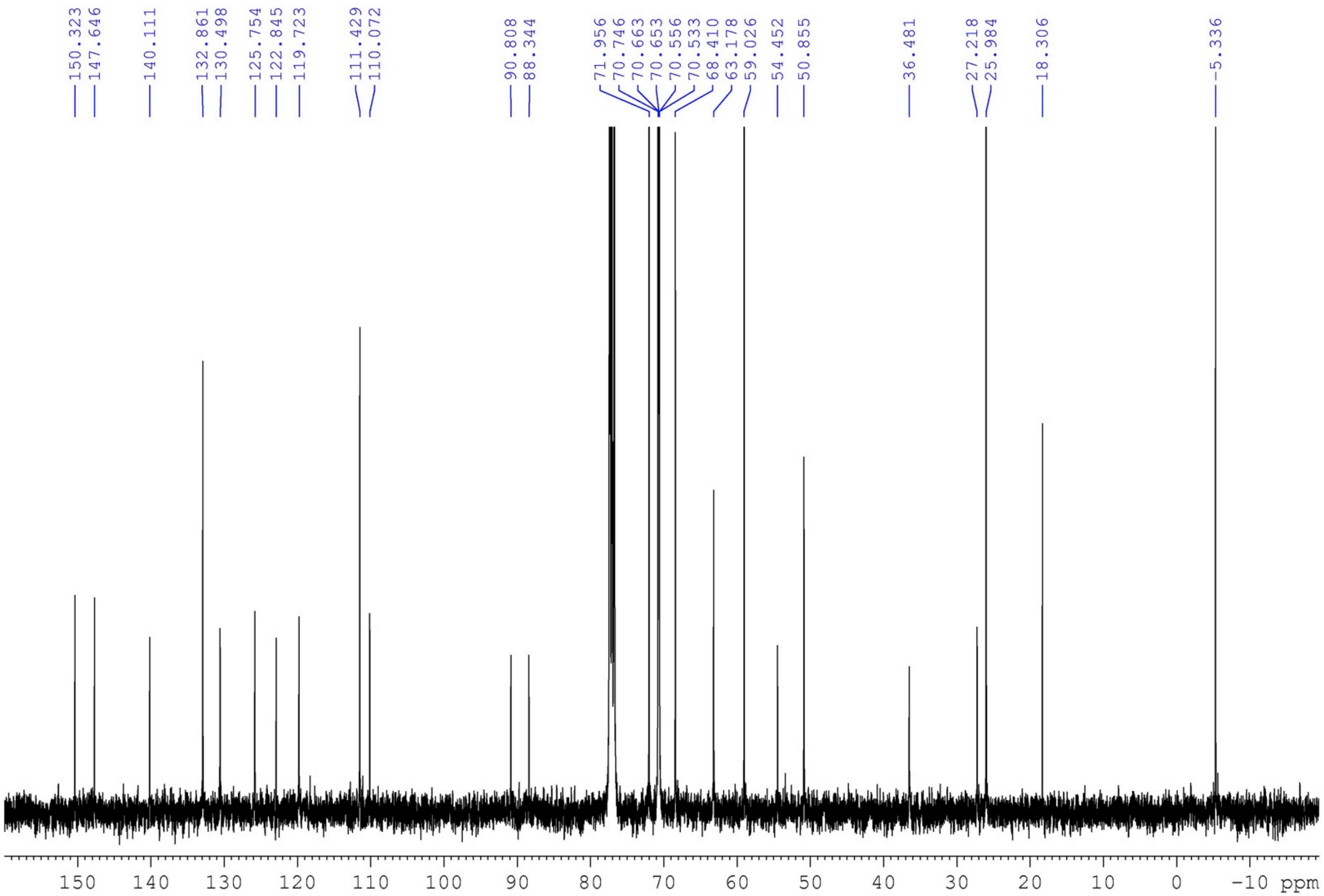

Figure S5. ^{13}C NMR spectrum of **BEF-OTBDMS** (200 MHz, $CDCl_3$, 298 K)

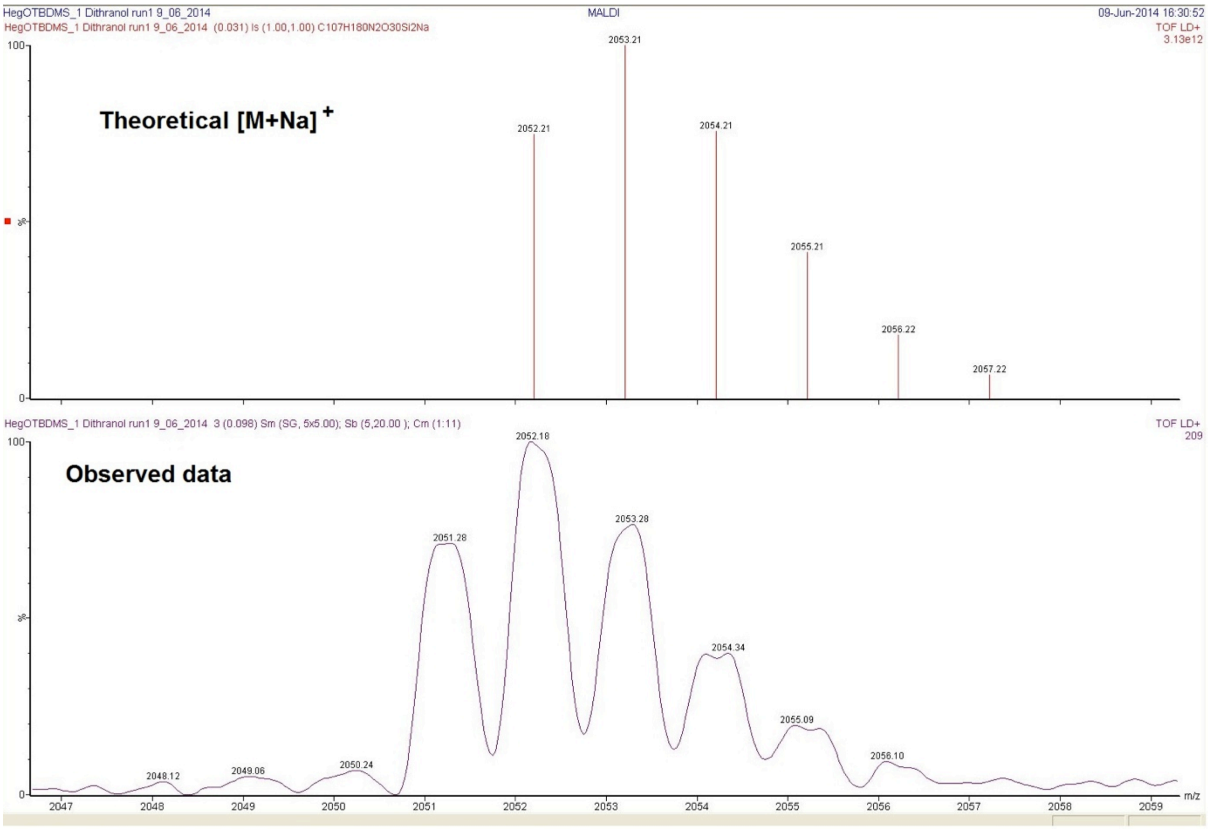

Figure S6. MALDI-TOF isotope patterning of [**BEF-OTBDMS**+Na]$^+$ *(bottom)* and theoretical pattern calculated for $C_{107}H_{180}N_2O_{30}Si_2Na^+$ *(top)*

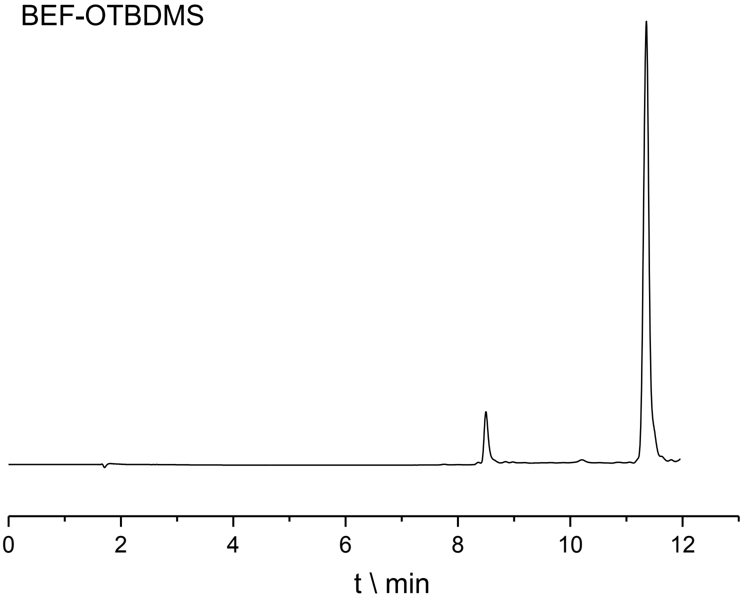

Figure S7.HPLC chromatogram of **BEF-OTBDMS**, absorption recorded at 375 nm, *HPLC method 1*. Analysis was performed with C18 5 µm, 4.6 × 150 mm Eclipse XDB-C18 column (Agilent).

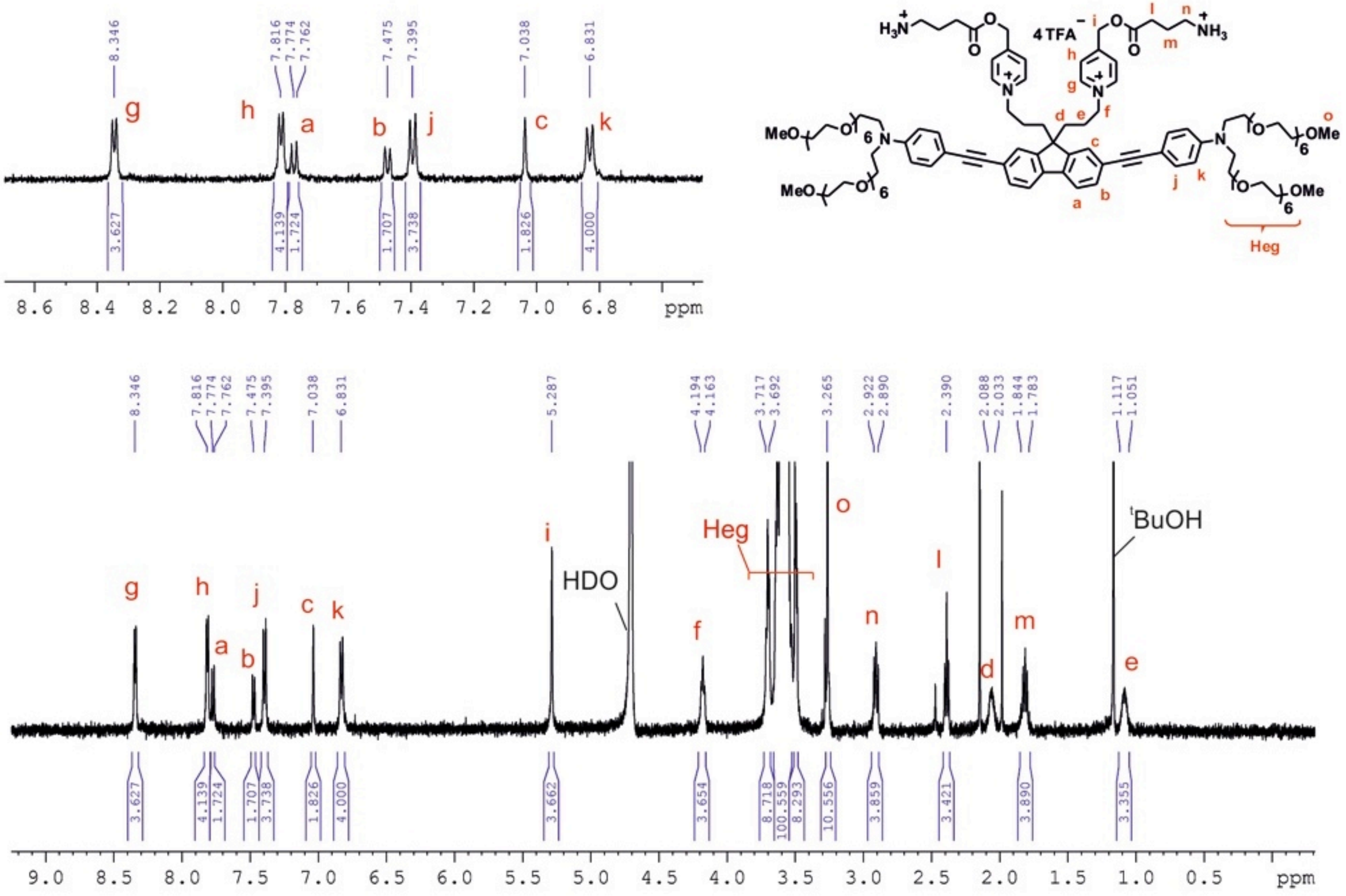

Figure S8. ^{1}H NMR spectrum of **BEF-Pyr-GABA** with zoom on the aromatic region (500 MHz, D_2O, 298 K).

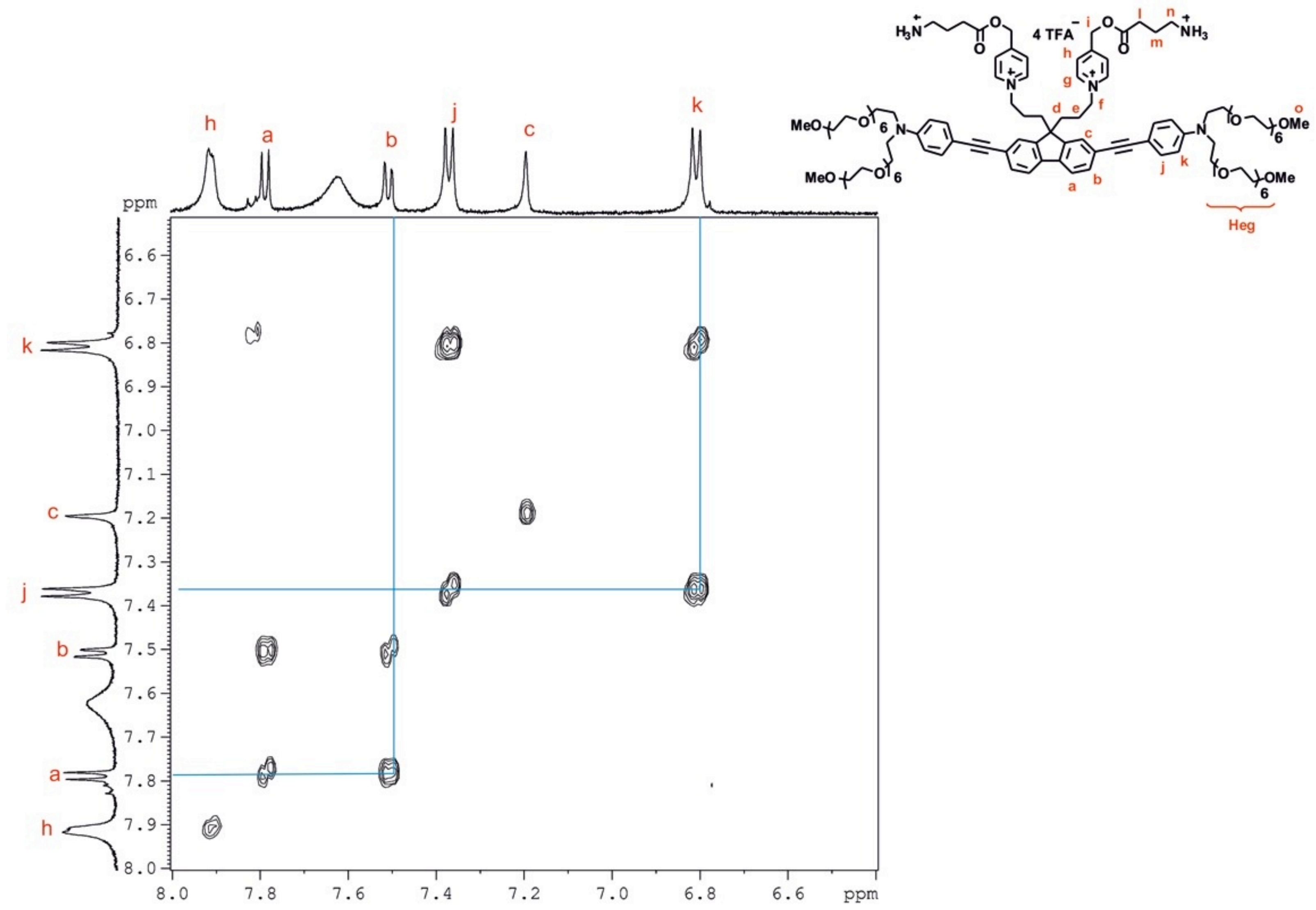

Figure S9. Part of the ^{1}H-^{1}H COSY spectrum showing coupling between the fluorene core protons (**a**, **b**) and aniline protons (**j**, **k**) in **BEF-Pyr-GABA** (500 MHz, $CD_3CN + D_2O$, 298 K).

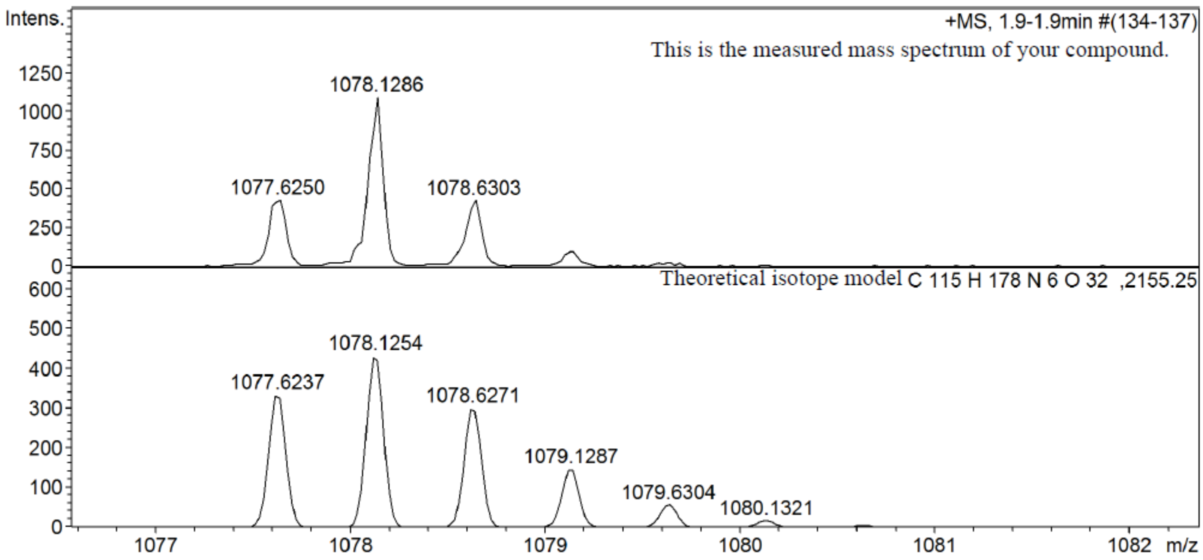

Figure S10.Positive ion electrospray mass spectra of **BEF-Pyr-GABA**, measured (*top*) and theoretical for $[C_{115}H_{178}N_6O_{32}]^{2+}$ (*bottom*).

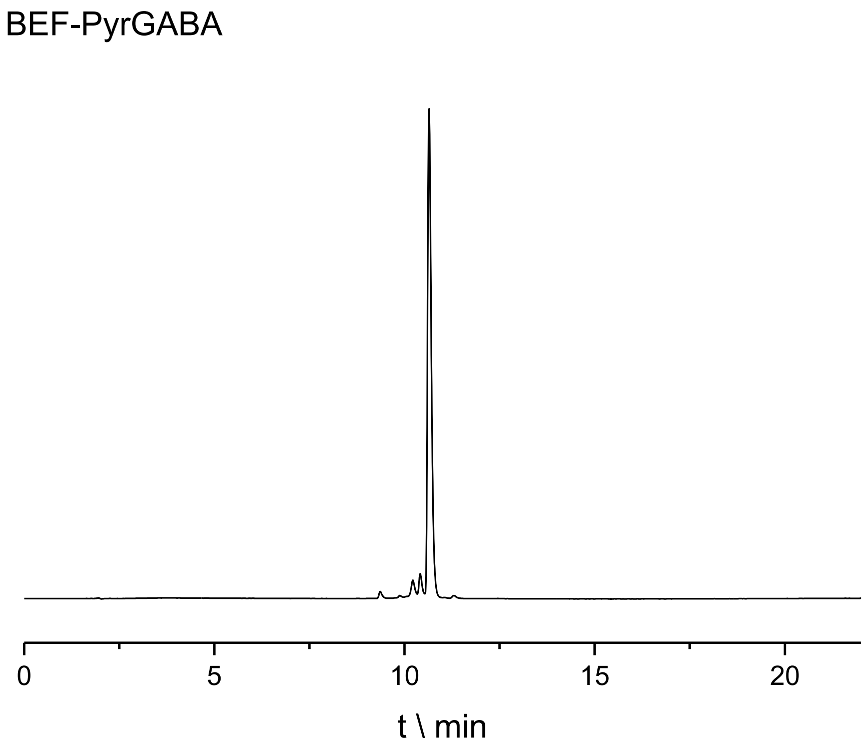

Figure S11.HPLC chromatogram of **BEF-Pyr-GABA**, absorption recorded at 375 nm, *HPLC method 3*. Analysis was performed with C18 5 µm, 4.6 × 150 mm Eclipse XDB-C18 column (Agilent).

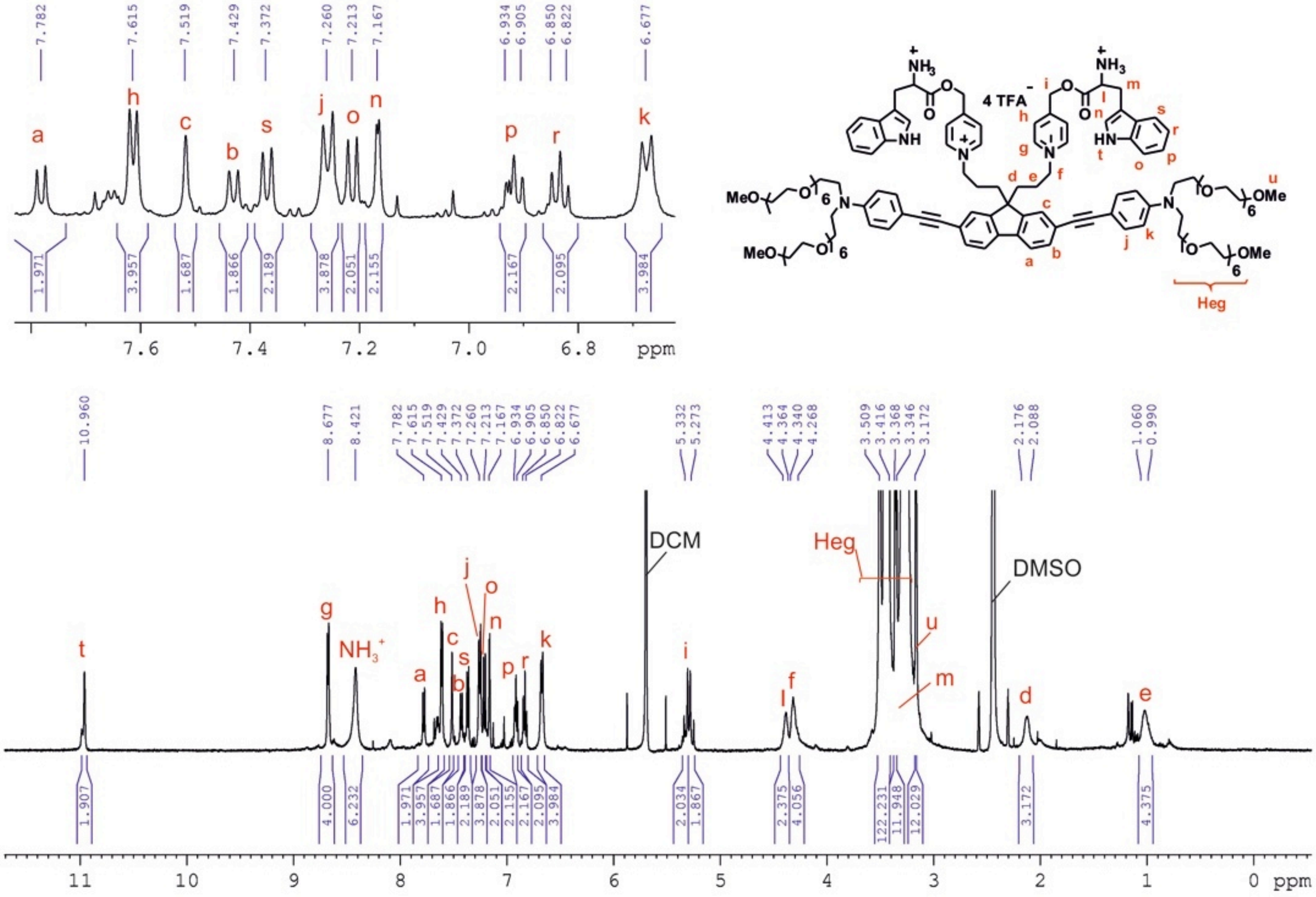

Figure S12. ^{1}H NMR spectrum of **BEF-Pyr-Trp** with zoom on the aromatic region (500 MHz, DMSO-d_6, 298 K).

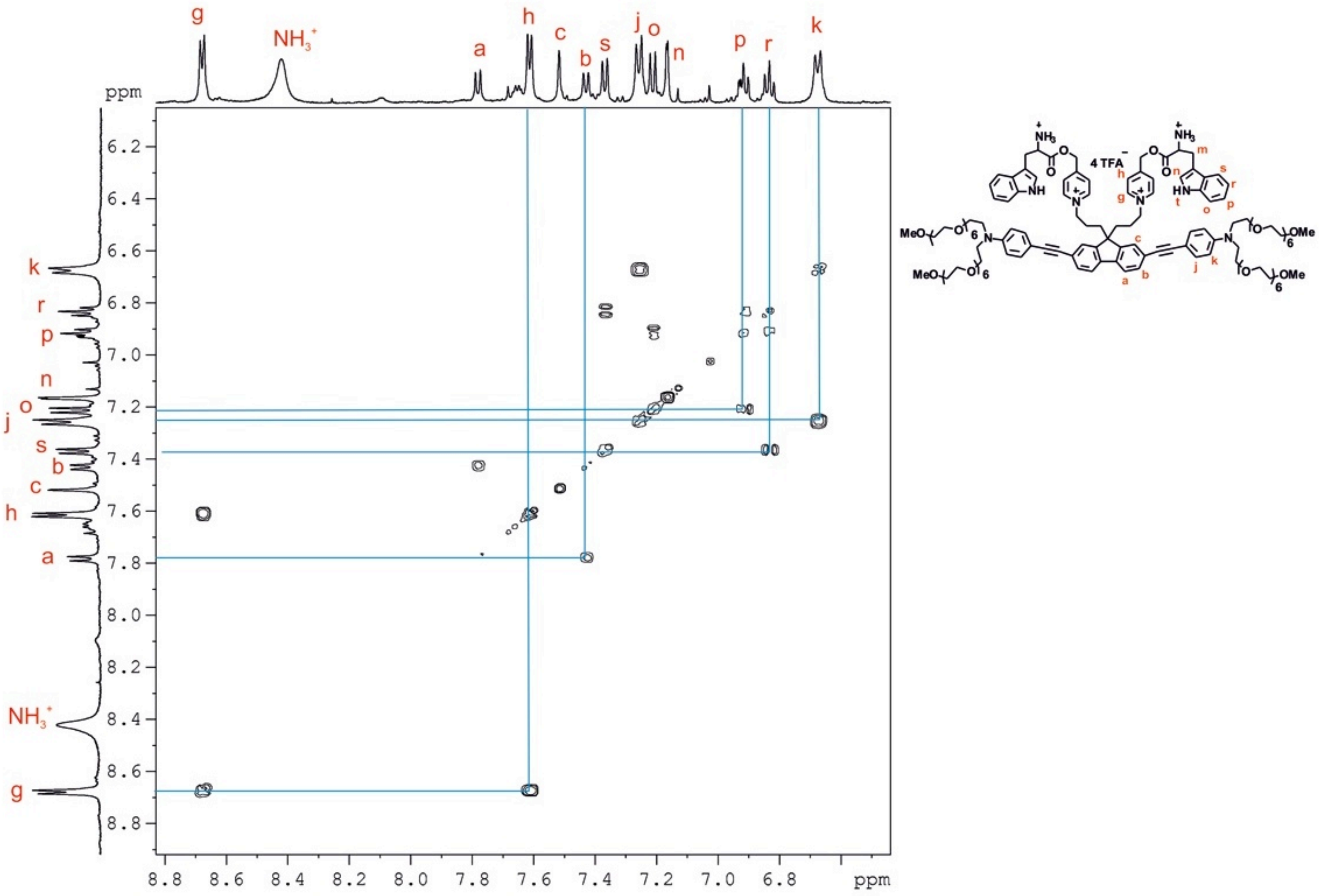

Figure S13. Part of the ^{1}H-^{1}H COSY spectrum showing coupling between the fluorene core protons (**a**, **b**), aniline protons (**j**, **k**), pyridinium protons (**g**, **h**) and tryptophan protons (**o**, **p**, **r**, **s**) in **BEF-Pyr-Trp** (500 MHz, DMSO-d_6, 298 K).

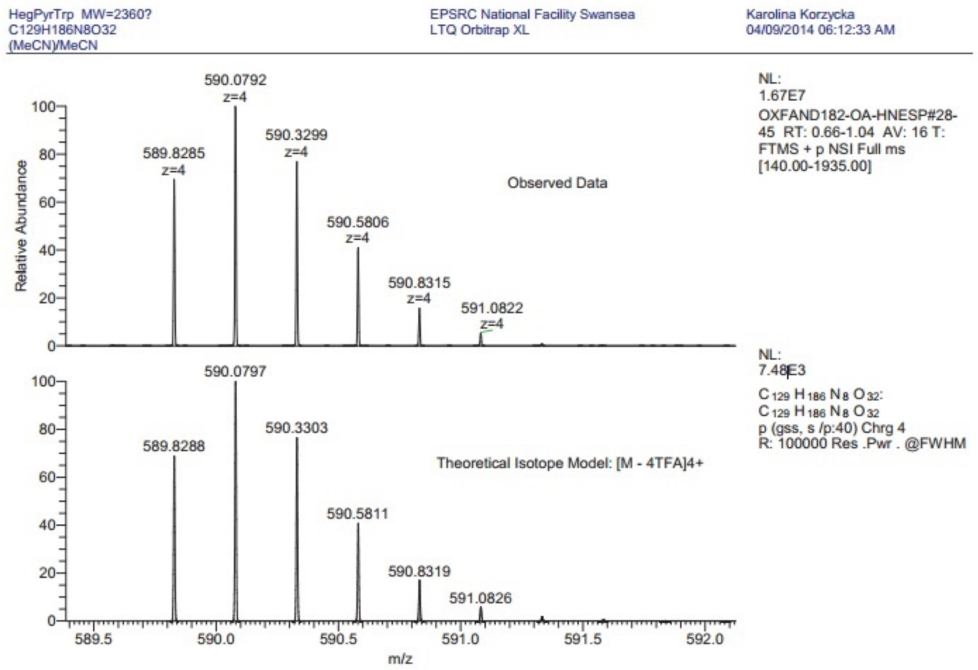

Figure S14. Positive ion electrospray mass spectra of **BEF-Pyr-Trp**, measured (*top*) and theoretical for $[C_{129}H_{186}N_8O_{32}]^{4+}$ (*bottom*).

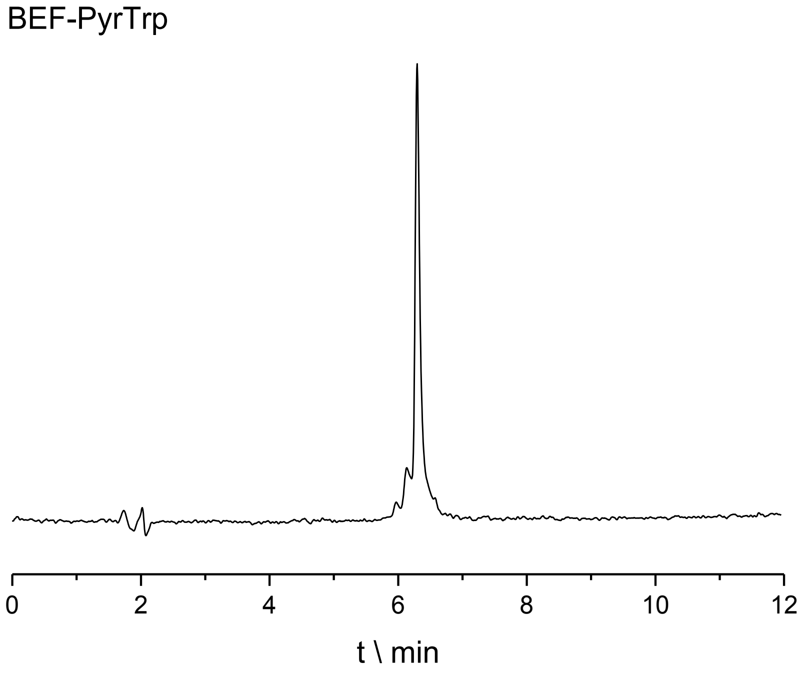

Figure S15. HPLC chromatogram of **BEF-Pyr-Trp**, absorption recorded at 375 nm, *HPLC method 1*.

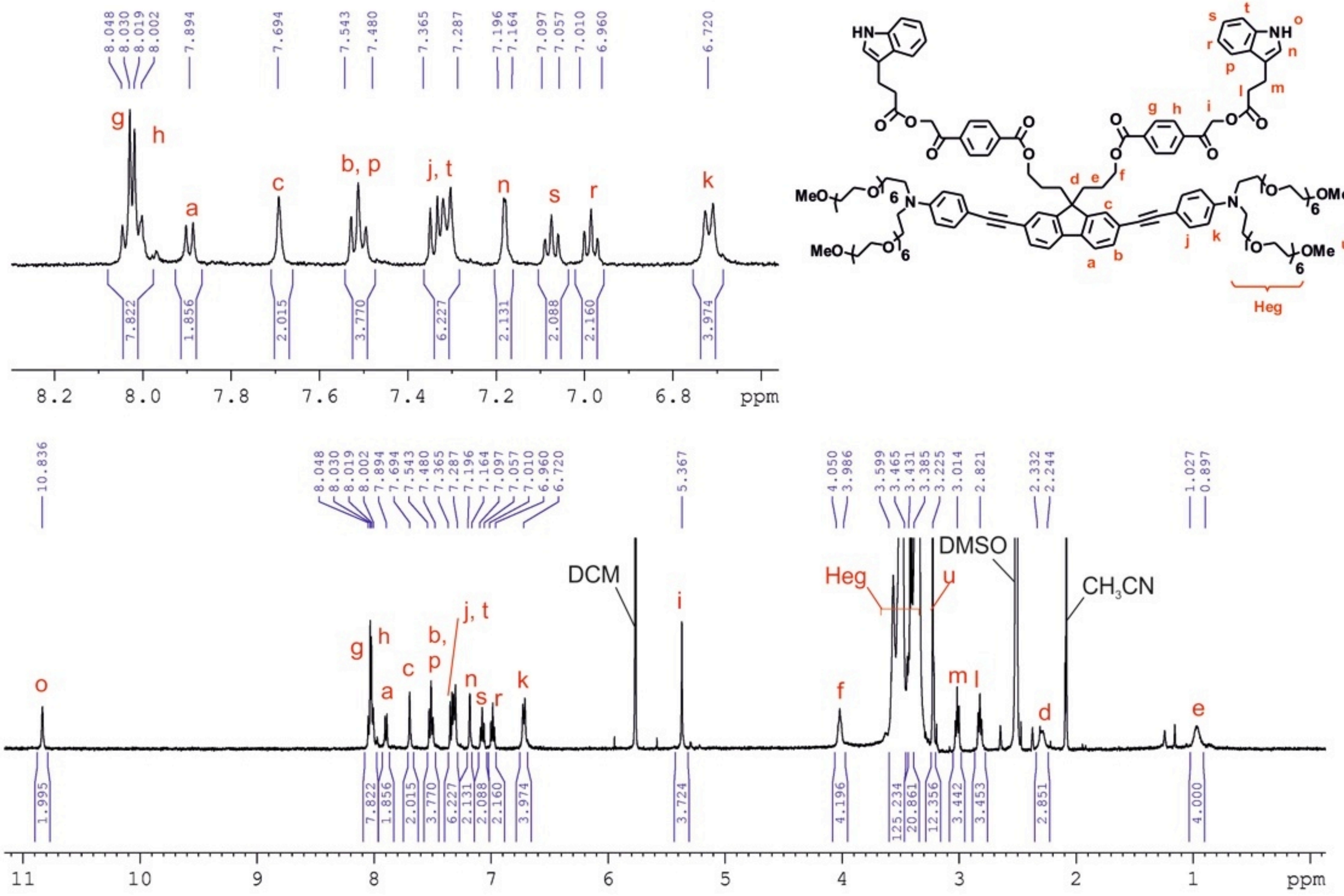

Figure S16. ^{1}H NMR spectrum of **BEF-Phen-Ind** with zoom on the aromatic region (500 MHz, DMSO-d_6, 298 K).

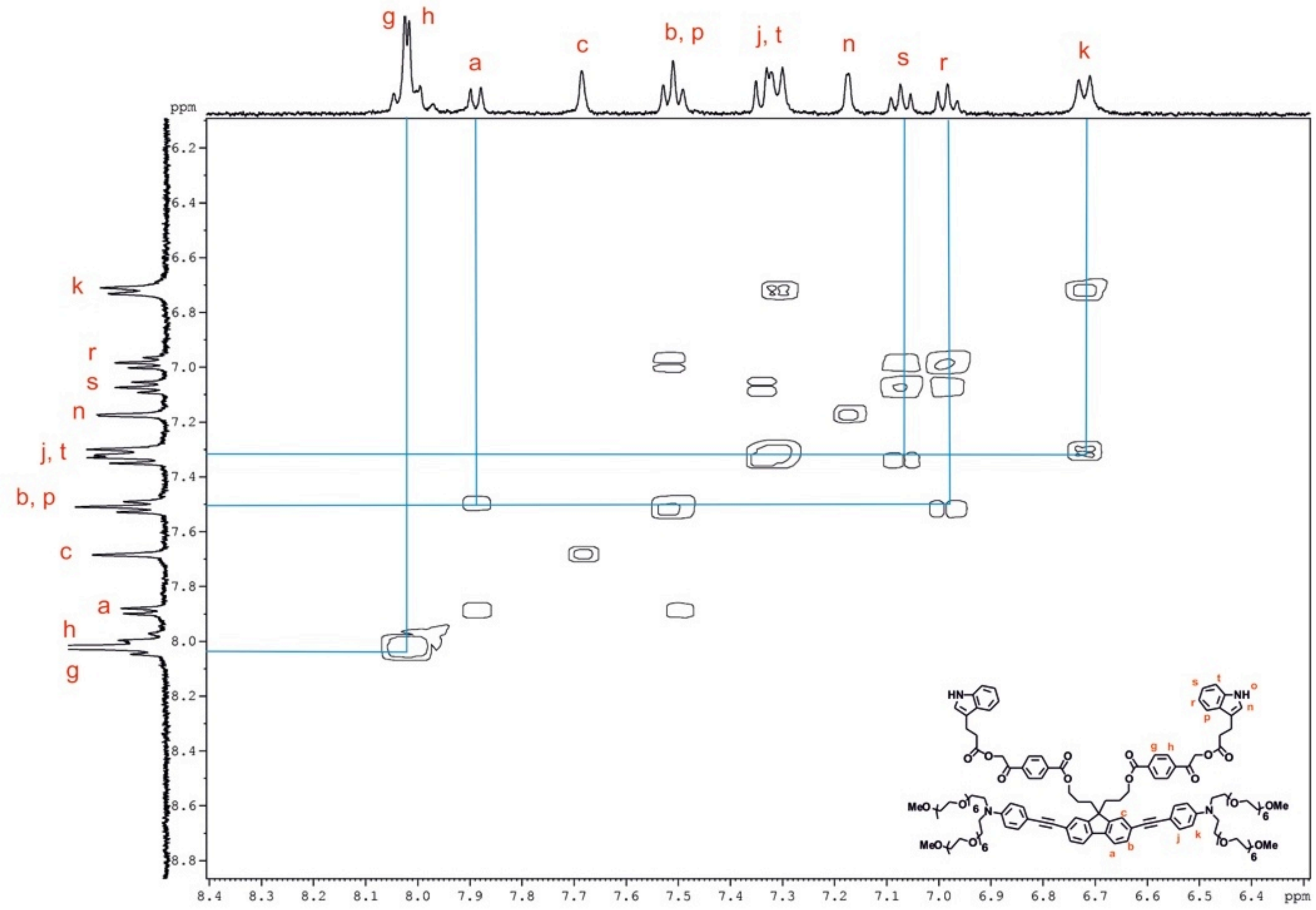

Figure S17. Part of the ^1H-^1H COSY spectrum showing coupling between the fluorene core protons (**a**, **b**), aniline protons (**j**, **k**), phenacyl protons (**g**, **h**) and 3-indolepropanoate protons (**p**, **r**, **s**, **t**) in **BEF-Phen-Ind** (500 MHz, DMSO-d_6, 298 K).

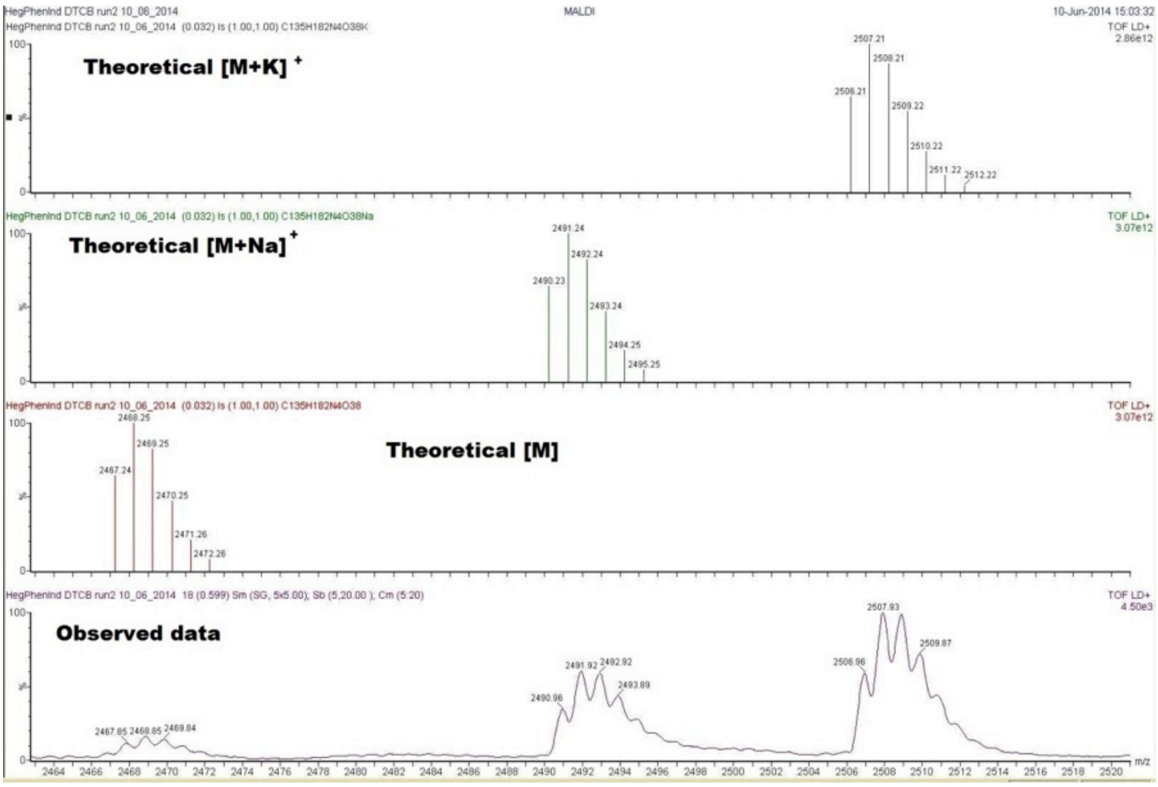

Figure S18. MALDI-TOF isotope patterning of [**BEF-Phen-Ind**], [**BEF-Phen-Ind**+Na]$^+$, [**BEF-Phen-Ind**+K]$^+$*(bottom)* and theoretical pattern calculated for $C_{135}H_{182}N_4O_{38}$, $C_{135}H_{182}N_4O_{38}Na^+$, $C_{135}H_{182}N_4O_{38}K^+$ *(top)*.

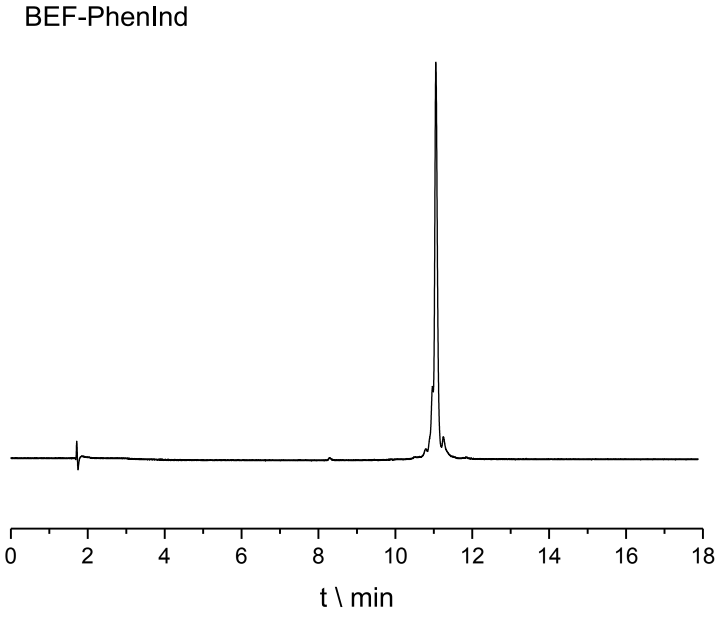

Figure S19.HPLC chromatogram of **BEF-Phen-Ind**, absorption recorded at 375 nm, *HPLC method 2*.

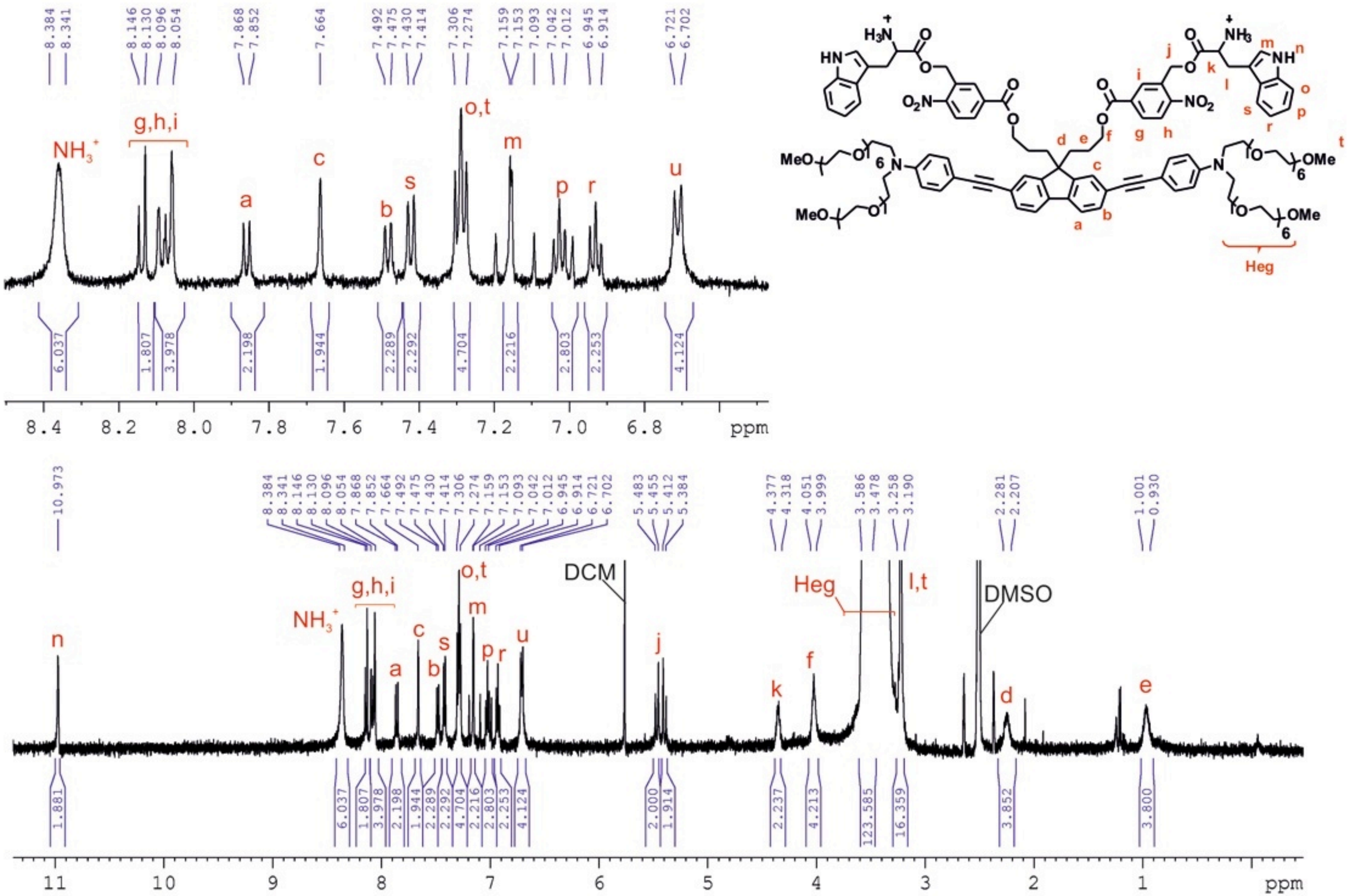

Figure S20. ^{1}H NMR spectrum of **BEF-NB-Trp** with zoom on the aromatic region (500 MHz, DMSO-d_6, 298 K).

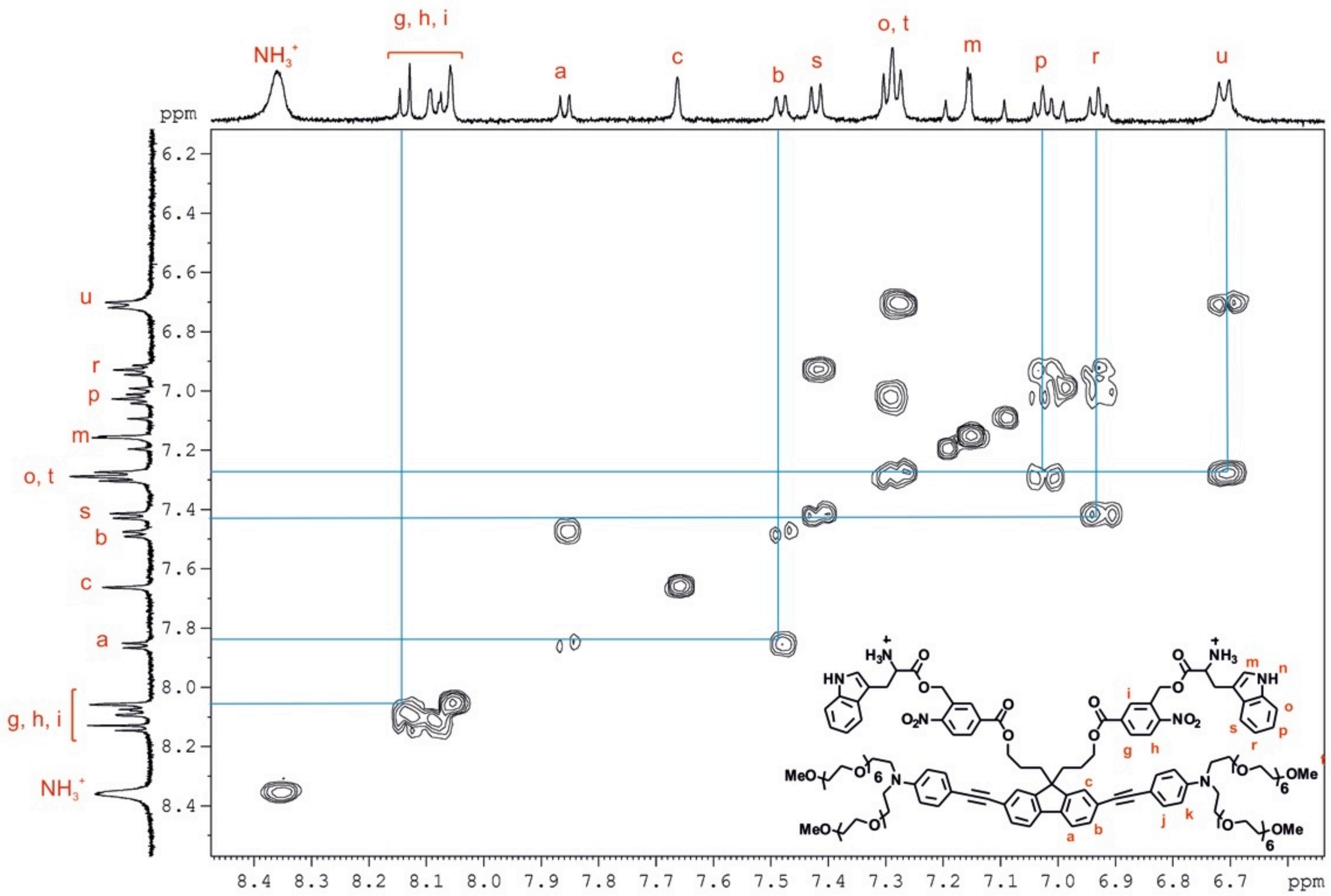

Figure S21. Part of the ^{1}H-^{1}H COSY spectrum showing coupling between the fluorene core protons (**a**, **b**), aniline protons (**j**, **k**), nitrobenzyl protons (**g**, **h, i**) and tryptophan protons (**o**, **p**, **r**, **s**) in **BEF-NB-Trp** (500 MHz, DMSO-d_6, 298 K).

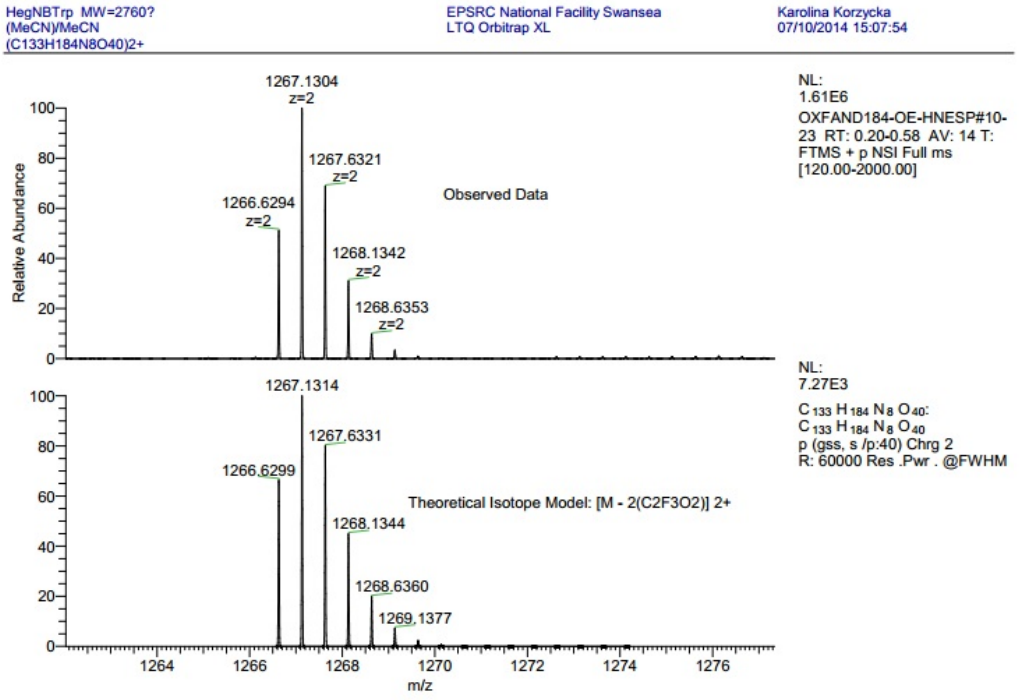

Figure S22. Positive ion electrospray mass spectra of **BEF-NB-Trp**, measured (*top*) and theoretical for $[C_{133}H_{184}N_8O_{40}]^{2+}$ (*bottom*).

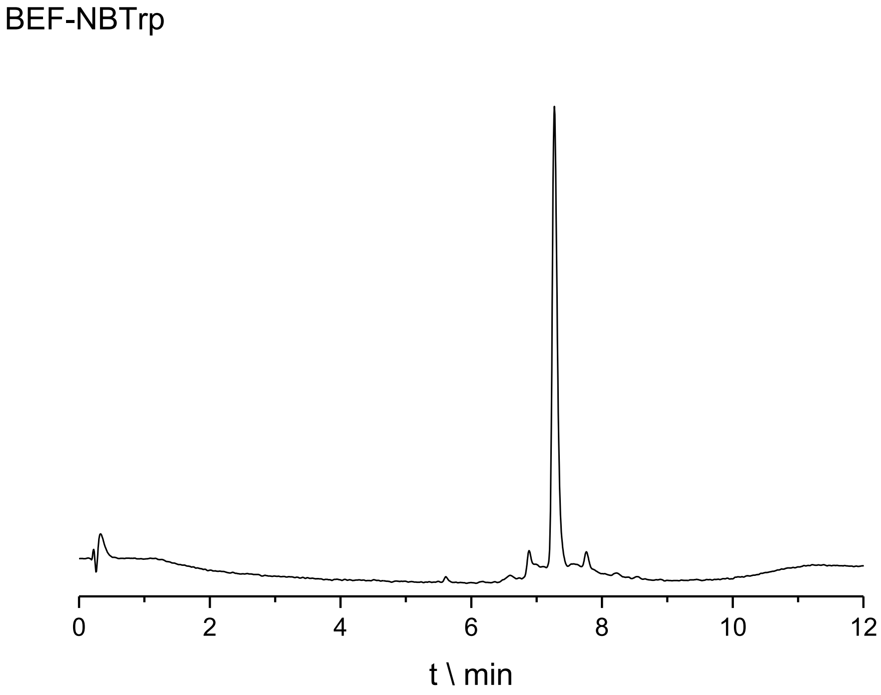

Figure S23. HPLC chromatogram of **BEF-NB-Trp**, absorption recorded at 375 nm, *HPLC method 1*. Analysis was performed with C18 5 µm, 2.1 × 50 mm Zorbax SB-C18 column (Agilent).

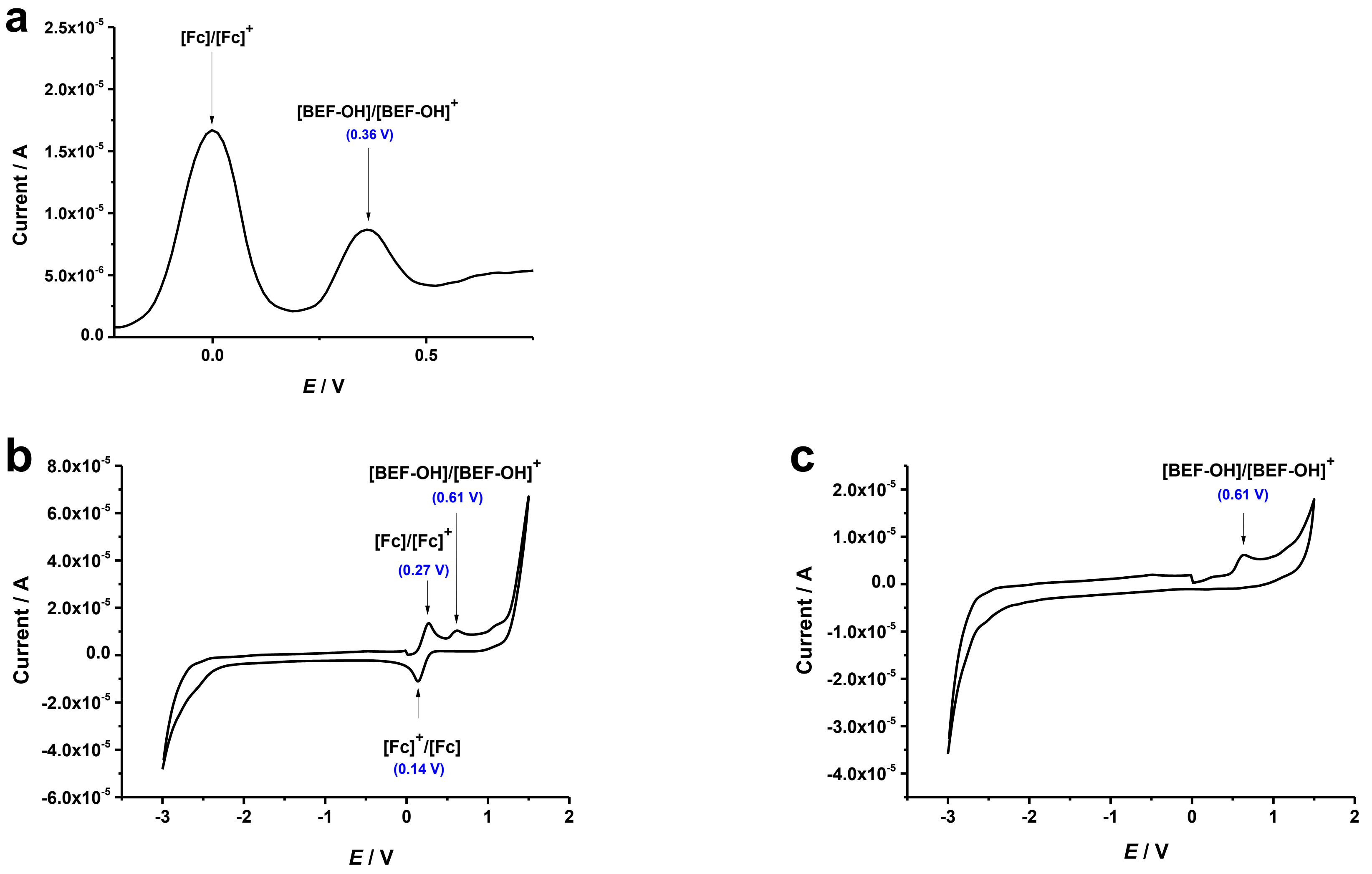

Figure S24. Determination of electrochemical properties of the model electron donor **BEF-OH**, **a)** square wave with ferrocene as internal standard; **b)** cyclic voltammetry with ferrocene as internal standard; **c)** cyclic voltammetry without standard. The results show that the first oxidation potential of **BEF-OH** is 0.36 V vs. Fc/Fc^+ (in THF, with 0.1 M Bu_4PF_6).

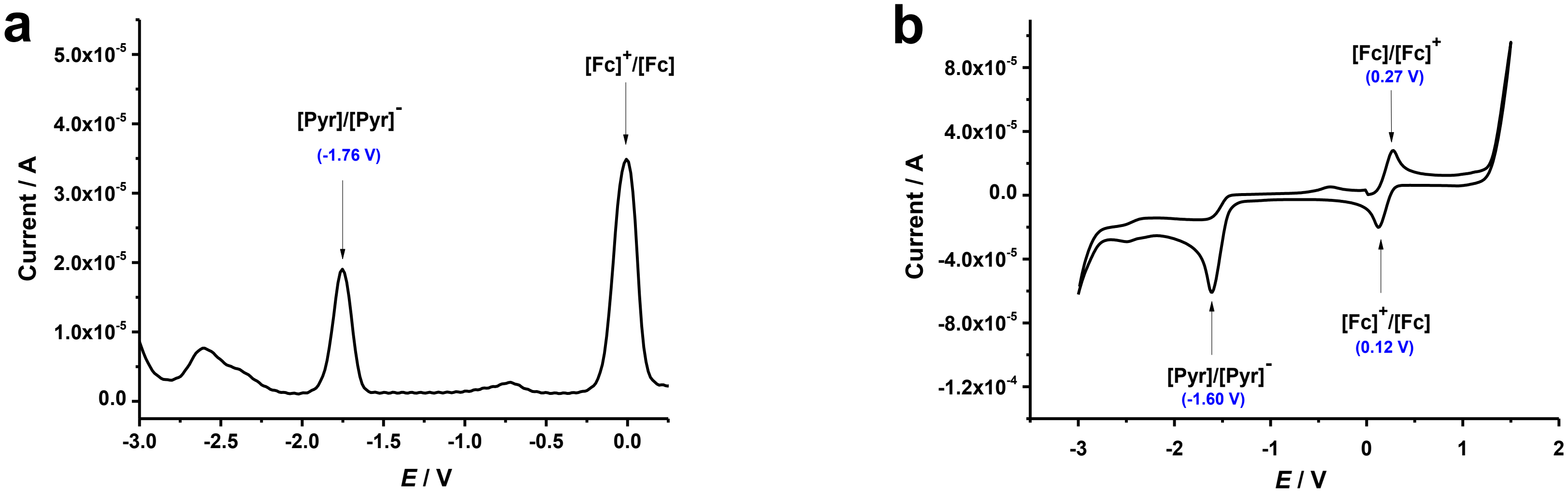

Figure S25. Determination of electrochemical properties of the model electron acceptor **Pyr** (*N*-methyl pyridinium hexafluorophosphate), **a)** square wave-reduction with ferrocene as standard; **b)** cyclic voltammetry.. The results show that the first reduction potential of **Pyr** is –1.76 V vs. Fc/Fc^+ (in THF, with 0.1 M Bu_4PF_6)

3.2. Absorption and emission spectra of model compounds

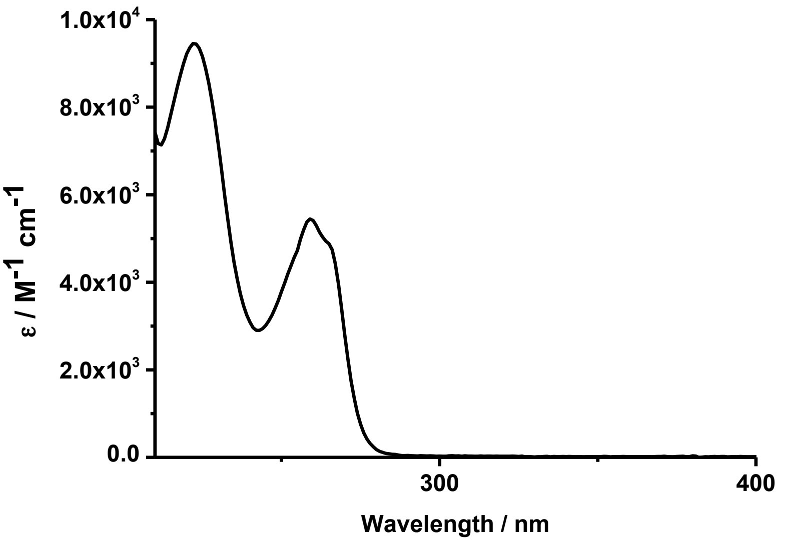

Figure S26. Absorption spectrum of model electron acceptor *N*-methyl pyridinium hexafluorophosphate (**Pyr**) at 293 K in THF.

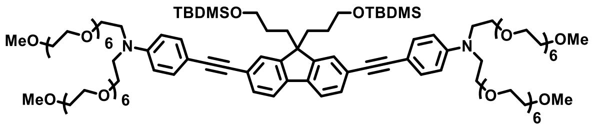

Figure S27. Structure of t-butyldimethylsilyl ether derivative **BEF-OTBDMS**. Synthesis and characterization data are reported in Section 1.2.1 and Section 2.

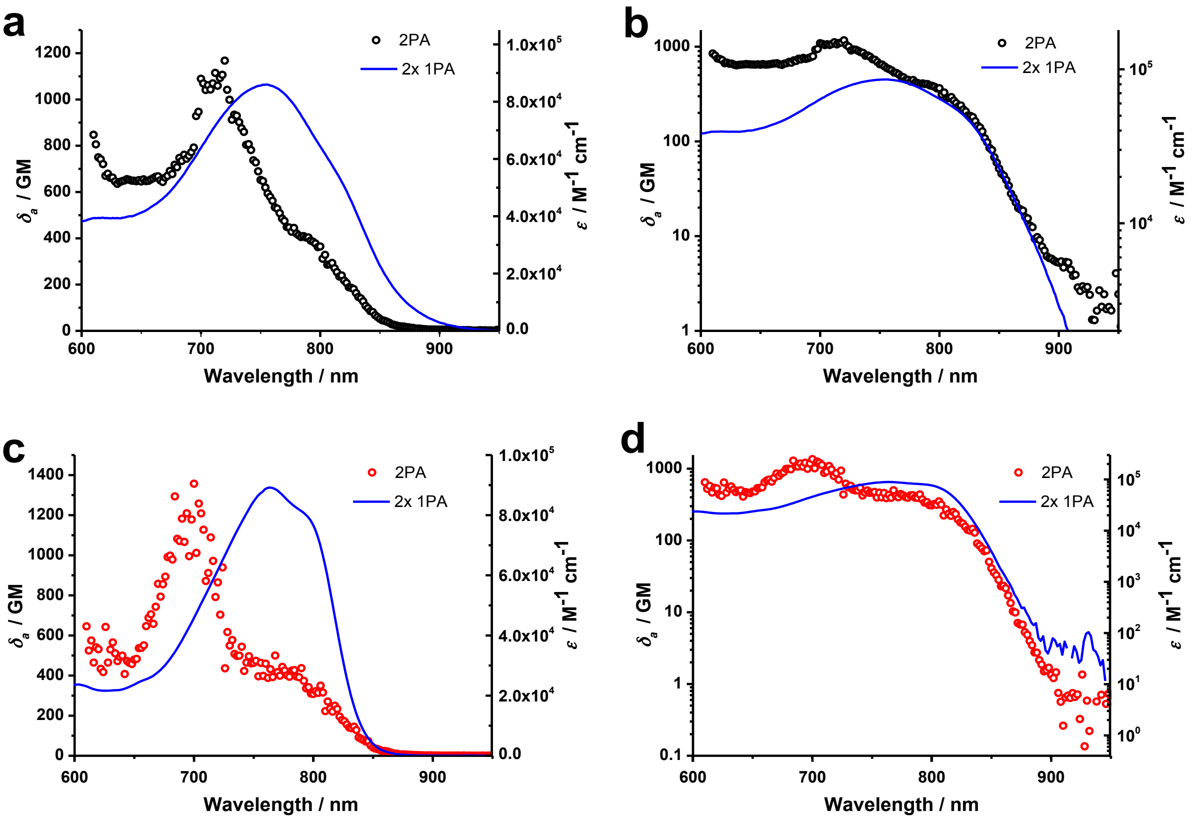

Figure S28. Two-photon absorption spectra (circles) overlaid with-wavelength-doubled one-photon absorption spectra (blue line); **a, b**) absorption spectra of **BEF-OH** in H_2O, **c, d)** spectra of **BEF-OTBDMS** in EtOH. Spectra **b** and **d** were drawn in the logarithmic scale. These 2PA spectra were measured using the standard two-photon excited fluorescence technique, as described in ref S20 (using the same conditions as those in ref S21, with the exception that fluorescein in pH 11 buffer solution was used for the reference compound).

4. One-photon photolysis

-4.1 One-photon uncaging of BEF-Pyr-GABA

The one-photon photolysis experiments were carried out by irradiating 1 mM solution of **BEF-Pyr-GABA** in D_2O in presence of 1 mM ^{t}BuOH as an internal reference for peak integration. Photolysis was performed using a Rayonet RMR-600 (300–400 nm, with peak at 350 nm) and Philips CLEO 15 W lamp (UV type 3) to irradiate vigorously stirred solutions in Wilmad 5 mm thin wall 7” 300 MHz NMR tubes (**Figure S29**). Samples were photolyzed for ~1 h with time intervals every ~5–10 min to monitor progress of the reaction and record NMR spectra (Bruker AVII 500 MHz). At time intervals during the irradiation, the lamps were switched off and the stirrer bar removed. Following each measurement, irradiation and stirring were restarted. During transfer to and from the instrument, the samples were shielded from light. After photolysis was completed the presence of free GABA was confirmed by doping the irradiated solution with commercially available GABA. The three multiplets of the suspected photoreleased GABA increased in intensity and no new signals were observed (**Figure S30**).

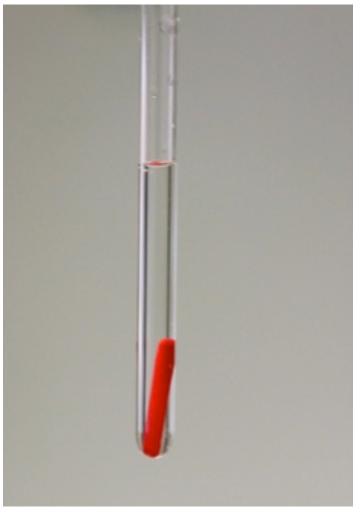

Figure S29. A magnetic stirrer bar (red) in D_2O (0.5 mL) in an NMR tube. By placing the photolysis chamber on a magnetic stirrer, a satisfactory vortex could be created in the solution.

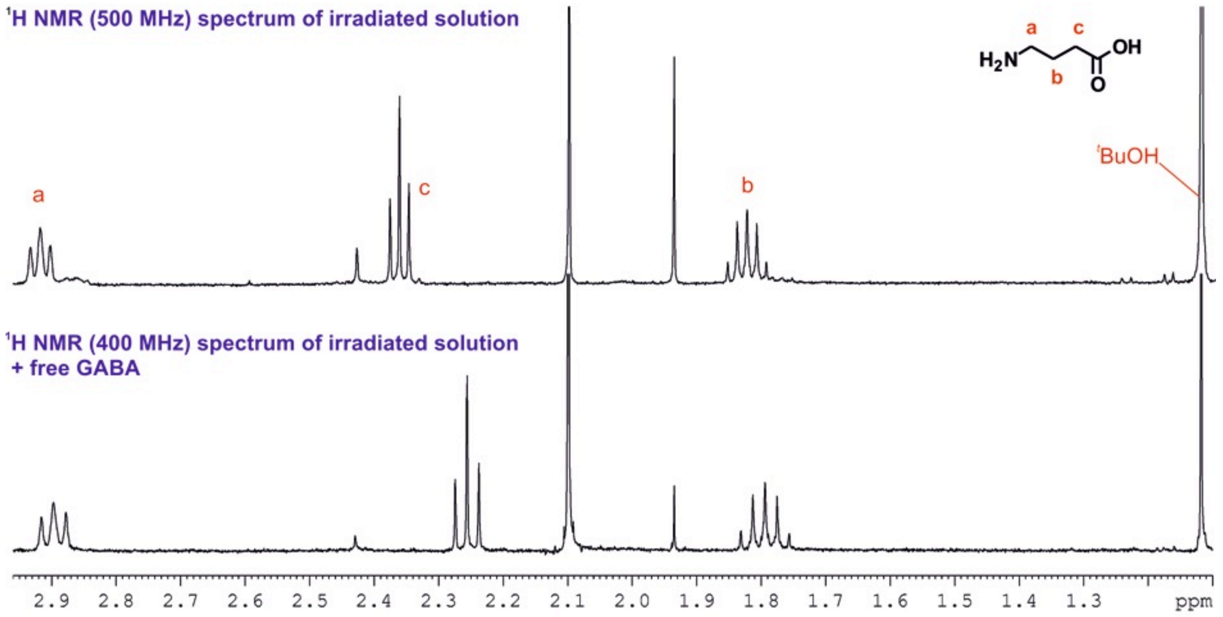

Figure S30. A ^{1}H NMR spectrum (400 MHz, D_2O) of a completed uncaging reaction (*top*) of **BEF-Pyr-GABA** and after the addition of one molar equivalent of GABA (500 MHz, D_2O) (*bottom*). The three multiplets of the suspected photoreleased GABA approximately doubled in integration and no new peaks were observed in the region. The small shift in signal position is likely due to the differences in field strength and concentration between the two spectra.

To take account of hydrolysis of **BEF-Pyr-GABA** a control experiment was carried by dissolving 1 mM **BEF-Pyr-GABA** in D_2O and recording NMR spectra at 20°C. The stability of the probe was monitored for 1.5 h. No hydrolysis was observed within the experiment time (**Figure S31**). No new peak were formed over time as well as the integration of GABA signals (**n**, **l, m**) relative to alkyl chain CH_2 signal (**d**) remained unchanged.

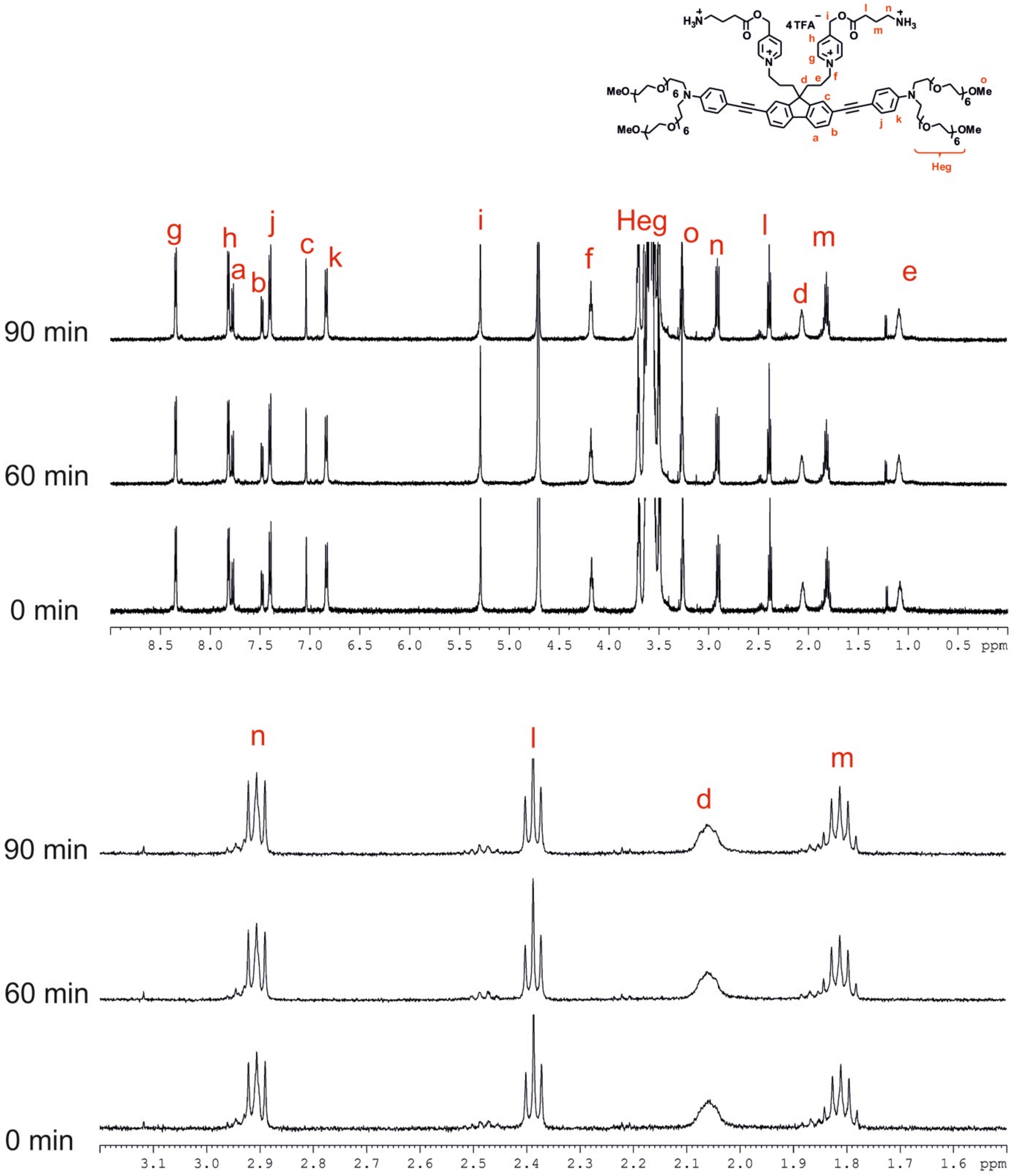

Figure S31. Stability of **BEF-Pyr-GABA** in D_2O at 20°C over 1.5 h. *Top* – full range NMR spectra, *bottom* – zoom on aliphatic region showing GABA peaks. Integration of peaks **n**, **l** and **m** relative to peak **d** remained constant.

4.2 The determination of the quantum yield of uncaging

4.2.1 Comparison with the DPNI-GABA as a standard

The quantum yield of uncaging ϕ_u of **BEF-Pyr-GABA** was determined by simultaneous irradiation of **BEF-Pyr-GABA** and commercially available DPNI-GABA (1 mM, D_2O), for which $\phi_{u\,(DPNI-GABA)} = 0.085$.[S22] Progress of uncaging was monitored by ^{1}H NMR. Integrals of the signals corresponding to caged GABA were used to construct [GABA] and [caged-GABA] versus time plots for each compound, which followed first order reaction kinetics (**Figure S32**). ^{t}BuOH was used as an internal reference for peak integration.

The rates of uncaging (DPNI-GABA 5.1 × 10^{-3} s^{-1} and **BEF-Pyr-GABA** 5.2 × 10^{-4} s^{-1}) were used to solve **Equation S1** giving a quantum yield of uncaging of 0.009 ± 0.003.

$$\phi_{u\,(BEF-Pyr-GABA)} = 0.085\,\left(\frac{k_{u(\mathbf{BEF-Pyr-GABA})}}{k_{u(DPNI-GABA)}}\right) \qquad \textbf{Equation S1}$$

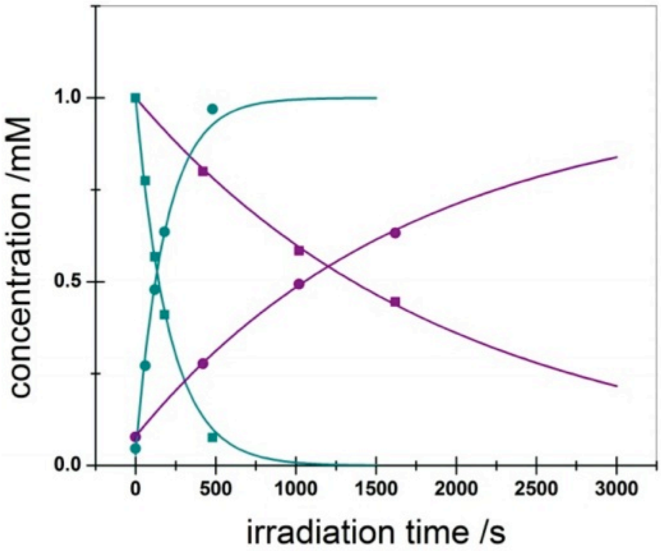

Figure S32. The concentration of caged GABA over time during simultaneous irradiation of DPNI-GABA (cyan) and **BEF-Pyr-GABA** (purple). Gradients of caged-GABA decay were 4.8 × 10^{-3} s^{-1} for DPNI-GABA (cyan, squares) and 5.1 × 10^{-4} s^{-1} for **BEF-Pyr-GABA** (purple, squares), while gradients for GABA uncaging were 5.4 × 10^{-3} s^{-1} for DPNI-GABA (cyan, circles) and 5.4 × 10^{-4} s^{-1} for **BEF-Pyr-GABA** (purple, circles). The rate of uncaging (k_u) was defined as the average of the two gradients.

4.2.2 Actinometry

To verify the value of one-photon uncaging quantum yield obtained by the comparison to the standard, we employed an alternative method based on the actinometry. **Equation S2** was used in calculations, as quantum yield of uncaging ϕ_u is defined as a ratio of number of molecules uncaged to the number of photons absorbed by the irradiated solution.[S23]

$$\phi_u = \frac{kcVN_A}{f(1-10^{-A})} \quad \textbf{Equation S2}$$

Equation S2. Equation used in calculating one-photon uncaging quantum yield ϕ_u where k - GABA release rate constant, c - concentration of the photolysed solution, V- volume of the photolysed solution, N_A – Avogadro number, f - number of photons administrated, A - absorption of the irradiated solution at the photolysis wavelength.

The number of molecules released per second, $kcVN_A$, was determined by following the progress of the reaction by ^{1}H NMR. The number of photons absorbed by the solution was calculated considering the number of administrated photons f and absorbance A of the sample. The photon flux of the light source was measured with ferric oxalate actinometry.

Ferric oxalate actinometry protocol[S24]

All procedures were carried out in the dark. Ferric oxalate solution (0.5 mL, 0.006 M, prepared by dissolving 300 mg of potassium ferric oxalate trihydrate ($K_3[Fe(C_2O_4)_3]\cdot 3H_2O$) in 100 mL of 0.05 M H_2SO_4) was placed in a quartz cuvette and irradiated (with the Rayonet RMR-600 for 10 s, or with the Philips CLEO 15 W lamp for 5 s) while an identical control sample was kept in the dark. Irradiated ferric oxalate solution (0.2 mL) and a control sample (0.2 mL) were transferred to the cuvettes containing a mixture of buffered 0.1% phenanthroline solution (0.9 mL, prepared by dissolving 22.5 g of $CH_3CO_2Na\cdot 3H_2O$ and 0.100 g of phenanthroline in 100 mL of 0.5 M H_2SO_4) and H_2O (0.9 mL). Samples were kept in the dark for 1 h to allow the complexation to occur and their absorbance at 510 nm was measured. The irradiation time was chosen to ensure that the photoconversion of ferric oxalate does not exceed 5% and that the absorption of the $Fe(phen)_3^{2+}$ complex is within 0.5–1.

Upon exposure to light potassium ferric oxalate ($K_3[Fe(C_2O_4)_3]$) is converted to Fe^{2+} and as the quantum yield ϕ_R of formation of Fe^{2+} is well-known for a range of wavelengths (222–500 nm) the photon flux f irradiating the solution can be calculated with use of **Equation S3**,

$$f = \frac{no.\ moles Fe^{2+}}{\phi_R t(1-10^{-A})} \quad \textbf{Equation S3}$$

where ϕ_R is the quantum yield of the ferric oxalate conversion at the irradiation wavelength, t (s) is the irradiation time, $(1-10^{-A})$ is the fraction of light absorbed by the ferric oxalate solution while A is the absorption of 0.006 M solution at the irradiation wavelength. Formation of the colored $Fe(phen)_3^{2+}$complex (ε_{510} = 11,100 M^{-1} cm^{-1}) allows to quantify amount of Fe^{2+} generated during the irradiation by measuring the absorption of irradiated and non-irradiated sample (**Equation S4**).[S25]

$$no.molesFe^{2+} = \frac{V_1 V_3 \Delta A(510\ nm)}{10^3 V_2 l\ \ (510\ nm)}$$ **Equation S4**

Where V_1 (mL) is the irradiated volume, V_2 (mL) is the aliquot of the irradiated solution transferred to the solution of buffered phenanthroline, V_3 (mL) is the final volume of the complexation solution, l (cm) is the optical path length of the quartz cuvette used for irradiation, ΔA (510 nm) is the difference in absorbance between the irradiated solution and control stored in the dark, and ε (510 nm) is the extinction coefficient of the complex ($Fe(phen)_3^{2+}$) at 510 nm.

The calculated value of the photon flux was expressed in $E \cdot s^{-1}$ and was converted to mols of photons$\cdot s^{-1}$ by multiplying the obtained value with the Avogadro number. The photon flux determined for Rayonet RMR-600 and Philips CLEO 15 W lamp were almost identical and on average of 1.9×10^{16} moles of photons$\cdot s^{-1}$.

The uncaging quantum yield for DPNI-GABA was verified by ferric oxalate actinometry and determined to be 0.088 ± 0.004 (in close accordance with the published value), while the quantum yield for **BEF-Pyr-GABA** 0.009 ± 0.004.

4.3 One-photon photolysis of BEF-Pyr-Trp.

4.3.1 Wavelength dependent uncaging experiments.

One-photon photolysis of **BEF-Pyr-Trp** was carried out with a broad source of light Rayonet RMR-600 (300–400 nm, with peak at 350 nm), while wavelength dependent uncaging experiments were performed with use of and JobinYvon Ltd. Fluoromax2 spectrometer (340 nm, 360 nm, 380 nm, 400 nm, 420 nm, with 5 nm band width).

Fluorescence cuvette (10 mm path length) was filled with 3 mL of citric acid/ sodium citrate buffer (pH 3.0) and150 µL of the buffer were removed and replaced with 150 µL of the **BEF-Pyr-Trp** stock solution in H_2O +0.1% TFA/acetonitrile, (3:2, v/v) (due to the susceptibility of **BEF-PyrTrp** to decomposition we used fractions collected upon semi-preparative HPLC purification as stock solutions and stored them at –80 °C). The concentration (1.35–1.75 µM) was set such that A = 0.15–0.18 (for sample of 90% purity by HPLC and ε_{380} = 90 000 M^{-1} cm^{-1}). After measuring the absorbance the sample was split into 3 batches: *1* - 400 µL, which was photolyzed immediately at 350 nm in Rayonet (10 min); *2* - 400 µL, which was kept in dark and used as control sample, *3* - 2200 µL, which was placed in the fluorometer and photolyzed at one of the following wavelengths: 340 nm, 360 nm, 380 nm, 400 nm, 420 nm. 35 µL aliquots of the control and photolyzed solution were removed after following time: 0, 150 s, 300 s, 600 s, 900 s, 1200 s, 1800 s, 2800 s, 3800 s, placed in HPLC vials and stored in the liquid nitrogen then analyzed one by one by *HPLC method 1.*

Release of tryptophan was monitored and quantified at 220 nm, while depletion of starting material **BEF-Pyr-Trp** was quantified at 375 nm. HPLC chromatograms were transferred to Origin and integrated. Amount of released tryptophan was quantified with reference to the calibration curve, which was prepared for concentrations 1.24–6.20 µM (**Figure S33**) with use of *HPLC method 1.*

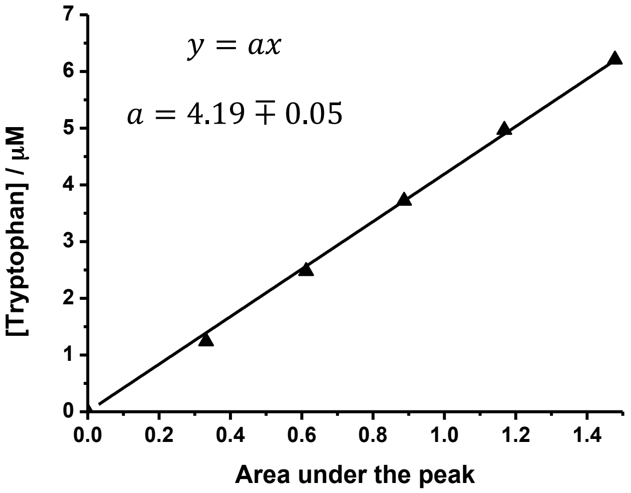

Figure S33. Tryptophan calibration curve prepared for 1.24–6.20 μM concentration.

The concentration of tryptophan released exclusively upon uncaging (net) was obtained upon subtraction of tryptophan concentration in control samples from tryptophan concentration in photolysis experiments. Plots for all uncaging experiments are presented in **Figure S34**. Chemical yield of uncaging for each experiment is presented in **Table S2**.

Table S2. Yield of uncaging obtained upon 3800 s of photolysis of 1.35–1.75 μM **BEF-Pyr-Trp** solutions in citric acid/ citrate buffer.

Wavelength / nm	Tryptophan concentration – measured / μM	Tryptophan concentration – expected / μM	Chemical yield of uncaging / %
340	1.58	3.00	52
360	2.33	3.54	65
380	2.15	2.4	89
400	1.77	2.4	73
420	1.3	3.54	36

The chemical yield of uncaging was ~85% when complete photolysis of **BEF-Pyr-Trp** was carried out with Rayonet RMR-600 (300–400 nm, 350 nm peak).

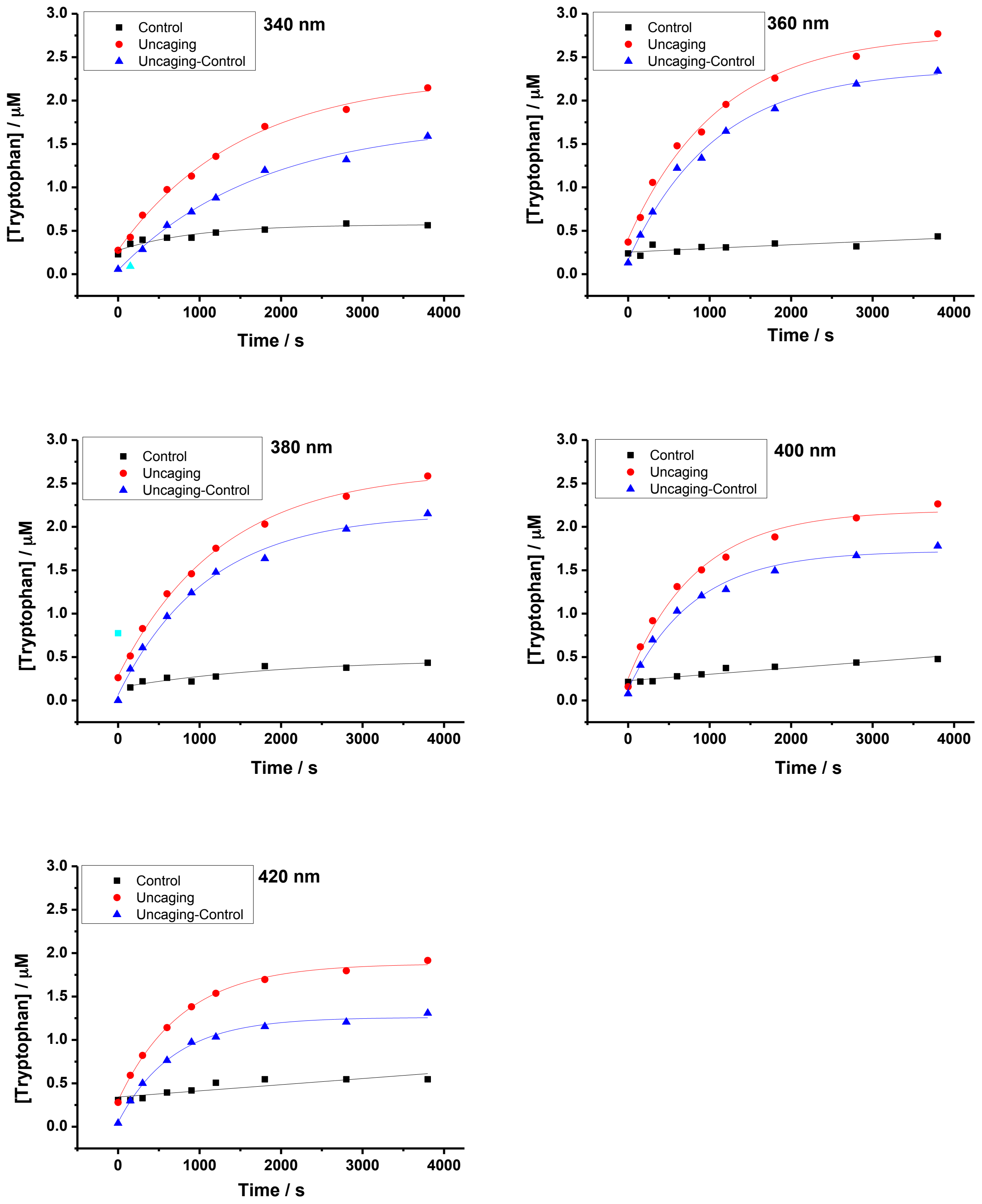

Figure S34. Photolysis experiments with **BEF-Pyr-Trp** carried out at 340 nm, 360 nm, 380 nm, 400 nm, 420 nm. Tryptophan concentration was determined by HPLC. Data have been fitted to first-order exponential curves.

4.3.2 Quantum yield of uncaging of BEF-Pyr-Trp

The uncaging quantum yield of **BEF-Pyr-Trp** was measured at 360 nm in citric acid/ citrate buffer (pH 3.0). Photolysis was carried out according to the protocol described in Section 4.3.1. Release of tryptophan followed the first order reaction kinetics and the rate of uncaging was determined as 9.3×10^{-4} s^{-1} (**Figure S35**). The photon flux of fluorometer was measured with ferric oxalate actinometry and was 3.30×10^{15} photons s^{-1}. The uncaging quantum yield was calculated as 0.0025.

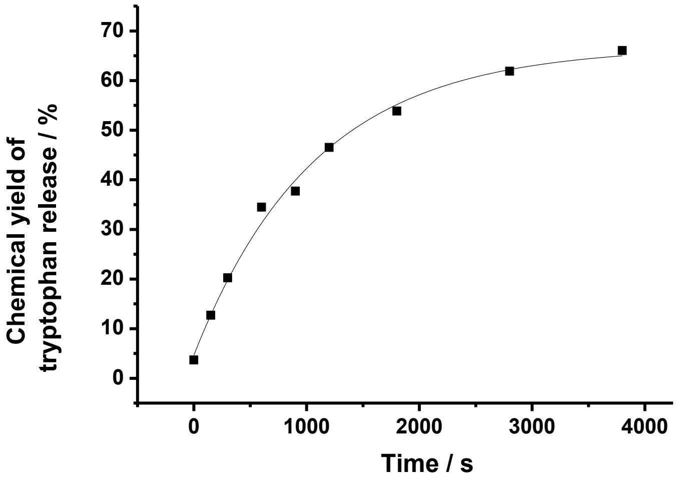

Figure S35. Photolysis of 1.77 µM solution of **BEF-Pyr-Trp** in citric acid/citrate buffer – increase in concentration of photoreleased tryptophan.

5. Stability experiments

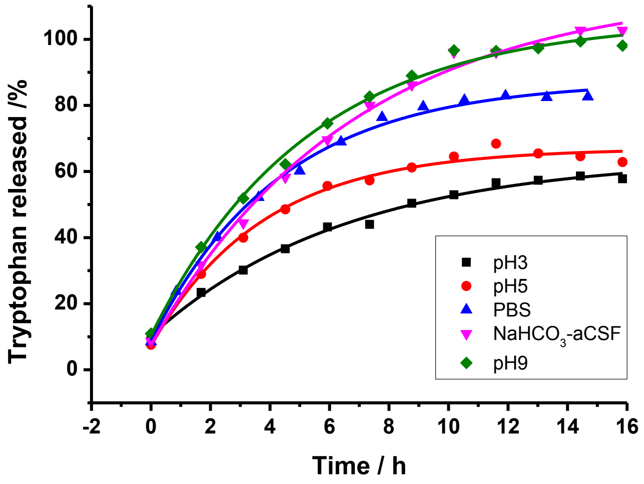

Figure S36. Stability studies of **BEF-Pyr-Trp**; release of tryptophan over time at pH 3–9. Data have been fitted to the first order exponential curves. Concentration of tryptophan was determined with HPLC.

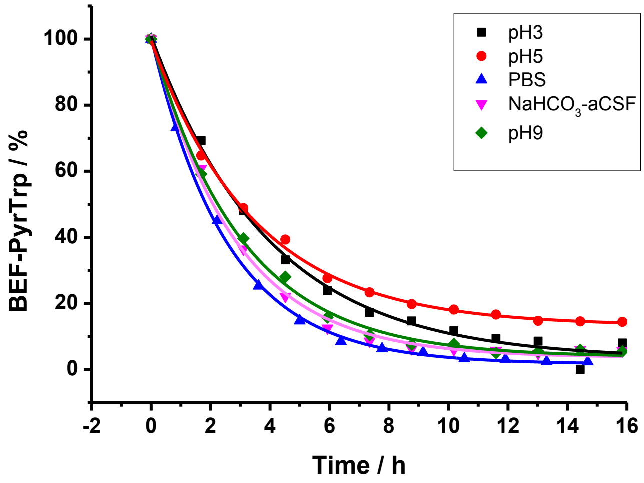

Figure S37. Stability studies of **BEF-Pyr-Trp**; depletion of the starting material over time at pH 3–9. Data have been fitted to the first order exponential curves. Concentration of **BEF-Pyr-Trp** was determined with HPLC.

7. Thermodynamics of electron transfer in non-pyridinium design

Free Gibbs energy for photoinduced electron transfer (ΔG_{ET}) for **Phen** and **NB** derivatives was calculated with consideration of interaction between charges, as expressed by Coulombic term (Equation 1 and 2). The distance between separated charges *a* was estimated as a center-to-center distance between the fluorene unit and release platform (center of each unit is represented by green ball, **Figure S38** and **Figure S39**). In the considered model structures, the electron donor and acceptor were separated by C-3 alkyl chain linker. To simplify calculations model compounds bore methyl substituents on aniline nitrogen atoms and release platforms bore acetic acid esters. Molecular mechanics and calculations were performed in HyperChem 8 using the MM+ forcefield. Non-bonded interactions were attenuated to zero by a switched potential between 4 Å and 2 Å. Minimizations were performed using the steepest descent algorithm. The distance between donor and acceptor was 8.7 Å for **NB** and 9.5 Å for **Phen**. Calculated term *w*(DA) was −0.22 eV for **NB** and −0.20 for **Phen**. The obtained value of the Coulombic term is an approximation as the distance measured between the donor and acceptor varies across possible conformations and introduces high uncertainty.

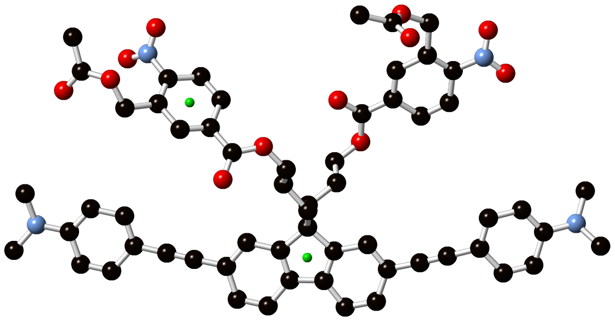

Figure S38. Model structure for calculation of the distance between the fluorene and **NB** release unit. Centers of the fluorene-dye and release unit are represented by green balls.

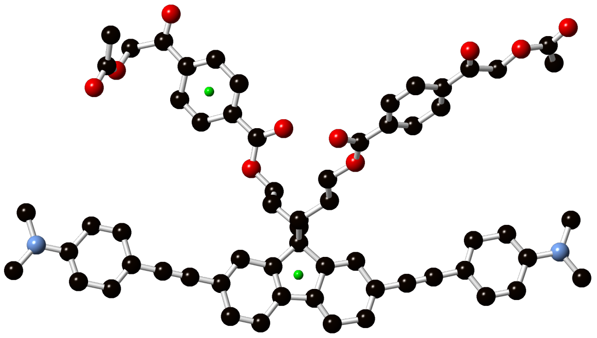

Figure S39. Model structure for calculation of the distance between the fluorene and **Phen** release unit. Centers of the fluorene-dye and release unit are represented by green balls.

8. Quantum yield of uncaging of BEF-Phen-Ind

A solution of **BEF-Phen-Ind** (9 μM in H_2O, 3 mL, 80% pure sample by HPLC, absorption of the solution A = 0.5, ε = 90 000 M^{-1} cm^{-1}) was split into 2 portions: 1 mL was kept in the dark whilst 2 mL were photolyzed at 360 nm with Ryonet. Every minute 30 μL aliquots were taken from the photolyzed reaction mixture and the control sample, placed in vials and stored in liquid nitrogen. After 10 minutes of irradiation samples were analyzed one by one by HPLC with use of *HPLC method 1*. Release of 3-indolepropionic acid was monitored and quantified at 220 nm, while depletion of starting material **BEF-Phen-Ind** was quantified at 375 nm. Amount of released 3-indolepropionic acid was quantified with reference to the calibration curve that was prepared for concentrations 1.4–6.9 μM and 4.1–20.7 μM using *HPLC method 1*. Changes in concentration of **BEF-Phen-Ind** and **Ind** during the photolysis are presented in **Figure S40**. The monoexponential curves were fitted to the data and allowed for calculating rate constants of 3-indole propionic acid release and consumption of **BEF-Phen-Ind**. Obtained rate constant of **Ind** release (k_{IND} = 0.0019±0.0008 M^{-1} s^{-1}) was used in calculation of the quantum yield of uncaging according to the Equation S2. Uncaging quantum yield at 350 nm for **BEF-Phen-Ind** was determined as 0.0022.

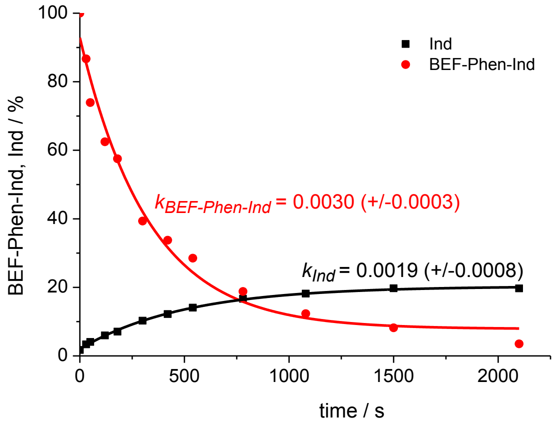

Figure S40. Photolysis of 9 μM solution of **BEF-Phen-Ind** in water. Changes in concentration of 3-indole propionic acid (black squares) and **BEF-Phen-Ind** (red circles) were determined with HPLC. Data was fitted to mono-exponential decay curve.

9. Literature references

S1 C. Gentilini, M. Boccalon and L. Pasquato, *Eur. J. Org. Chem.*, 2008, 3308–3313.
S2 C. Monnereau, E. Blart and F. Odobel, *Tetrahedron Lett.*, 2005, **46**, 5421–5423.
S3 V. Hrobáriková, P. Hrobarik, P. Gajdos, I. Fitilis, M. Fakis, P. Persephonis and P. Zahradnık, *J. Org. Chem.*, 2010, **75**, 3053–3068.
S4 C. B. Nielsen, M. Johnsen, J. Arnbjerg, M. Pittelkow, S. P. McIlroy, P. R. Ogilby and M. Jørgensen, *J. Org. Chem.*, 2005, **70**, 7065–7079.
S5 N. M. Yoon, C. S. Pak, H. C. Brown, S. Krishnamurthy and T. P. Stocky, *J. Org. Chem.*, 1973, **38**, 2786–2792.
S6 J. J. Michels, M. J. O'Connell, P. N. Taylor, J. S. Wilson, F. Cacialli and H. L. Anderson, *Chem. Eur. J.,* 2003, **9**, 6167–6176.
S7 O. Mongin, L. Porrès, M. Charlot, C. Katan and M. Blanchard-Desce*, Chem. Eur. J.,* 2007, **13**, 1481–1498.
S8 T. J. Donohoe, A. Ironmonger and N. M. Kershaw, *Angew. Chem. Int. Ed.*, 2008, **47**, 7314.
S9 M. S. DeClue, P.-A. Monnard, J. A. Bailey, S. E. Maurer, G. E. Collis, H.-J. Ziock, S. Rasmussen and J. M. Boncella, *J. Am. Chem. Soc.*, 2009, **131**, 931–933.
S10 T. Terasaka, T. Kinoshita, M. Kuno, N. Seki, K. Tanaka and I. Nakanishi, *J. Med. Chem.*, 2004, **47**, 3730–3743.
S11 A. Boccia, V. Lanzilotto, V. Di Castro, R. Zanoni, L. Pescatori, A. Arduinib and A. Secchi, *Phys. Chem. Chem. Phys.*, 2011, **13**, 4452–4462.
S12 Z. Li and R. Bittman, *J. Org. Chem.*, 2007, **72**, 8376–8382.
S13 S. Ito, I. Saito and T. Matsuura*, J. Am. Chem. Soc.*, 1980, **102**, 7535–7541.
S14 K. Lee and D. E. Falvey, *J. Am. Chem. Soc.*, 2000, **122**, 9361–9366.
S15 P. Wang, S. Liao, J.-B. Zhu, and Y. Tang, *Org. Lett.*, 2013, **15**, 3606–3609.
S16 R. S. Givens, A. Jung, C.-H. Park, J. Weber and W. Bartlett, *J. Am. Chem. Soc.*, 1997, **119**, 8369–8370.
S17 I. Aujard, C. Benbrahim, M. Gouget, O. Ruel, J.-B. Baudin, P. Neveu and L. Jullien, *Chem. Eur. J.,* 2006, **12**, 6865–6879.
S18 P. Chand, Y. S. Babu, S. Bantia, N. Chu, L. B. Cole, P. L. Kotian, W. G. Laver, J. A. Montgomery, V. P. Pathak, S. L. Petty, D. P. Shrout, D. A. Walsh and G. M. Walsh, *J. Med. Chem.*, 1997, **40**, 4030–4052.
S19 C. J. MacNevin, F. Atif, I. Sayeed, D. G. Stein and D. C. Liotta, *J. Med. Chem.,* 2009, **52**, 6012–6023.
S20 N. S. Makarov, M. Drobizhev and A. Rebane, *Optics Express*, 2008, **16**, 4029-4047
S21 J. D. Wilkinson, G. Wicks, A. Nowak-Krol, L. G. Lukasiewicz, C. J. Wilson, M. Drobizhev, A. Rebane, D. Gryko, T. and H. L. Anderson, *J. Mater. Chem. C*, 2014, **2**, 8.
S22 G. Papageorgiou and J. E. T. Corrie, *Tetrahedron*, 2007, **63**, 9668–9676.
S23 J. Goedhart and T. W. J. Gadella, *Biochemistry,* 2004, **43**, 4263–4271.
S24 H. J. Kuhn, S. E. Braslavsky and R. Schmidt, *Pure Appl. Chem.*, 1989, **61**, 187–210.
S25 M. Montalti, A. Credi, L. Prodi and M. T. Gandolfi, *Handbook of Photochemistry*, Taylor & Francis Group, Florida, 2006, p 601–604.
